# Systematic discovery of biomolecular condensate-specific protein phosphorylation

**DOI:** 10.1038/s41589-022-01062-y

**Published:** 2022-07-21

**Authors:** Sindhuja Sridharan, Alberto Hernandez-Armendariz, Nils Kurzawa, Clement M. Potel, Danish Memon, Pedro Beltrao, Marcus Bantscheff, Wolfgang Huber, Sara Cuylen-Haering, Mikhail M. Savitski

**Affiliations:** 1 Genome Biology Unit, European Molecular Biology Laboratory (EMBL), Heidelberg, Germany; 2 Cell Biology and Biophysics Unit, EMBL, Heidelberg, Germany; 3 Collaboration for joint PhD degree between EMBL and Faculty of Biosciences, Heidelberg University, Heidelberg, Germany; 4 European Bioinformatics Institute (EMBL-EBI), Hinxton, UK; 5 Cellzome, a GSK company, Heidelberg, Germany

**Keywords:** Post-translational modifications, Proteomics, RNA metabolism, Nuclear organization

## Abstract

Reversible protein phosphorylation is an important mechanism for regulating (dis)assembly of biomolecular condensates. However, condensate-specific phosphosites remain largely unknown, thereby limiting our understanding of the underlying mechanisms. Here, we combine solubility proteome profiling with phosphoproteomics to quantitatively map several hundred phosphosites enriched in either soluble or condensate-bound protein subpopulations, including a subset of phosphosites modulating protein–RNA interactions. We show that multi-phosphorylation of the C-terminal disordered segment of heteronuclear ribonucleoprotein A1 (HNRNPA1), a key RNA-splicing factor, reduces its ability to locate to nuclear clusters. For nucleophosmin 1 (NPM1), an essential nucleolar protein, we show that phosphorylation of S254 and S260 is crucial for lowering its partitioning to the nucleolus and additional phosphorylation of distal sites enhances its retention in the nucleoplasm. These phosphorylation events decrease RNA and protein interactions of NPM1 to regulate its condensation. Our dataset is a rich resource for systematically uncovering the phosphoregulation of biomolecular condensates.

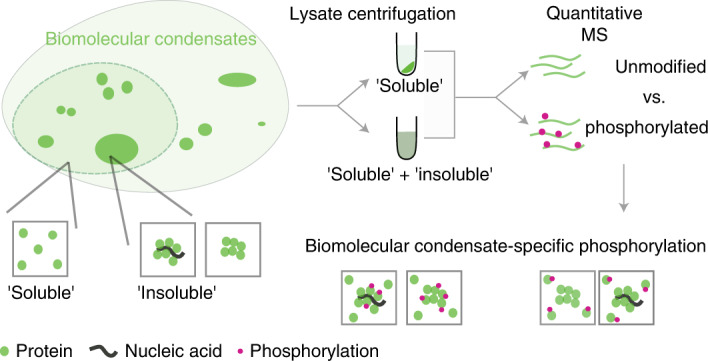

## Abstract (web summary)

A combination of solubility proteome profiling with phosphoproteomics enables systematic analysis of the phosphorylation status of proteins in soluble and condensate-bound pools.

## Main

Biomolecular condensates are macromolecular assemblies of proteins and nucleic acids that concentrate specific biomolecules while excluding others to perform specialized cellular functions^1–3^. Examples of such assemblies are membraneless organelles in the nucleus (including the nucleolus, nuclear speckles and Cajal bodies) and the cytosol (including stress granules and P-bodies)^3^. Many of these assemblies are formed by liquid−liquid phase separation of proteins and RNA^2,4^. In vitro reconstitution experiments have established that multivalent interactions between protein domains, intrinsically disordered regions and RNA are central to the formation of these condensates^5^. Our understanding of the intracellular mechanisms regulating the formation and dissolution of biomolecular condensates is still limited. Post-translational modification (PTM) of proteins is thought to be a major regulatory mechanism^6,7^. Protein phosphorylation is of major interest as its rapid and reversible addition in response to cellular cues can alter protein function, interactions and localization^8^.

Protein phosphorylation can promote as well as repress condensate formation. For example, in fused in sarcoma (FUS), an RNA-binding protein linked to neurodegenerative disorders, multi-phosphorylation of its N-terminal disordered segment prevents condensate formation^9^. In fragile X mental retardation protein (FMRP), which forms ribonuclear protein granules in neurons, multi-phosphorylation of its C-terminal disordered region increases condensation in vitro^10^. Phosphorylation sites that can influence protein condensation are known only for a few proteins. In these examples, the impact of phosphorylation on protein condensation driven by either homotypic interactions or protein–RNA oligonucleotide interactions was evaluated. However, it is increasingly recognized that several intracellular condensates form due to heterotypic interactions between different proteins and RNA^11,12^. Hence, to understand the consequences of phosphorylation on biomolecular condensates, we need to characterize the phosphorylation status of distinct subpopulations of proteins (condensate bound or soluble) on a systems level.

To address this, we combined phosphoproteomics^13^ with a recently developed quantitative proteomics-based approach to measure protein solubility following a lysate-centrifugation assay^14^ to determine the phosphorylation sites that are observed in either the soluble or the condensate-bound subpopulation of proteins. We identify known examples of phosphorylation-mediated regulation of a protein (dis)association with a biomolecular condensate and uncover several hundred phosphosites that can potentially modulate condensate dynamics. These phosphosites occur in disordered regions of proteins with distinct biases in hydrophobicity and charged residue distribution. Taking two proteins involved in different aspects of RNA metabolism, HNRNPA1 and NPM1, as examples, we identify driver phosphorylation events and elucidate the mechanism of phosphoregulation of their condensation.

## Results

### Solubility status of the human proteome

**Fig. 1:**
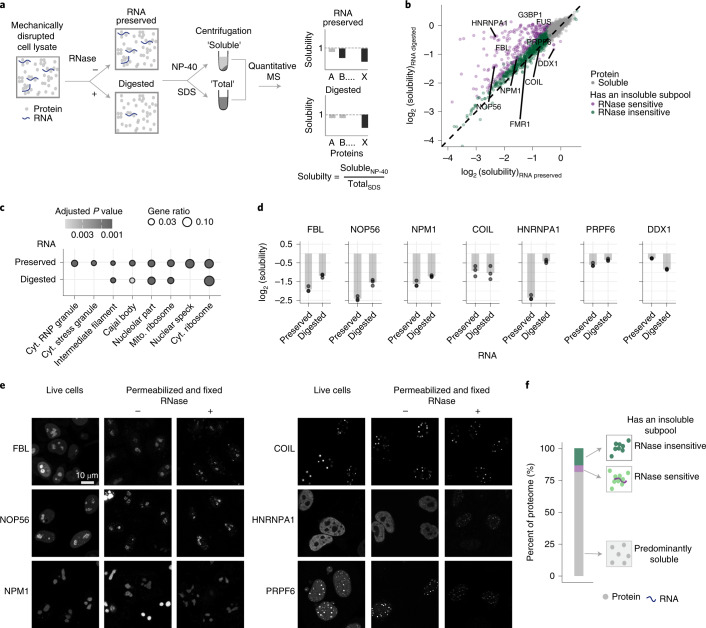
Solubility status of the human proteome. **a**, Experimental setup of solubility proteome profiling using RNA-preserved and RNA-digested crude cellular lysate systems. **b**, Scatterplot comparing the solubility (NP-40/SDS ratio) of proteins in RNA-preserved (*x* axis) and RNA-digested (*y* axis) samples in log_2_ scale. Proteins that maintain a significant insoluble subpopulation (see Methods for statistical significance) in both lysate types are depicted in green and proteins that alter solubility due to cellular RNA digestion are shown in purple. FMR1, fragile X messenger ribonucleoprotein 1; G3BP1, G3BP stress granule assembly factor 1. **c**, Dot plot showing a subset of over-represented gene ontology cellular compartment terms (*q* value < 0.05, hypergeometric test, corrected using the Benjamini–Hochberg procedure) among proteins that exhibit low solubility in RNA-preserved and RNA-digested lysates. Cyt., cytosolic; mito., mitochondrial. **d**, Bar plot representation of solubility (*y* axis in log_2_ scale) of FBL, NOP56, NPM1, COIL, HNRNPA1 and PRPF6 in RNA-preserved and RNA-digested (*x* axis) samples. Dots represent the solubility measurement from three independent biological replicates. Low FCs represent low solubility. **e**, Confocal microscopy images of HeLa cells overexpressing fusion proteins GFP–FBL, GFP–NOP56, SiR-SNAP–NPM1, GFP–COIL, GFP–HNRNPA1 and GFP–PRPF6 in live cells and in permeabilized (without and with RNase treatment) and fixed cells. **f**, Bar plot representing different solubility classes of proteins. Proteins are classified as ‘predominantly soluble’ (no significant insoluble subpool was measured) and ‘has an insoluble subpool’, which is either ‘RNase sensitive’ or ‘RNase insensitive’. Source data

**Extended Data Fig. 1:**
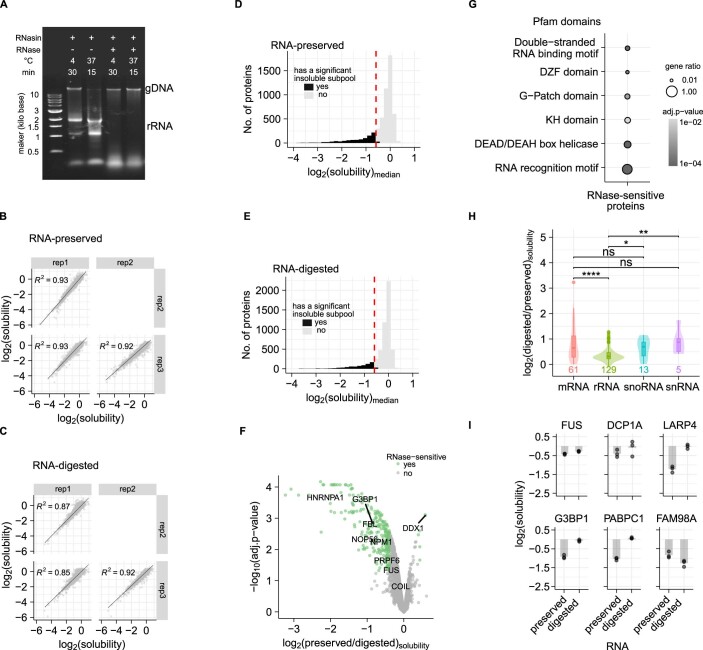
Experimental setup and classification of proteins based on solubility. (**a**) 1% agarose gel separation of total nucleic acids extracted under selectively preserving and digesting cellular RNA under different conditions. gDNA: genomic DNA, rRNA: ribosomal RNA. (**b**, **c**) Scatter plot comparing the reproducibility of protein solubility measurements (in log_2_ scale) from three independent replicates from (**B**) RNA-preserved and (**C**) RNA-digested lysate. (**d**, **e**) Histogram of proteome-wide solubility of proteins in (**D**) RNA-preserved and (**E**) RNA digested lysate. Proteins that exhibit at least 30% lower abundance in NP40 compared to SDS-extracted lysate at FDR < 1% (Benjamini-Hochberg procedure) was considered to maintain an insoluble subpool in the lysate. (**f**) Differential solubility of proteins in RNA-preserved and RNA-digested lysate. The y-axis represents -log_10_(adjusted p-value) (*limma*, corrected with Benjamini-Hochberg procedure) and the x-axis displays the log_2_(fold change). Green dots represent proteins that exhibit | log_2_(fold change) | > 0.5, at FDR < 1%. (**g**) Dot plot showing the over-represented Pfam protein domain among proteins that exhibit significant difference in solubility in RNA-digested compared to RNA-preserved lysate (q-val < 0.05, hypergeometric test, corrected using Benjamini-Hochberg procedure). (**h**) Boxplot with violin plot showing the distribution of difference in solution of proteins after RNA digestion (compared to preserving RNA in lysate) among proteins annotated to be binding to mRNA, rRNA, snoRNA and snRNA). Significance calculated using Wilcoxon signed-rank test (two-sided) and represented as ns: not significant, *p < 0.05, **p < 0.01, and ***p < 0.001. The box plots display the median and IQR, with the upper whiskers extending to the largest value ≤1.5 × IQR from 75th percentile and the lower whiskers extending to smallest values ≤1.5 × IQR from 25th percentile (**i**) Bar plot representation of solubility (y-axis in log_2_ scale) of FUS, G3BP1, PABPC1, DCP1A, LARP4 and FAM98A in RNA-preserved and RNA-digested (x-axis). Dots represent the solubility measurement from three independent biological replicates. Low fold-changes represent low solubility. Source data

To map the distinct protein subpopulations of the human proteome, we measured proteome-wide solubility of proteins from mechanically disrupted HeLa cells after preserving RNA (termed ‘RNA-preserved’) or digesting cellular RNA (‘RNA-digested’; Extended Data Fig. 1a) with an RNase cocktail (RNase A, RNase T1 and RNase H). An aliquot of these lysates was extracted with a mild detergent (NP-40) that solubilizes cellular and organelle membranes while preserving higher-order assemblies of proteins and nucleic acids, which are subsequently removed using high-speed centrifugation. A second aliquot of the lysate was extracted with a strong detergent (SDS), which denatures and solubilizes the entire proteome (Fig. 1a). The ratio of NP-40-derived and SDS-derived protein abundances is representative of its extent of solubility: smaller ratios suggest a higher proportion of a protein maintained in an insoluble subpopulation.

Using quantitative MS-based proteomics (Fig. 1a)^15^, we measured the abundance of 5,398 proteins (with at least two unique peptides in all three replicates; Extended Data Fig. 1b,c) from NP-40-solubilized and SDS-solubilized RNA-preserved and RNA-digested lysates. Proteins with at least 30% lower abundance and an adjusted *P* value <0.01 in NP-40-derived proteomes compared to SDS-derived proteomes were considered to maintain an insoluble subpool, hence referred to as the ‘insoluble proteome’ (Extended Data Fig. 1d,e and Supplementary Data 1). The solubilities (NP-40/SDS ratio) of proteins in both lysate types were comparable (Fig. 1b), with the exception of 284 proteins (Extended Data Fig. 1f). Among these, 278 proteins gained solubility upon digestion of cellular RNA (Fig. 1b). The majority (>80%) of these proteins have an RNA-binding domain (Extended Data Fig. 1g), explaining their RNase-sensitive solubility profile. The insoluble proteome of both lysate types mainly consists of proteins annotated as being part of different biomolecular condensates, along with a small proportion of cytoskeletal proteins (including actin and lamin) (Fig. 1c). However, proteins annotated to be part of cytoplasmic stress granules, nuclear speckles and/or cytoplasmic ribonuclear proteins lost their insoluble subpools upon RNA digestion (Fig. 1c).

**Extended Data Fig. 2:**
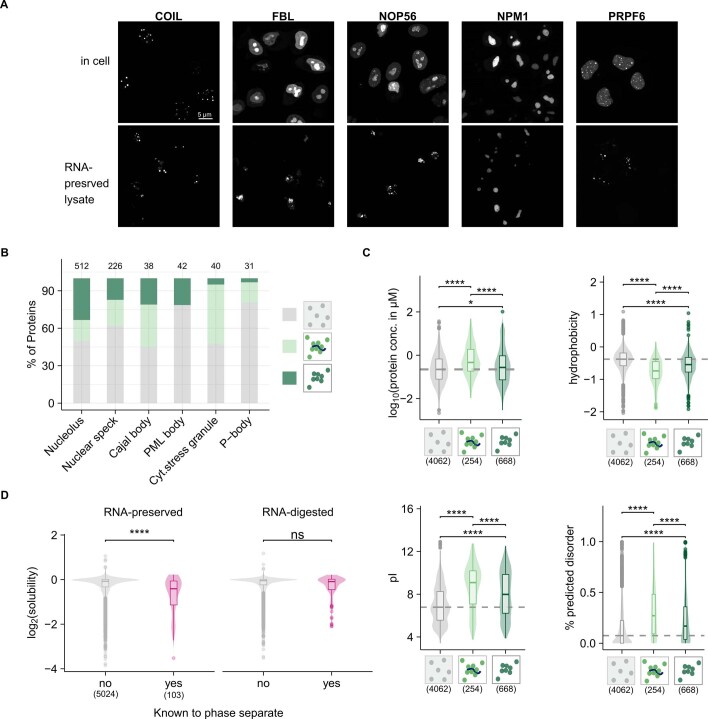
Physiochemical properties of proteins in different solubility subgroups. (**a**) Representative confocal images of intact HeLa cells expressing GFP-tagged COIL, FBL, NOP56, NPM1 and PRPF6 and post-lysis using conditions used for proteomics assay. (**b**) Bar plot representing the proportion of proteins of different solubility classes present among proteins that are annotated to be part of various membrane-less organelles. Gene ontology annotation only based on experimental evidence was used for binning the proteins in different cellular compartments. Number of proteins from each organelle is shown on the top. (**c**) Distribution of intracellular protein concentration (top left, in log_10_ scale), hydrophobicity (top right, Kyte Doolittle scale), isoelectric point (pI, bottom left) and %predicted disorder in the sequence (bottom right) of proteins that were classified as ‘predominantly soluble’, and has an insoluble sub-pool that is ‘RNase-sensitive’ or ‘RNase-insensitive’. The box plots display the median and IQR, with the upper whiskers extending to the largest value ≤1.5 × IQR from 75th percentile and the lower whiskers extending to smallest values ≤1.5 × IQR from 25th percentile. Numbers represent the number of proteins in each category. Significance calculated using Wilcoxon signed-rank test (two-sided) and represented as ns: not significant, *p < 0.05, **p < 0.01, and ***p < 0.001. (**d**) Distribution of solubility (in log_2_ scale) of proteins that are known to undergo phase separation based on *in-vitro* experiments (curated list from PhaseDB) in RNA-preserved (left) and RNA-digested (right) lysate. Numbers represent the number of proteins in each category. Significance calculated using Wilcoxon signed-rank test (two-sided) and represented as ns: not significant, *p < 0.05, **p < 0.01, and ***p < 0.001. Source data

The extent of gain in solubility after RNA digestion was highly variable (Fig. 1b). Solubilities of proteins annotated to interact with mRNA exhibited higher susceptibility to RNase treatment than ribosomal RNA (rRNA)-binding proteins (Extended Data Fig. 1h). For example, nucleolar proteins such as fibrillarin (FBL), NPM1 and NOP56 exhibited a mild increase, while RNA-splicing proteins such as HNRNPA1 and pre-mRNA-processing factor (PRPF)6 were completely solubilized, and Cajal body protein, coilin (COIL) remained unaffected upon digestion of RNA (Fig. 1d and Extended Data Fig. 1i). These solubility effects were recapitulated for fluorescently (GFP- or SNAP-)tagged versions of the above-mentioned proteins using confocal microscopy. In live cells, most proteins appeared as condensates (Fig. 1e) as well as the soluble nucleoplasmic pool. Permeabilization (using the same lysis buffer as in the proteomic assay) followed by fixation retained the protein signals only in the condensates but not in the soluble pool within the nucleoplasm. Permeabilization with RNase-containing buffer reduced the size of the condensates to varying degrees, matching observations from the MS-determined solubility profiles (Fig. 1d,e). Furthermore, the fluorescently tagged proteins remained in condensates in cell lysates prepared using the lysis buffer used for the proteomic assay (Extended Data Fig. 2a). These observations suggest that the lysis conditions used for the proteomic assay permeabilize the nucleus, providing access to the soluble protein pool while preserving the condensate-bound subfraction, which is read out as insoluble.

Strikingly, we also observed that six proteins forming a transcription-dependent RNA-transport complex (consisting of C14ORF166, DDX1, FAM98A, FAM98B and RTCB) decreased in solubility after digestion of RNA (Fig. 1b,d and Extended Data Fig. 1i). This complex shuttles between the nucleus and the cytosol^16^ and is known to be sequestered into stress granules^17^ during transcriptional arrest. Our data suggest that cellular RNA keeps these proteins soluble and prevents partitioning into condensates.

The human proteome can thus be classified into proteins that are predominantly soluble (81.5%) or maintain an insoluble subpool that is either RNase sensitive (5.5%) or RNase insensitive (13.5%) (Fig. 1f and Supplementary Data 2). The proportion of these solubility subgroups was highly variable among protein sets annotated to be part of different biomolecular condensates (Extended Data Fig. 2b). Proteins that maintained an insoluble subpool tended to have higher intracellular protein concentrations, lower hydrophobicity, higher positive charge and higher percentages of predicted structural disorder (Extended Data Fig. 2c and Supplementary Data 2) than proteins that were predominantly soluble. These characteristics are reminiscent of proteins that undergo liquid–liquid phase separation^2^. The small number of proteins that are known to phase separate in vitro (*n* = 103)^18^ exhibited low solubility in the RNA-preserved lysate (Extended Data Fig. 2d), suggesting that this lysate maintains higher-order assemblies of these proteins. In sum, proteome-wide solubility measurements report on distinct subpopulations of proteins associated with biomolecular condensates.

### Mapping phosphorylation sites of distinct protein pools

**Fig. 2:**
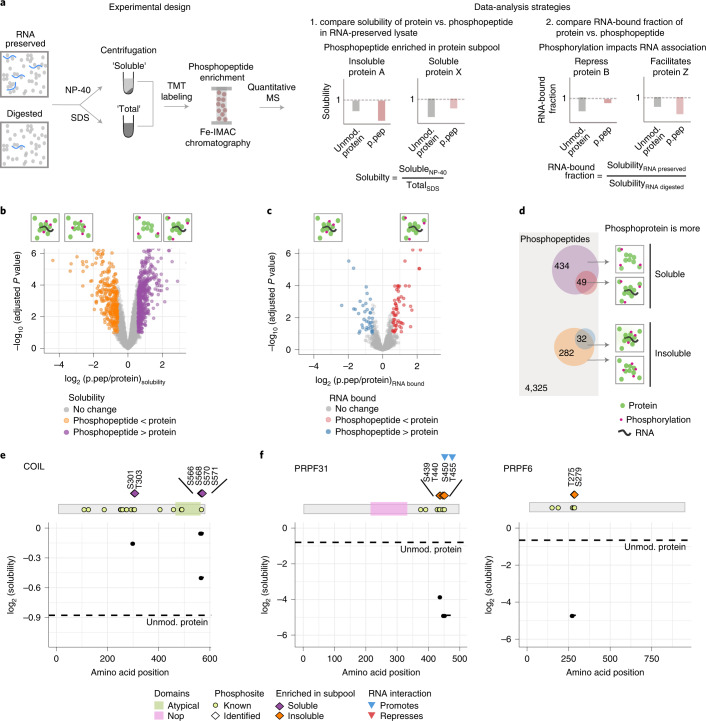
Mapping phosphorylation sites of distinct protein pools. **a**, Schematic representation of the experimental design and data-analysis strategies. Fe-IMAC, Fe^3+^-immobilized metal ion affinity chromatography; p.pep, phosphopeptide; TMT, tandem mass tag; unmod., unmodified. **b**, Volcano plot of the differential solubility of phosphopeptides of a protein compared to its unmodified protein in RNA-preserved lysate. Phosphopeptides exhibiting significantly (|log_2_ (FC)| > 0.5 and adjusted *P* value obtained from limma analysis (Benjamini–Hochberg) < 0.01) lower (orange) and higher (purple) solubility than the unmodified proteins are shown. **c**, Volcano plot of the differential RNA-bound fraction of phosphopeptides of a protein compared to its unmodified protein. Phosphopeptides exhibiting significantly (|log_2_(FC)| > 0.5 and adjusted *P* value obtained from limma analysis (Benjamini–Hochberg) < 0.01) lower (red) and higher (blue) proportions in the RNA-bound subpool than the unmodified proteins are shown. **d**, Venn diagram summarizing the overlap in different categories of assigned phosphopeptides. **e**,**f**, Visualization of the median solubility profiles (*n* = 3) of identified phosphopeptides (solid lines, phosphosites as points) and unmodified protein (dashed line) in log_2_ scale is represented along the linear sequence of the protein (*x* axis) of COIL (**e**), PRPF6 and PRPF31 (**f**). Top, schematic representation of the protein with its domains and known phosphosites from UniProt. Source data

**Extended Data Fig. 3:**
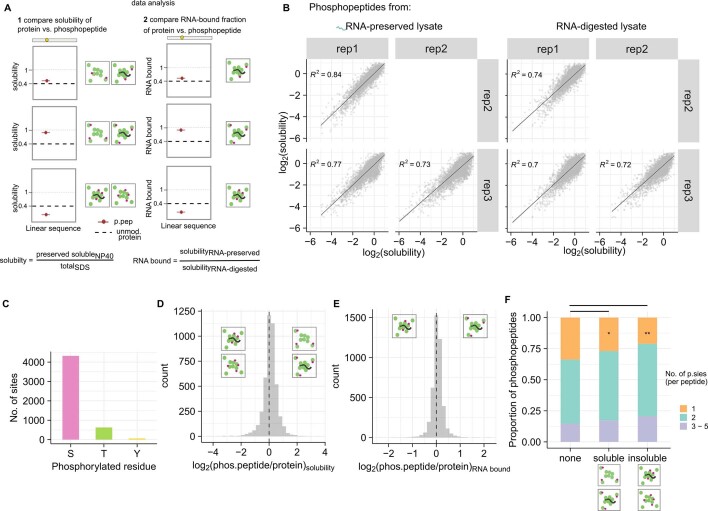
Differentially soluble phosphopeptides. (**a**) Schematic representation of data analysis and interpretation strategies of combining phosphoproteomics with solubility proteome profiling. (**b**) Scatter plot comparing the reproducibility of phosphopeptides solubility measurements (in log_2_ scale) from three independent replicates from RNA-preserved and RNA-digested lysate. (**c**) Bar plot representation of number of phosphorylated serine (S), threonine (T) and tyrosine (Y) residues identified in this dataset. (**d**) Histogram showing the difference in solubility of phosphopeptides and their corresponding unmodified proteins (x-axis in log_2_ scale) in RNA-preserved lysate. (**e**) Histogram showing the difference in RNA-bound fraction of phosphopeptides and their corresponding unmodified proteins (x-axis in log_2_ scale). (**f**) Barplot representing the proportion of single, double and multiple phosphorylation sites containing peptides among differenentially soluble phosphopeptides. Fisher’s exact test was utilized to ascertain the significance. ** p-value < 0.01, * p-value < 0.05. Source data

To identify phosphorylation patterns specific to distinct protein subpools, we combined solubility profiling with phosphoproteomics^13^ (Fig. 2a and Extended Data Fig. 3a). We measured the abundance of 7,026 phosphopeptides from three independent replicates of NP-40-solubilized and SDS-solubilized RNA-preserved and RNA-digested lysates (after filtering for stringent quality criteria; Methods and Extended Data Fig. 3b). These phosphopeptides mapped onto 5,011 distinct phosphorylation sites (86.3% S, 12.5% T and 1.2% Y; Extended Data Fig. 3c). The solubility of the phosphopeptides (NP-40/SDS ratio) was compared with the solubility of their respective unmodified proteins: most proteins are substoichiometrically phosphorylated and hence typically represent the unmodified state when no enrichment is performed. We observed 797 phosphopeptides with significantly lower (314 peptides, that is, phosphorylation enriched in the insoluble protein pool) or higher (483 peptides, that is, phosphorylation enriched in the soluble protein pool) solubility than their unmodified protein (|log_2_ (fold change (FC))| > 0.5, adjusted *P* value < 0.1; Fig. 2b, Extended Data Fig. 3d and Supplementary Data 3).

Next, we mapped the phosphorylation events that may affect the interaction of a protein with RNA. As digestion of cellular RNA resulted in a global increase in solubility of proteins (Fig. 1b), the ratio of protein solubility before and after RNA digestion reflects the fraction of a protein that was solubilized due to RNase treatment, with smaller values indicating a higher amount of protein associated with RNA. This ratio was termed the ‘RNA-bound fraction’ (Fig. 2a). We compared this ratio between phosphopeptides and their corresponding unmodified protein (Fig. 2c and Extended Data Fig. 3e). We observed 96 phosphopeptides with a significantly lower (58 peptides, that is, phosphorylation may repress RNA interaction) or higher (38 peptides, that is, phosphorylation may promote RNA association) RNA-bound fraction than their unmodified protein (|log_2_ (FC)| > 0.5, adjusted *P* value < 0.1; Fig. 2c and Supplementary Data 3). A majority of phosphorylation events that may reduce RNA association were also enriched in the soluble pool of proteins, while phosphorylation events that may facilitate RNA binding predominantly came from the insoluble pool of proteins (Fig. 2d).

**Extended Data Fig. 4:**
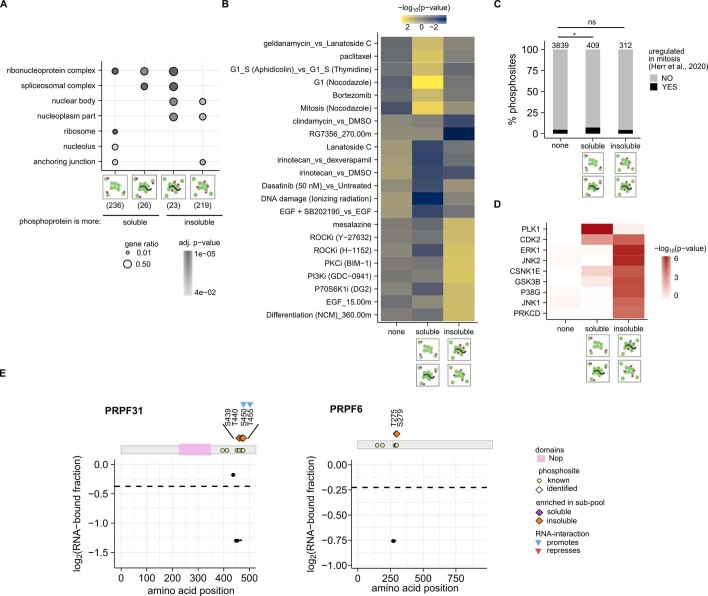
Regulation and localization of differential phosphosites. (**a**) Dot plot of gene ontology cellular compartments over-represented among proteins which have differentially soluble phosphopeptides (q-val < 0.05, hypergeometric test, corrected using Benjamini-Hochberg procedure). (**b**) Heat map representation of the degree of regulation of phosphosites sub-divided into protein solubility subgroups across different conditions. The up or down regulation of phosphosites was inferred from a large scale collection of previously published phosphoproteomics datasets^26^ on various conditions including drug/inhibitor treatment and different cellular states. Cellular conditions in which the phosphosites assigned in this study showed significant change (Z-test, |-log10(p-value)| > 2) in regulation in at least one solubility subgroup are shown. High positive and negative values indicate increased or decreased regulation of phosphosites in the indicated condition. (**c**) Bar plot representation of the proportion of mitotically upregulated phosphorylation (from Herr et a., 2020) sites among the differentially soluble phosphosites identified from this dataset. Significance estimated using Fisher’s exact test and coded as * p-value < 0.05, ns- not significant. (**d**) Heat map representation of kinase over-representation analysis based on enrichment of their direct substrates in different protein solubility sub-groups. Kinases enriched in at least one protein solubility sub-group are shown (q-value < 0.15, hypergeometric test corrected with Benjamini-Hochberg procedure). (**e**) Visualization of the median RNA-bound fraction (n = 3) of identified phosphopeptides and unmodified protein of PRPF6 and PRPF31. Top: schematic representation of the protein with its domains and known phosphosites from Uniprot is shown. Median RNA-bound fraction (of three independent measurements, y-axis) of phosphopeptides (solid lines with points representing the site) and unmodified protein (dotted line) in log_2_ scale is represented along the linear sequence of the protein (x-axis). Source data

The differential phosphopeptides, both increasing and decreasing in solubility, had an under-representation of monophosphorylated peptides, suggesting that proximate phosphorylation events are likely to have a higher impact on protein solubility (Extended Data Fig. 3f). The phosphopeptides (797 peptides) mapped onto 369 proteins. Most of these proteins localize to different biomolecular condensates (Extended Data Fig. 4a). The phosphosites enriched in the soluble protein subpool were also regulated in other cellular states^19,20^, including mitosis, following proteasome inhibition and in response to DNA damage (Extended Data Fig. 4b,c), which are known to affect protein condensation^21,22^. This subset of phosphosites is also enriched in cyclin-dependent kinase 2 (CDK2) and Polo-like kinase 1 (PLK1) substrates^23^ (Extended Data Fig. 4d). Phosphosites specific to the insoluble protein pool were enriched in substrates of several kinases including casein kinase (CSNK1E) and protein kinase C-δ (PRKCD) (Extended Data Fig. 4d). These observations suggest that the dataset encompasses phosphorylation events that are relevant in various cellular processes and can regulate the (dis)association of proteins to different biomolecular condensates under steady-state conditions.

Our data encompass some of the known examples of phosphoregulation of protein condensates. For example, residues S301, T303, S566, S568, T570 and S571 of COIL had higher solubility than the unmodified protein (Fig. 2e and Supplementary Data 3 and 4), in agreement with previously known phosphosites (S566, S568, T570, S571, S572 and S573) that reduce the protein’s association with Cajal bodies^24^. Another example was the phosphorylation of spliceosome complex B proteins PRPF31 (at S439, T440, S450 and T455) and PRPF6 (at T275 and S279), which displayed lower solubility (Fig. 2f). Phosphorylation sites S450 and T455 of PRPF31 were enriched in the RNA-bound fraction of the protein (Extended Data Fig. 4e) and are known to stabilize the PRPF6–PRF31 interaction with U4 and U6 small nuclear RNA and other spliceosome proteins, which is crucial for the catalytic activity of spliceosomes^25^. Overall, the combination of phosphoproteomics with solubility profiling enabled the identification of phosphorylation sites that are specifically enriched in different protein subpopulations.

### Sequence properties of differential phosphorylation sites

**Fig. 3:**
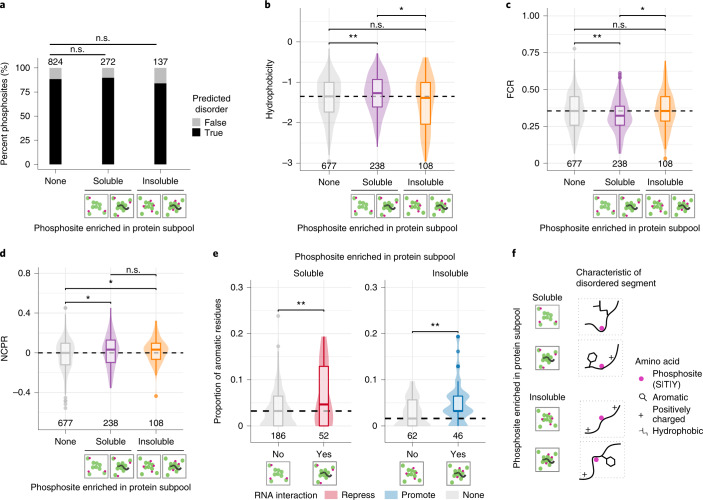
Sequence properties of disordered segments surrounding solubility subpopulation-specific phosphosites are distinct. **a**, Bar plot showing the proportion of phosphosites localized within the predicted disorder segments of proteins. Significance values were obtained using Fisher’s exact test; n.s., *P* > 0.05. **b**–**d**, Comparison of different sequence properties of 31-amino acid segment non-changing, soluble and insoluble subpool-enriched phosphosites (which were disordered based on Uversky classification). **b**, Hydrophobicity was calculated using the Kyte–Doolittle scale. **c**, The fraction of charged residues (FCR) was calculated as the sum of the fraction of positively charged (*f*_+_) and negatively charged (*f*_−_) residues. **d**, Net charge per residue (NCPR) was calculated as the difference between *f*_+_ and *f*_−_. The number of phosphosites in each category is indicated at the bottom of the representation. Significance was calculated using two-sided Wilcoxon signed-rank tests and is represented by *P < 0.05, **P < 0.01 and ***P < 0.001. The box plots display the median and the interquartile range (IQR), with the upper whiskers extending to the largest value ≤1.5 × IQR from the 75th percentile and the lower whiskers extending to the smallest values ≤1.5 × IQR from the 25th percentile. **e**, Comparison of the proportion of aromatic amino acids in the 31-amino acid segments of phosphosites, which are enriched in either the soluble (right) or insoluble (left) protein subpool and may or may not impact RNA interactions. Significance was calculated using a two-sided Wilcoxon signed-rank test and is represented by *P < 0.05, **P < 0.01 and ***P < 0.001. The box plots display the median and the IQR, with the upper whiskers extending to the largest value ≤1.5 × IQR from the 75th percentile and the lower whiskers extending to the smallest values ≤1.5 × IQR from the 25th percentile. The number of phosphosites in each category is indicated at the bottom of the representation. **f**, Schematic representation of key sequence properties observed in phosphosites that are enriched in soluble and insoluble subpools of proteins. Source data

**Extended Data Fig. 5:**
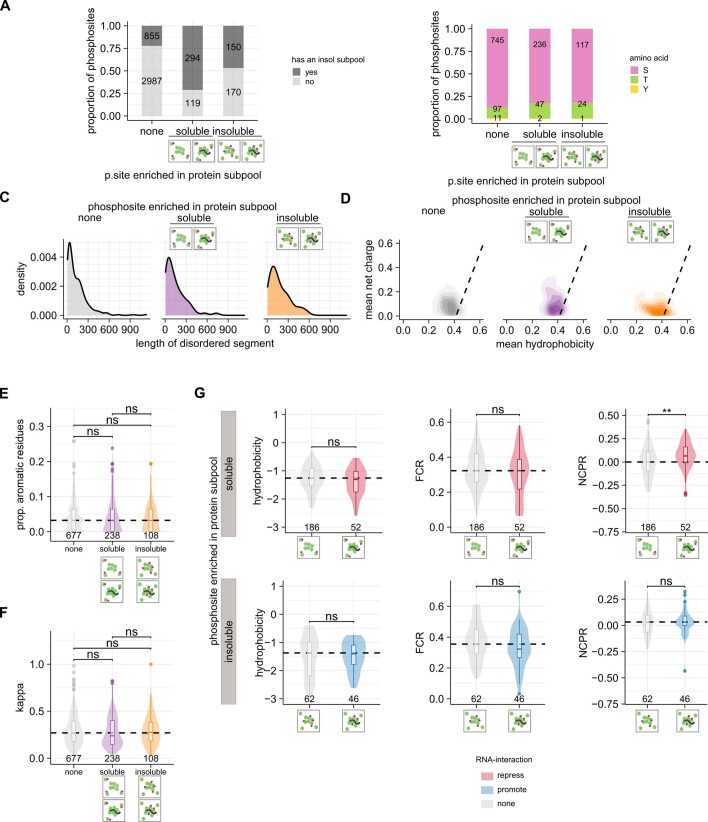
Sequence features of differentially soluble phosphopeptides. (**a**) Bar plot representing the proportion of classified phosphosites that correspond to proteins which have a significant insoluble sub-pool. Only phosphosites identified from proteins that maintains a significant insoluble sub-pool are used for subsequent analysis. (**b**) Number of phosphorylated serine (S), threonine (T) and tyrosine (Y) among classified phosphosites of protein which have a significant insoluble sub-pool. (**c**) Density plot representing the distribution of predicted disorder segment lengths in proteins with a significant insoluble sub-pool and an identified phosphosite. (**d**) 2D-density plot of charge vs. hydrophobicity of 31-amino acid segment surrounding the phosphosite (as center residue). Dotted line is the boundary (mean net charge = 2.785 X mean hydrophobicity – 1.151) that is shown to distinguish disordered (left of the line) from folded (right of the line) segments based on Uversky classification. (**E**) Distribution of proportion of aromatic residues (F|W|Y) and (**f**) Kappa value of the 31-amino acid segments (which were disordered based on Uversky classification) of different solubility subgroups. The number of phosphosites in each category is indicated at the bottom of the representation. Significance calculated using two-sided Wilcoxon signed-rank test and represented as *p < 0.05, **p < 0.01, and ***p < 0.001. The box plots display the median and IQR, with the upper whiskers extending to the largest value ≤1.5 × IQR from 75th percentile and the lower whiskers extending to smallest values ≤1.5 × IQR from 25th percentile. (**g**) Distribution of hydrophobicity, net charge per residue (NCPR) and fraction of charged residue (FCR) between phosphosites that may impact protein solubility or affect solubility through alteration of RNA-binding properties of proteins. The number of phosphosites in each category is indicated at the bottom of the representation. Significance calculated using two-sided Wilcoxon signed-rank test and represented as *p < 0.05, **p < 0.01, and ***p < 0.001. The box plots display the median and IQR, with the upper whiskers extending to the largest value ≤1.5 × IQR from 75th percentile and the lower whiskers extending to smallest values ≤1.5 × IQR from 25th percentile. Source data

To gain insights into how the mapped phosphosites (Extended Data Fig. 5a) impact solubility transitions, we assessed their sequence features. Similar proportions of S, T and Y sites were mapped among phosphosites enriched in soluble and insoluble pools of proteins (Extended Data Fig. 5b). Similar to most known phosphosites^26^, these sites preferentially localized to predicted disordered regions of proteins (Fig. 3a). Due to high variability in the length of these predicted disordered segments (Extended Data Fig. 5c), we assessed the different molecular features of a 31-amino acid window surrounding the site (±15 amino acids, with the phosphosite as the center). Most (>95%) of the 31-amino acid segments were also disordered (based on Uversky classification^27^; Extended Data Fig. 5d), encompassing high proportions of charged amino acids and low proportions of hydrophobic amino acids. However, the disordered segments of phosphosites enriched in the soluble protein subpool were more hydrophobic (Fig. 3b) and had a lower number of charged residues (Fig. 3c), with a net positive charge (Fig. 3d) compared to the non-changing sites. The disordered segments of phosphosites enriched in the insoluble protein subpool had a similar distribution of hydrophobic (Fig. 3b) and charged residues (Fig. 3c), while carrying a higher net positive charge (Fig. 3d) compared to the non-changing sites. No discernable differences in the segregation of oppositely charged residues (*κ*)^28^ or the proportion of aromatic residues (Extended Data Fig. 5e,f) was observed between different solubility subgroups (Extended Data Fig. 5b).

Hydrophobicity and the fraction of charged residues of the disordered segments remained indistinguishable between sites that potentially impact RNA binding and solubility (Extended Data Fig. 5g). However, two distinguishing features between sites that can influence RNA binding among the solubility subgroup-specific sites were observed: phosphosites that may repress RNA binding to increase solubility had more positive charges (Extended Data Fig. 5g) and the proportion of aromatic amino acids around the RNA interaction-promoting and RNA interaction-repressing sites was significantly higher (Fig. 3e).

In summary, the differentially soluble phosphosites are located in disordered segments of proteins with significant differences in hydrophobicity and charge (Fig. 3f).

### Phosphorylation affects HNRNPA1 condensation

**Fig. 4:**
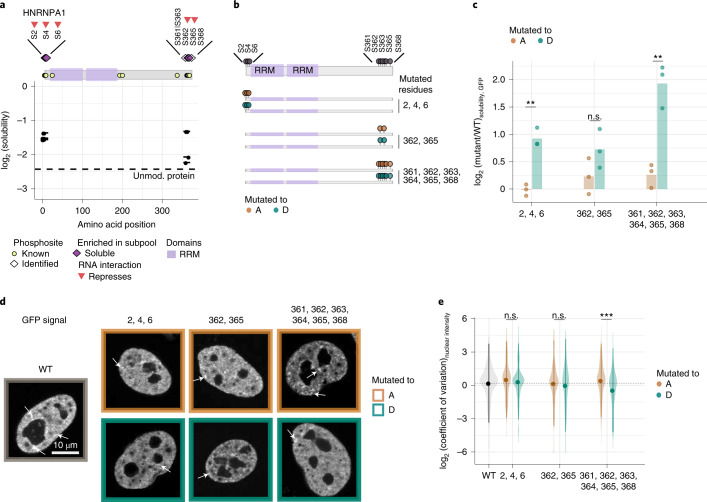
Multisite phosphorylation of the HNRNPA1 C terminus impacts its solubility. **a**, Visualization of the solubility profiles of identified phosphopeptides of HNRNPA1 and unmodified HNRNPA1 protein. Top, schematic representation of the protein with its domains and known phosphosites from UniProt is shown. Median solubility (of three independent measurements, *y* axis) of phosphopeptides (solid lines with points representing the sites) and unmodified protein (dashed line) in log_2_ scale is represented along the linear sequence of the protein (*x* axis). **b**, Schematic representation of different phosphodeficient (S to A) and phosphomimetic (S to D) mutants of HNRNPA1. These variants were expressed as GFP-tagged fusion proteins. RRM, RNA-recognition motif. **c**, Bar plot of the solubility (*y* axis) of GFP-tagged phosphodeficient and phosphomimetic mutants (sites are indicated on the *x* axis) of HNRNPA1 normalized to that of GFP-tagged WT protein is shown. Points represent the size effect calculated from three independent biological replicates. The variants of HNRNPA1 were expressed as GFP fusion proteins. Hence, the solubility of the tag (GFP) is used as the proxy to infer the solubility of HNRNPA1 variants. Mean from three independent trials are shown and the statistical significance was obtained by comparing the phosphodeficient and phosphomimetic mutant pairs using Student’s *t*-test (two sided) and is represented by *P < 0.05, **P < 0.01 and ***P < 0.001. **d**, Representative examples of a HeLa cell line transiently expressing the GFP-tagged HNRNPA1 mutant proteins depicted in **b**. GFP signal of WT in gray (left), phosphodeficient mutant proteins in brown (top) and phosphomimetic mutant proteins in green (bottom). Single *z* slices are shown. Scale bar, 10 µm. Examples of nuclear clusters are indicated with arrows. **e**, Coefficient of variation (s.d. ÷ mean intensity) of the nuclear signal of GFP-tagged WT and variants of HNRNPA1. Violin plot displays the underlying distribution of the coefficient of variation calculated from at least 120 nuclei from two independent experiments. The mean and s.d. are represented as a point and solid lines. Statistical significance was obtained using Student’s *t*-test (two sided) and is represented by *P < 0.05, **P < 0.01 and ***P < 0.001. Source data

**Extended Data Fig. 6:**
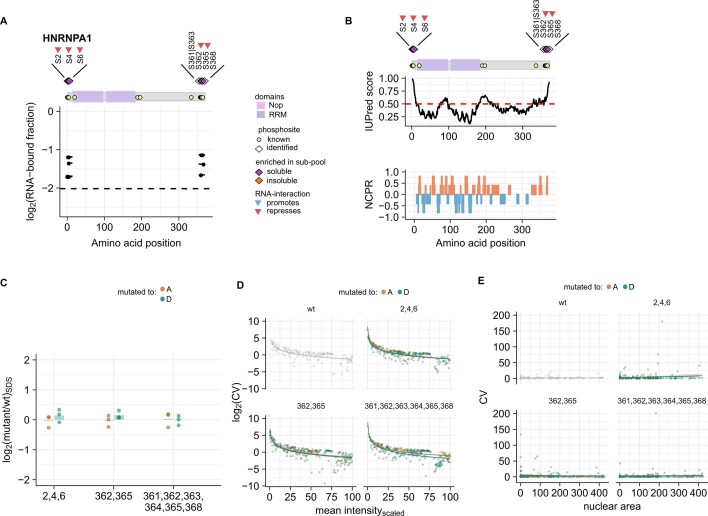
Phosphoregulation of HNRNPA1. (**a**) Visualization of the RNA-bound fraction of identified phosphopeptides and unmodified protein of HNRNPA1. Top: schematic representation of the protein with its domains and known phosphosites from Uniprot is shown. Median RNA-bound fraction (of three independent measurements, y-axis) of phosphopeptides (solid lines with points representing the site) and unmodified protein (dotted line) in log_2_ scale is represented along the linear sequence of the protein (x-axis). (**b**) IUpred, prediction of intrinsic disorder (top) and net charge per residue (NCPR, calculated over 5 aa window) along the linear sequence of HNRNPA1. (**c**) Barplot representing the relative protein abundance (in log_2_ scale, y-axis) of heterologously expressed GFP-tagged phosphomutants (x-axis) of HNRNPA1 (proxy for intracellular protein expression) compared to the wild type (wt) from three independent biological replicates. (**d**) Comparison of coefficient of variation (CV, in log_2_ scale) of intensity with the mean intensity of (segmented-) nucleus (n > 100) from two independent trials. (**f**) Comparison of coefficient of variation (CV) of intensity with the area of the (segmented-) nucleus (n > 100) from two independent trials. Source data

Next, we examined the impact of phosphorylation on the condensation propensity of HNRNPA1, a key RNA-splicing factor. Phosphopeptides spanning the N and C termini (N-terminal sites S2, S4 and S6 and C-terminal sites S362 and S365 and the ambiguous location in S361|S363|S364|S368) of HNRNPA1 exhibited higher solubility (Fig. 4a and Supplementary Data 3 and 4) than the overall protein solubility. These phosphosites were also low in the RNA-bound fraction of the protein, suggesting their role in repressing RNA binding (Extended Data Fig. 6a). The N-terminal sites are in a negatively charged intrinsically disordered segment, while the C-terminal sites occur in a positively charged disordered segment of HNRNPA1 (Extended Data Fig. 6b). Phosphosites S4 and S6 are known to repress HNRNPA1 interaction with RNA in the cytoplasm^29^, and the C-terminal sites (residues 360–365 and 368) are known to relocate the protein to the cytoplasm during osmotic stress^30^.

To assess the importance of multi-phosphorylation in HNRNPA1 associations, we built three sets of phosphodeficient (S to A) and phosphomimetic (S to D) mutants of HNRNPA1. While such mutants do not copy the exact roles of a loss or gain of phosphorylation, they provide a close approximation to assess the phosphorylation effect. The first mutant had three point mutations on N-terminal sites (S2, S4 and S6). The second mutant had two point mutations on C-terminal sites (S362 and S365). The third mutant had six point mutations in the C terminus of the protein, which included all proximate sites of S362 and S365, namely, S361, S363, S364 and S368, as there was ambiguity in the site localization (Supplementary Data 3). MS-based readouts typically struggle to identify and assign the location of highly phosphorylated peptides with putative sites located next to each other^31^. Hence, one mutant version of HNRNPA1 that encompassed all proximate residues of S362 and S365 was included (Fig. 4b). Solubility profiling of HeLa cells transiently overexpressing wild-type (WT) or mutant HNRNPA1 showed that the phosphomimetic versions of HNRNPA1 exhibited higher solubility than the respective deficient versions (Fig. 4c and Extended Data Fig. 6b). Confocal imaging of these cells showed that, despite all mutant proteins being predominantly located within the nucleus (similar to the WT protein; Fig. 4d), the heterogeneity of the fluorescent intensity within the nucleus (measured by the coefficient of variation) of the phosphomimetic mutant harboring six mutations (361, 362, 363, 364, 365 and 368) was lower than that of its deficient (Fig. 4e and Extended Data Fig. 6c,d) version. This observation suggested that multisite phosphorylation of HNRNPA1 at its C terminus reduces the protein’s propensity to form nuclear clusters.

### Phosphorylation impacts NPM1 localization to the nucleolus

**Fig. 5:**
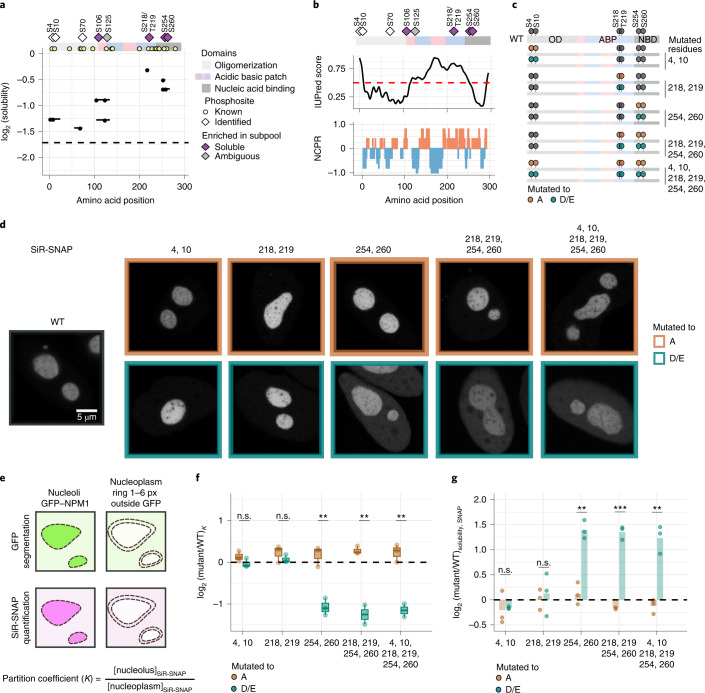
Phosphorylation of S254 and S260 are crucial for NPM1 localization. **a**, Visualization of the solubility profiles of identified phosphopeptides of NPM1 (solid lines, phosphosites as points) and unmodified NPM1 protein (dashed line). Top, schematic representation of the different protein domains and previously known phosphosites are shown. **b**, IUPred, prediction of intrinsic disorder (top) and net charge per residue (calculated over a five-amino acid window, bottom) along the linear sequence of NPM1. **c**, Schematic representation of different phosphodeficient (S|T to A) and phoshomimetic (S to D|T to E) mutants of NPM1. ABP, acidic basic patch; NBP, nucleic acid binding; OD, oligomerization domain. **d**, Representative examples of a HeLa cell line overexpressing WT GFP-tagged NPM1 from a bacterial artificial chromosome (BAC) and transiently expressing the SNAP-tagged NPM1 mutant proteins depicted in **c**. SiR-SNAP signal of WT is in dark gray (left), phosphodeficient mutants are in brown (top), and phosphomimetic mutants are in green (bottom). Single *z* slices are shown. Scale bar, 5 µm. **e**, Schematic of nucleolus and nucleoplasmic segmentation. WT GFP–NPM1 was used for nucleolus segmentation, and a rim surrounding each nucleolus was used for the nucleoplasmic segmentation. SiR-SNAP signals were quantified. The partition coefficient (*K*) is the proportion of nucleolar intensity to nucleoplasmic intensity. px, pixels. **f**, Box plot of relative *K* values (*y* axis) of SNAP-tagged NPM1 mutants (*x* axis) with respect to SNAP-tagged NPM1 WT from at least three independent trials is shown in log_2_ scale. Dashed lines represent the effect size of SNAP-tagged NPM1 WT (*n* ≥ 3). Statistical significance was obtained by comparing the phosphodeficient and phosphomimetic mutant pairs using Student’s *t*-test (two-sided) and is represented by *P < 0.05, **P < 0.01 and ***P < 0.001. The box plots display the median and the IQR, with the upper whiskers extending to the largest value ≤1.5 × IQR from the 75th percentile and the lower whiskers extending to the smallest values ≤1.5 × IQR from the 25th percentile. **g**, Bar plot of mean protein solubility (*y* axis, from three independent trials) of SNAP-tagged NPM1 mutants (*x* axis) with respect to SNAP-tagged NPM1 WT measured using the proteomic assay. Points represent the solubility measured from *n* = 3 experiments. Statistical significance was obtained by comparing the phosphodeficient and phosphomimetic mutant pairs using Student’s *t*-test (two-sided) and is represented by *P < 0.05, **P < 0.01 and ***P < 0.001. Source data

**Extended Data Fig. 7:**
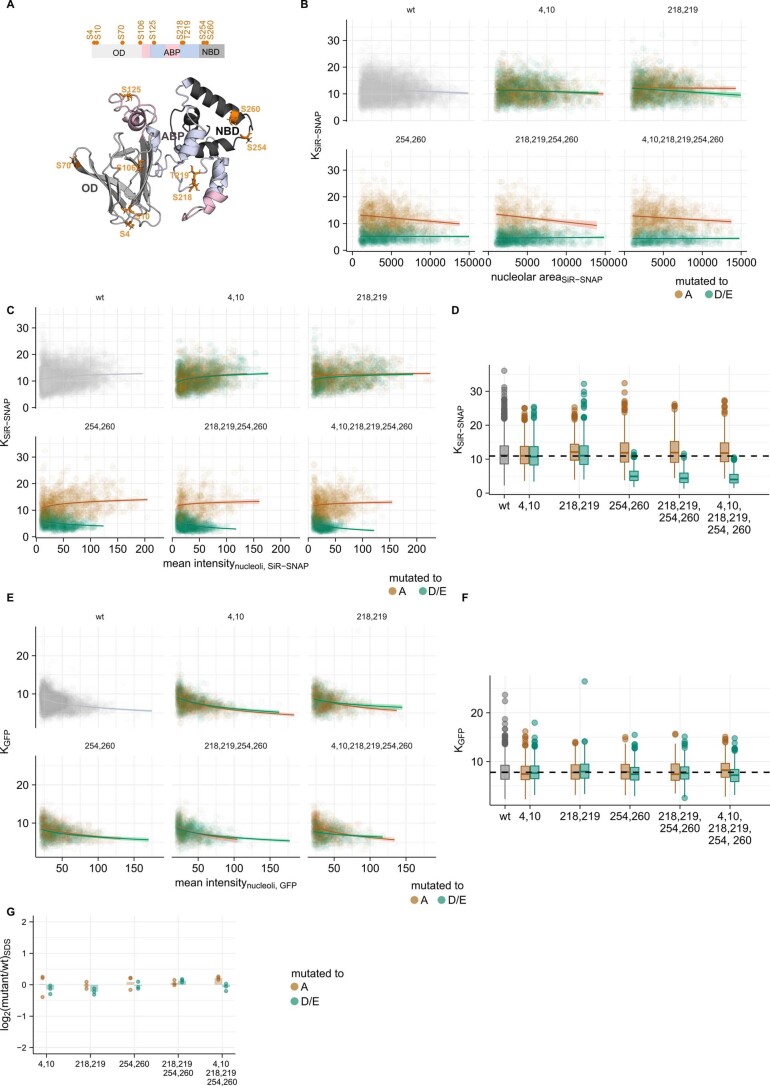
Localization of NPM1 and its phosphomutants. (**a**) Model of NPM1 with the identified phosphosites highlighted in orange. Protein domains: OD- oligomerization domain, ABP- acidic basic patch and NBD- nucleic acid binding domain. (**b**) Scatter plot of calculated partition coefficient (K) from SiR-SNAP channel (y-axis) and size of individual (segmented-) nucleoli (x-axis) from at least three independent trials per construct. (**c**) Scatter plot of calculated partition coefficient from SiR-SNAP channel (K_SiR-SNAP_, y-axis) and mean intensity of SiR-SNAP in nucleoli (proxy for the expression level of SNAP-tagged protein, x-axis) for individual (segmented-) nucleoli from at least three independent trials per construct. (**d**) Box plot showing the distribution of K_SiR-SNAP_ (partition coefficient calculated from SiR-SNAP channels for NPM1 wildtype (wt) and phosphomutants. The box plots display the median and IQR, with the upper whiskers extending to the largest value ≤1.5 × IQR from 75th percentile and the lower whiskers extending to smallest values ≤1.5 × IQR from 25th percentile. (**e**) Scatter plot of calculated partition coefficient from GFP channel (K_GFP_, y-axis) and mean intensity of GFP in nucleoli (proxy for the expression level of GFP-tagged wt-NPM1, x-axis) for individual (segmented-) nucleoli from at least three independent trials per construct. (**f**) Box plot showing the distribution of K_GFP_ (partition coefficient calculated from GFP channels for NPM1 wildtype (wt) expressed as marker of nucleoli in all experiments. The box plots display the median and IQR, with the upper whiskers extending to the largest value ≤1.5 × IQR from 75th percentile and the lower whiskers extending to smallest values ≤1.5 × IQR from 25th percentile. (**g**) Barplot representing the relative protein abundance (in log_2_ scale, y-axis) of heterologously expressed SNAP-tagged phosphomutants (x-axis) of NPM1 compared to the wild type (wt) from three biological replicates. Source data

Next, we focused on the impact of phosphorylation on the nucleolar association of NPM1, a highly abundant and essential protein that forms the granular component of the nucleolus^32^. NPM1 is a pentameric protein with distinct domain organization^33^ (Extended Data Fig. 7a). In our data, multiple phosphopeptides spanning eight sites (S4, S10, S70, S106, S125, S218|T219, S254 and S260) exhibited higher solubility than the unmodified protein (Fig. 5a), but, in particular, phosphorylation of S106, S218|T219, S254 and S260 exhibited the strongest effects that passed the significance threshold. The phosphopeptides spanning the S218|T219 site had a localization probability of 40% for S218 and 60% for T219 (Supplementary Data 3). The ability to localize phosphosites on a peptide with high confidence depends on unambiguous identification of fragment ions surrounding the phosphosite. Due to the ambiguity in the assignment, both sites were included in the subsequent analysis. Most of these sites are located in the positively charged predicted disordered segment of NPM1 (Fig. 5b), except for S70 and S260, which are located within the oligomerization and nucleic acid-binding domains, respectively (Extended Data Fig. 7a).

To assess the impact of phosphorylation on the nucleolar localization of NPM1, we designed five sets of phosphodeficient (S to A, T to A) and phosphomimetic (S to D, T to E) mutants of NPM1, which covered six phosphorylation sites. The first three mutants had two point mutations (S4 and S10, S218 and T219, S254 and S260). The second set of mutants carried four point mutations (S218, T219, S254 and S260) and six point mutations (S4, S10, S218, T219, S254 and S260), simulating the acquisition of additional phosphorylations (Fig. 5c). The phosphomutants and WT NPM1 were transiently overexpressed as SNAP-tagged fusion proteins in a HeLa cell line expressing GFP-tagged NPM1 (ref. ^34^) and imaged using confocal microscopy after labeling with a cell-permeable SNAP-tag substrate, 647-SiR. While the WT and all phosphodeficient mutants of NPM1 localized to the nucleolus, all phosphomimetic versions containing the S254 and S260 sites showed increased nucleoplasmic signal (Fig. 5d). To quantify the extent of nucleoplasmic localization, we measured the intensities of SiR-SNAP in nucleoli and the nucleoplasm and calculated their ratio as the representative of the partition coefficient *K* (Fig. 5e). The relative changes in *K* (normalized to WT values) were comparable between deficient and phosphomimetic versions of NPM1 carrying mutations at residues 4 and 10 as well as 218 and 219. However, all mutant proteins carrying phosphomimetic mutations at 254 and 260 showed lower *K* values than their deficient version (Fig. 5f, Extended Data Fig. 7b–f and Supplementary Data 4).

We further used proteomics to measure the solubility of NPM1 and its phosphomutants. The protein expression levels of the heterologously expressed NPM1 constructs were comparable (Extended Data Fig. 7d). However, the solubility (measured using SNAP-tag as a proxy to infer on different variants) of the phosphomimetic mutants of NPM1 that exhibited lower partitioning into the nucleolus was higher than that of their corresponding deficient versions (Fig. 5f).

In summary, phosphorylation at S254 and S260 prevents NPM1 from localizing to the nucleolus, keeping it in the nucleoplasm. Additional phosphorylation at S218, T219, S4 and S10 further increases the proportion of NPM1 in the nucleoplasm.

### Phosphorylation of NPM1 affects its molecular interactions

**Fig. 6:**
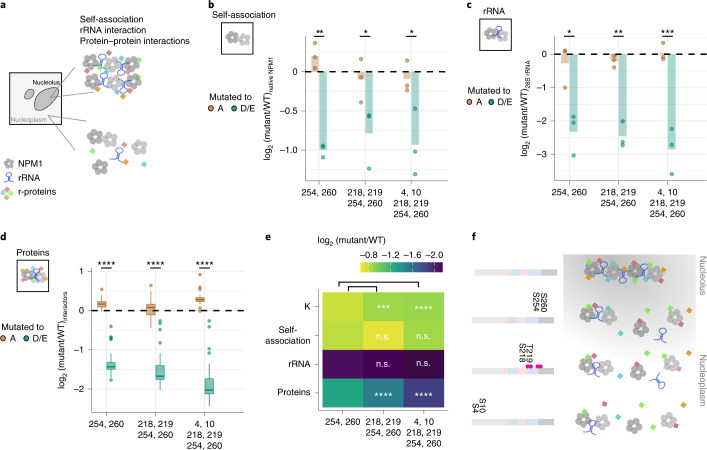
Phosphorylation impairs both homotypic and heterotypic interactions of NPM1. **a**, Schematic representation of the molecular interactions of NPM1 within the nucleolus and the nucleoplasm. **b**, Comparison of the self-association property (in log_2_ scale, *y* axis) of the indicated SNAP-tagged phosphomutants of NPM1 (*x* axis) normalized to that of SNAP-tagged NPM1 WT. This was measured by assessing the amount of HeLa cell (native) NPM1 associated with heterologously overexpressed SNAP-tagged NPM1 variants following immunoprecipitation (IP) with SNAP-tag as bait. Points represent data from three independent trials and the bar represents the mean value. Significance for each phosphomutant pair was calculated using Student’s *t*-test (two sided) and is represented by *P < 0.05, **P < 0.01 and ***P < 0.001. **c**, Comparison of the amount of 28S rRNA (in log_2_ scale, *y* axis) associated with the indicated SNAP-tagged phosphomutants of NPM1 (*x* axis) normalized to that of SNAP-tagged NPM1 WT following IP with SNAP-tag as bait. Points represent data from three independent trial and the bar represents the mean values. Significance for each phosphomutant pair was calculated using Student’s *t*-test (two sided) and is represented by *P < 0.05, **P < 0.01 and ***P < 0.001. **d**, Comparison of the relative amounts of NPM1-interacting proteins (in log_2_ scale, *y* axis; 44 proteins were classified as NPM1 interactors, including both ribosomal and non-ribosomal proteins) associated with the indicated SNAP-tagged phosphomutants of NPM1 (*x* axis) in comparison to those associated with SNAP-tagged NPM1 WT following IP with SNAP-tag as bait. Significance for each phosphomutant pair was calculated using Student’s *t*-test and is represented by *P < 0.05, **P < 0.01, ***P < 0.001 and ****P < 0.0001. The box plots display the median and the IQR, with the upper whiskers extending to the largest value ≤1.5 × IQR from the 75th percentile and the lower whiskers extending to the smallest values ≤1.5 × IQR from the 25th percentile. **e**, Heatmap showing normalized relative effect sizes of partition coefficient values (*K*) and amount of native NPM1 (reflects NPM1 self-association), rRNA and protein interactors of NPM1 associated with the indicated phosphomimetic mutants (*x* axis). Significance levels were obtained with Student’s *t*-test (two sided) and are represented by *P < 0.05, **P < 0.01, ***P < 0.001 and ****P < 0.0001. **f**, Schematic representation of the working model of the impact of phosphorylation on NPM1’s propensity to form a biomolecular condensate. Pink dots represent phosphosites, gray circles indicate NPM1, diamond shapes represent r-proteins, and the stem-loop schematic represents rRNA. Source data

**Extended Data Fig. 8:**
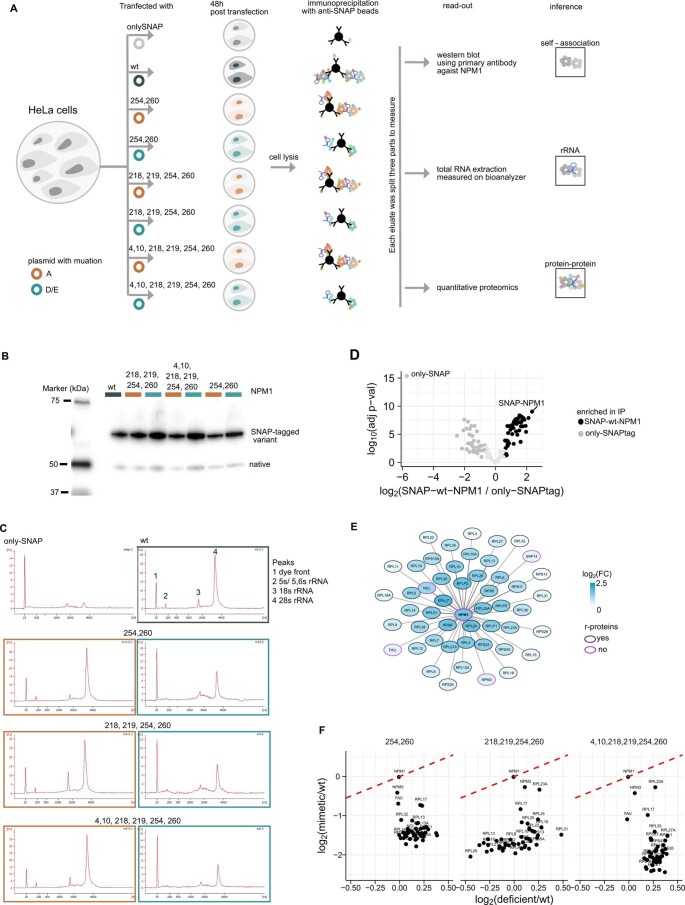
Phosphoregulation of NPM1 interactions. (**a**) Schematic representation of the experimental set-up to capture the interaction of NPM1. (**b**) Western blot image of the eluate of the IP of different variants of NPM1 using primary antibody against NPM1. Two bands corresponding to heterologous expression (SNAP-tagged, high molecular weight) and native protein (low molecular weight) are observed. Higher amounts of SNAP-tagged phosphomimetic mutants are observed due to higher accessibility (due to higher solubility) to antibody based pull-down. (**c**) RNA elution profiles from Bioanalyzer showing the fluorescence intensity (in arbitrary units, y-axis) along the elution time (in seconds, x-axis) of NPM1 and its phosphomutants. (**d**) Differential analysis of proteins associated with SNAP-tagged wild type NPM1 compared to only-SNAP tag. Proteins represented in black (solid circle) are (at least 2-fold higher with an FDR < 0.1, *limma* analysis, p-value corrected with Benjamini-Hochberg procedure) defined as the specific protein-interactors of NPM1. (**e**) Protein-protein interactions of NPM1 represented in a network visualization. Each node represents ribosomal proteins (black outline) and non-ribosomal proteins (pink outline) specifically interacting with NPM1. (**f**) Scatter plot comparing the median fold changes (from n = 3 trials) of the amount of the specific protein interactors of NPM1 associated with phosphodeficient and mimetic versions of NPM1. Source data

To elucidate the mechanism of how phosphorylation affects partitioning of NPM1 into the nucleolus, we investigated the impact of NPM1 phosphorylation on its self-association (homotypic) and heterotypic interactions with rRNA and ribosomal proteins (r-proteins), which are known to impact its condensation^11,33^ (Fig. 6a). As nucleoli remained intact after lysis (Fig. 1e and Extended Data Fig. 2a), we assessed the impact of phosphorylation on these key molecular interactions in a lysate setting by preparing cellular extracts from HeLa cells transiently overexpressing SNAP-tagged WT and mutants of NPM1 (which showed lower partitioning to the nucleolus) and quantitatively assessed the amount of native NPM1, rRNA and r-proteins associated following an affinity-based pulldown assay using SNAP-tag as bait (Extended Data Fig. 8a).

The amount of native NPM1 (from HeLa cells) bound to SNAP-tagged WT or mutant NPM1 was assessed using western blot (Extended Data Fig. 8b). All phosphomimetic mutants pulled down lower amounts of native NPM1 than their respective deficient versions (Fig. 6b). However, no significant difference between different phosphomimetic mutants of NPM1 was observed. This suggested that phosphorylation of sites S254 and S260 reduced the self-association property of NPM1, but additional phosphorylation events did not result in further reduction in NPM1–NPM1 interaction.

Next, the amount of rRNA bound to different NPM1 constructs was evaluated by extracting and analyzing the total RNA from the pulldown eluate on a bioanalyzer. WT NPM1 was predominantly associated with 28S rRNA along with small amounts of 18S, 5S and 5.6S rRNA (Extended Data Fig. 8c). The relative amount of 28S rRNA associated with the phosphomimetic mutants was lower (Fig. 6c) than that of the respective phosphodeficient mutants, suggesting that phosphorylation of S254 and S260 reduces rRNA–NPM1 interaction.

Finally, to obtain insights into the impact of NPM1 phosphorylation on its protein–protein interactions, we identified and quantified the proteins co-purifying with NPM1 (and its mutants) using quantitative proteomics. We first mapped the protein interactors of WT NPM1 by comparing the proteins that were differentially abundant in cells expressing SNAP-tagged WT NPM1 compared to SNAP-tag alone (FC > 2, adjusted *P* value < 0.1; Extended Data Fig. 8d and Supplementary Data 6). Similar to previous studies^35^, many r-proteins along with a small number of non-ribosomal proteins (including nucleolin) were found to interact with NPM1 (Extended Data Fig. 8e), which are termed ‘NPM1 interactors’. Next, we quantified the relative amounts of these protein interactors associated with NPM1 mutants compared to those associated with WT NPM1. All phosphomimetic mutants interacted with lower amounts of NPM1 interactors than their respective deficient versions (Fig. 6d and Extended Data Fig. 8f). However, NPM1 mutants carrying four (218, 219, 254 and 260) and six (4, 10, 218, 219, 254 and 260) mutations further reduced NPM1 interactors compared to the variant containing two mutations (254 and 260) (Fig. 6e). This suggested that phosphorylation of S254 and S260 reduces r-protein–NPM1 interaction and additional phosphorylation of S218, T219, S4 and S10 further reduces the ability of NPM1 to associate with these proteins.

In summary, our results suggest that phosphorylation of S254 and S260 in the vicinity of the nucleic acid-binding domain is pivotal for dissociating NPM1 from the nucleolus by modulating all three modes of molecular interactions: self-association, protein–RNA and protein–protein. The additional phosphorylation of S218, T219, S4 and S10 increases the propensity of the protein to localize to the nucleoplasm, primarily through reduction of NPM1–protein interactions (Fig. 6e,f).

## Discussion

In this work, we systematically classified proteins based on their solubility and identified phosphorylation sites specific to distinct subpools of proteins under steady-state conditions. Several proteins that are annotated to be part of biomolecular condensates and proteins that form structural polymers maintained stable insoluble subpopulations under lysate conditions. These proteins are likely to be the core interactors or ‘scaffold proteins’ (ref. ^1^) of the condensates, and the weak interactors or ‘client proteins’ (refs. ^1,3^) are likely lost due to cell lysis-mediated dilution. Nearly a third of the insoluble proteome required cellular RNA to retain its insoluble subpool, in agreement with RNA–protein interactions playing a central role in the assembly of condensates^36^. High-affinity interactions of proteins and RNA known to enable protein condensation^37^ are likely to remain intact in cell lysates, while weak non-specific interactions of RNA with proteins, reported to decrease protein condensation^38^, are expected to be lost due to cell lysis-mediated dilution. Hence, digestion of RNA in cell lysates is expected to affect biomolecular condensate-associated proteins, and indeed we observe a global increase in protein solubility due to disruption of high-affinity RNA–protein interactions. Distinguishing distinct subpools of proteins allows the characterization of subpopulation-specific signatures that drive and stabilize molecular interactions of condensates, such as the impact of phosphorylation.

The importance of phosphorylation in the regulation of biomolecular condensates has been demonstrated through the central role of casein kinase 2 (CK2) and dual-specificity tyrosine kinase 3 (DYRK3) in the disassembly of stress granules^39^ and nuclear speckles during mitosis^40^. Recent studies have shown a global impact of phosphorylation on condensation of RNA-binding proteins^41^. However, the site-specific information necessary to understand mechanisms of phosphoregulation of protein condensation is unknown. Our dataset addresses this knowledge gap and provides the foundation for systematically uncovering the mechanisms of phosphorylation-mediated regulation of biomolecular condensates.

Most phosphosites occur within the intrinsically disordered segments of proteins, which sample an ensemble of conformations. Protein phosphorylation introduces two negative charges that can alter chemical, steric and electrostatic properties of amino acid side chains, which can induce a range of structural alterations^42,43^. The biases in sequence properties observed for differentially soluble phosphosites allows inference into the likely consequence of phosphorylation on the conformational characteristics of the local disordered regions. Phosphorylation of disordered segments with mild hydrophobicity and a low proportion of charged residues (as in the vicinity of phosphosites enriched in the soluble subpool) is likely to favor transition from compact conformations to expanded coils (Fig. 3f)^28,44^. Conversely, phosphorylation of positively charged disordered segments as in the surroundings of phosphosites enriched in the insoluble protein pools can potentially transform these segments into polyampholytes, which is likely to increase the valency of intrachain and interchain interactions (Fig. 3f)^45,46^. The phosphorylation sites suggested to impact RNA-binding properties have a high proportion of aromatic amino acids in their vicinity. Many prion-like domains containing RNA-binding proteins have been reported to undergo phase transition through π–π and cation–π interactions between the side chains of arginine and tyrosine residues^47^, which could be disrupted upon phosphorylation.

For HNRNPA1, we observe that multisite phosphorylation of its C-terminal disordered segment impacts its condensation. Acquisition of 12 negative charges due to the phosphorylation of six residues will increase the proportion of charged residues from 0.129 to 0.323 of the local disordered segment (last 31 amino acids of the protein), with a net negative charge (−0.26). This increase in charge density is likely to promote disorder^45,48^ of the C-terminal tail, which can affect its RNA binding and condensation. For NPM1, we observe phosphorylation affecting the continuum of molecular interactions necessary for condensation. The key phosphosites S254 and S260 likely drive a conformational switch by promoting order-to-disorder transition, which has a direct impact on nucleic acid binding. This phosphorylation switch can then prime the protein for further phosphorylation, which moves the protein from the nucleolus to the nucleoplasm by primarily impacting its protein–protein interactions (Fig. 5f).

Our approach can be combined with any PTM enrichment to assign the PTM state of condensate-bound and soluble pools of proteins. This will enable delineation of the crosstalk between distinct PTMs. Multiple modifications of the same amino acid can compete with each other, for example, *O*-linked-*N*-acetylglucosaminylation of S or T competes with phosphorylation^49^. Modifications on different amino acids can build cooperativity or antagonism of interactions, for example, acetylation of tau prevents its phosphorylation and increases its solubility^50^. Subsequent investigations using targeted inhibition or knockout of writers (for example, kinases) will allow the assignment of enzymes responsible for the modifications of proteins and thus provide tools to modulate their solubility behavior.

In terms of limitations, our approach is expected to map both driver and passenger phosphorylation events. For example, in the case of NPM1, phosphorylation of S254 and S260 are driver events necessary to prevent localization to the nucleolus. Phosphorylation of S4, S10, S218 and T219 are likely passenger events that independently have a minimal effect on NPM1 solubility but are enriched in the soluble pool. Furthermore, in cases in which multiple proximate sites can be phosphorylated, assigning the exact location of phosphorylation becomes challenging due to technical difficulties of mapping multiphosphorylated peptides by MS^31^.

In summary, we present a system-wide approach, complementary and orthogonal to microscopy-based experiments, for the study of biomolecular condensates and systematically characterize the RNA dependency and phosphorylation signatures of protein condensates. Our study is a step toward understanding the widespread impact of phosphoregulation of biomolecular condensates.

## Methods

### Cell culture

All cell lines used in this study were verified to be negative for mycoplasma contamination.

#### For the proteomic assay

HeLa Kyoto cells (a kind gift from the Ellenberg group, EMBL) were cultured in DMEM (Sigma-Aldrich, D5648) containing 1 mg ml^−1^ glucose, 10% (vol/vol) FBS (Gibco, 10270) and 1 mM l-glutamine (Gibco, 25030081) at 37 °C with 5% CO_2_. HeLa cells (0.5 million) were seeded in 150-mm dishes and grown for 2 d. The cells were washed with ice-cold PBS (2.67 mM KCl, 1.5 mM KH_2_PO_4_, 137 mM NaCl and 8.1 mM NaH_2_PO_4_, pH 7.4) and collected by scraping. The cells were pelleted by centrifugation at 300*g* for 3 min. The cell pellets were flash frozen in liquid N_2_ and stored at −80 °C.

#### For imaging

HeLa Kyoto cells were cultured in DMEM containing 10% (vol/vol) FBS, 1% (vol/vol) penicillin–streptomycin (Sigma-Aldrich), 1% (vol/vol) GlutaMAX (Gibco, 35050038) and selected antibiotics as appropriate for the expression constructs: G418 (1 mg ml^−1^, Invitrogen). All NPM1 mutant proteins were visualized through transient transfection of a HeLa cell line overexpressing WT GFP-tagged NPM1 from a BAC^34^ with constructs encoding SNAP-fused proteins on a Lab-Tek chambered coverglass (Thermo Fisher Scientific). Live cell imaging was performed in DMEM containing 10% (vol/vol) FBS, 1% penicillin–streptomycin and 1% (vol/vol) GlutaMAX (Gibco, 35050038) without riboflavin or phenol red to reduce autofluorescence.

### Solubility profiling of cellular lysates

Lysis buffer was composed of PBS containing 1 U ml^−1^ RNase inhibitors (RNasin Plus, N2615), cOmplete protease inhibitors (Roche), PhosSTOP (phosphatase inhibitors, Roche), 1.5 mM MgCl_2_, 2 mM NaF, 2 mM Na_3_VO_4_, 2 mM Na_4_P_2_O_7_ and 10 mM CH_3_CH_2_CH_2_COONa. Frozen HeLa cell pellets were thawed on ice and resuspended in a volume of lysis buffer equal to twice the volume of the pellet. This homogeneous cell suspension was subjected to mechanical disruption by three freeze–thaw cycles (freezing in liquid nitrogen and thawing at 25 °C). The protein concentration in the lysate was determined using the Rapid Gold BCA assay (Thermo Fisher Scientific, A53225). The lysate was diluted to 3.5 mg ml^−1^ and split into three aliquots of 100 µl each. The first aliquot represented the RNA-preserved lysate, the second was an RNA-digested lysate to which 2 µl RNase cocktail (Thermo Fisher Scientific, AM2286) was added, and the last portion represented the total proteome to which 1 µl of Benzonase (Sigma, E1014) was added. All three aliquots were incubated at 4 °C on a shaking platform (500 r.p.m.) for 30 min. The RNA-preserved and RNA-digested samples were solubilized with NP-40 (final concentration, 0.8%), while the total proteome aliquot was solubilized with SDS (final concentration, 1%). The RNA-preserved and RNA-digested lysates were spun at 100,000*g* and 4 °C for 20 min. The supernatants containing the soluble pool of proteins were obtained, and the pellet containing the insoluble pool of proteins was washed with 100 µl lysis buffer (containing 0.8% NP-40) twice and finally solubilized in 100 µl lysis buffer containing 1% SDS and 0.25 U ml^−1^ Benzonase. The insoluble protein pools and total proteome aliquots were incubated at room temperature for 15 min followed by a 5-min incubation at 90 °C. The protein concentration of the total proteome was assessed with the Rapid Gold BCA assay (Thermo Fisher Scientific, A53225), and the volume of lysate containing 125 µg total protein was determined. Equal volumes of ‘soluble’ supernatants and ‘insoluble’ pellets were used for multiplexing for MS measurements. Three independent trials using cell pellets generated from different passages of HeLa cells were performed.

### Plasmids and transfection

The open reading frames of *FBL*, *NOP56*, *COIL* and *PRPF6* were obtained from GenScript. These sequences were cloned into the pcDNA3.1 vector backbone to express them as eGFP (C-terminus) fusion proteins. Sequences for GFP-tagged (N-terminal) versions of HNRNPA1 and its phosphomutants were cloned into the pIRES backbone. Sequences for SNAP-tagged (N-terminal) versions of NPM1 and its phosphomutants were cloned into the pIRES backbone. Transient transfections were performed with PEI transfection reagent (1 mg ml^−1^ stock, Polysciences) 48 h before imaging or solubility profiling. Plasmid amounts were optimized for each assay and varied between 0.25 µg and 2 µg DNA per 3 µl transfection reagent and 100 µl Opti-MEM.

### Live cell microscopy

SNAP–NPM1 constructs were labeled with SNAP-Cell 647-SiR (NEB, S9102S) following the supplier’s instructions. All confocal microscopy images were acquired on a customized confocal Zeiss LSM780 microscope, using a ×40 or ×63, 1.4-NA oil-immersion objective in DIC mode with a Plan-Apochromat objective (Zeiss), operated with ZEN 2011 software. During acquisition, an in-house-built incubator provided a humidified atmosphere and a constant temperature of 37 °C with 5% CO_2_.

### Permeabilization and fixation of cells for microscopy

Cells transfected to express GFP-tagged FBL, NOP56, COIL, PRPF6, HNRNPA1 and SNAP-tagged NPM1 (after staining) were permeabilized with lysis buffer (PBS containing 1 U ml^−1^ RNasin, cOmplete protease inhibitors, PhosSTOP phosphatase inhibitors, 1.5 mM MgCl_2_ and 0.8% NP-40) with or without RNase cocktail (1 µl per 100 µl lysis buffer) for 15 min at room temperature. Following permeabilization, cells were fixed with formaldehyde (4% final concentration) for 10 min at room temperature. Subsequently, cells were washed twice with PBS, and images were acquired using a Zeiss LSM780 confocal microscope with a ×63, 1.4-NA oil-immersion objective in DIC mode with a Plan-Apochromat objective (Zeiss), operated with ZEN 2011 software. These experiments were performed in three independent trials.

### Lysate imaging

HeLa cells transfected to express GFP-tagged FBL, NOP56, COIL, PRPF6 and the GFP-NPM1-Bac-HeLa cell line were lysed as described (For the proteomic assay) for solubility profiling. The cell lysate was seeded in an eight-well imaging dish and placed on ice for 15 min. Following the incubation, the imaging dish was moved to room temperature for 10 min (to best suit imaging acquisition). Images were acquired using a Zeiss LSM780 confocal microscope with a ×63, 1.4-NA oil-immersion objective in DIC mode with a Plan-Apochromat objective (Zeiss), operated with ZEN 2011 software.

### Solubility profiling of HNRNPA1 and NPM1 phosphomutants

HeLa cells (50,000 cells per well in a 24-well plate) were transfected with (0.9 µg DNA with 2 µl PEI transfection reagent in 100 µl Opti-MEM) plasmids encoding different phosphomutants of NPM1 and HNRNPA1 and WT protein. After transfection (~48 h), the cells were lysed with 100 µl lysis buffer (PBS containing 1 U ml^−1^ RNasin, cOmplete protease inhibitors, PhosSTOP phosphatase inhibitors, 1.5 mM MgCl_2_ and 0.8% NP-40). The lysate was split into two equal aliquots. One aliquot was centrifuged at 100,000*g* for 20 min at 4 °C, and the supernatant was retrieved. The second aliquot was treated with 1 µl Benzonase (50 U µl^−1^) on ice for 20 min and further solubilized with SDS (final concentration, 1%). Total protein concentration in the lysate was measured with the Rapid Gold BCA assay (Thermo Fisher Scientific, A53225), and the volume of the lysate representing 5 µg total protein was determined. The same volume of ‘soluble’ supernatant and the total protein fraction was used for multiplexing for MS measurements. At least three independent biological replicates were performed.

### Immunoprecipitation of NPM1 phosphomutants

HeLa cells transfected with plasmids encoding SNAP tag alone, SNAP-tagged versions of WT and phosphomutants of NPM1 (~48 h) were lysed (with lysis buffer, 10 mM Tris-HCl, pH 7.5, 150 mM NaCl, 0.5 U ml^−1^ RNase inhibitors, cOmplete protease inhibitors, PhosSTOP, 0.5 mM EDTA and 0.8% NP-40) and centrifuged at 1,000*g* for 5 min at 4 °C to clear the lysate of cell debris. The supernatant was transferred to a new tube, and an equal volume of dilution buffer (10 mM Tris-HCl, pH 7.5, 150 mM NaCl, 0.5 U ml^−1^ RNasin, cOmplete protease inhibitors, PhosSTOP and 0.5 mM EDTA) was added. An aliquot (30 µl) of this lysate was stored for analyzing the variation in input, and the remaining lysate was used for IP. A SNAP/CLIP-tag-Trap-Agarose (ChromoTek, wta-10) bead slurry was washed with dilution buffer and incubated with lysate in a spin column for 1 h on a rotating platform (10 r.p.m.) at 4 °C. Following the incubation, the flow through was collected by centrifugation (1,000*g*, 30 s at 4 °C). The beads were washed three times with wash buffer (10 mM Tris-HCl, pH 7.5, 150 mM NaCl, 0.2 U ml^−1^ RNasin, cOmplete protease inhibitors, PhosSTOP, 0.5 mM EDTA and 0.05% NP-40). Finally, the protein–RNA complex was eluted by incubating the beads with 70 µl elution buffer (10 mM Tris-HCl, pH 7.5, 150 mM NaCl, cOmplete protease inhibitors, PhosSTOP, 0.5 mM EDTA, 1% SDS) for 15 min at room temperature on a rocking platform (700 r.p.m.). The eluate was split into 3 × 20-µl aliquots (to be analyzed by western blot, multiplexed quantitative MS and the bioanalyzer after extracting RNA) to measure protein–protein and protein–RNA interactions of NPM1. It is noteworthy that, although all variants of NPM1 overexpressed to similar levels, phosphomimetic mutants that exhibited higher solubility had higher accessibility for antibody-based pulldown, and hence data-correction steps for pulldown efficiency have been included as described in Data analysis. Data interpretation was carried out with data from at least three independent IP experiments.

#### Western blotting

One aliquot of the eluate was reduced (37 °C, 1 h) and heat denatured (95 °C, 5 min) after addition of an equal volume of 2× sample buffer (150 mM Tris-HCl, 2% SDS, 30% glycerol, 0.04% bromophenol blue, 20 mM Tris(2-carboxyethyl)phosphine (TCEP)). The samples were separated by SDS–PAGE using 4–15% Mini-PROTEAN polyacrylamide gels (Bio-Rad) and 1× Laemmli buffer at constant voltage (100 V) for 75–90 min. The proteins were transferred onto a 0.2-µm PVDF membrane using semi-dry transfer (Trans-Blot Turbo Transfer System, Bio-Rad) and 1× Trans-Blot transfer buffer at 1.3 A and 25 V for 10 min. The membrane was blocked with 5% non-fat milk prepared in PBS containing 0.1% Tween (blocking buffer). Mouse monoclonal IgG against NPM1 (sc-32256, Santa Cruz Biotechnology, clone FC-8791) was diluted in blocking buffer (1:2,000 dilution) and incubated with the membrane overnight at 4 °C. The membrane was washed three times and incubated with goat anti-mouse IgG–HRP (sc-2005, Santa Cruz Biotechnology, 1:5,000 dilution) for 1 h at room temperature. The membranes were washed and developed using an enhanced chemiluminescence kit (Bio-Rad) using the manufacturer’s instructions. Two bands (one from native HeLa cell NPM1 at ~45 kDa and one from heterologous expression of SNAP–NPM1 at ~65 kDa) were detected and quantified using ImageJ (version 1.53e).

#### RNA isolation and analysis on the bioanalyzer

rRNA associated with NPM1 and its phosphomutants was assessed by extracting total RNA from the second aliquot of the IP eluate using the RNeasy Mini kit (74004, Qiagen) following instructions from the manufacturer. The isolated RNA was run on a 2100 Bioanalyzer instrument (Agilent) programmed using 2100 Expert software after preparing the samples on a chip using the Agilent RNA 6000 Pico kit according to the manufacturer’s instructions. Area under the curve corresponding to 28S rRNA was obtained from 2100 Expert software.

#### Multiplexed quantitative proteomics

Proteins in the third aliquot of the IP eluate were incubated at 37 °C for 1 h following addition of TCEP (final concentration, 10 mM). The samples were digested and labeled to be analyzed on a mass spectrometer as described below.

### Mass spectrometry sample preparation

#### Protein digestion and labeling

Three biological replicates of the solubility-profiling experiments were multiplexed as a single MS run. Different lysates (as described above) were diluted with an equal volume of sonication buffer (1% sodium deoxycholate, 5 mM TCEP, 30 mM chloroacetamide, 1 mM MgCl_2_, 10 U µl^−1^ Benzonase) and sonicated in a Bioruptor for 15 cycles (30 s on, 30 s off) to remove nucleic acids.

A modified SP3 protocol was used to perform protein digestion^51^. Briefly, the protein samples were incubated with a paramagnetic bead slurry (10 µg Sera-Mag SpeedBeads per 5 µg protein, Thermo Fisher Scientific, 4515‐2105‐050250, 6515‐2105‐050250) in ethanol (at 70%). This mixture was incubated for 15 min at room temperature with shaking and subsequently was washed four times with 70% ethanol. Proteins precipitated on beads were alkylated, reduced and digested overnight using 100 µl digest solution (100 mM HEPES, pH 8, containing 5 mM chloroacetamide, 1.7 mM TCEP, 1 µg µl^−1^ trypsin, 1 µg µl^−1^ LysC). The resulting peptides were eluted from the beads, dried under vacuum and reconstituted in 100 µl water. Peptide labeling was performed with TMT 16-plex reagents (dissolved in 20 µl acetonitrile) at a 1:5 (peptide:TMT) weight ratio for 1 h at room temperature. This reaction was quenched with 5 µl 5% hydroxylamine and was pooled together for a single MS experiment. The pooled sample was desalted with solid-phase extraction after acidification with trifluoroacetic acid (TFA, final concentration of 1%). The sample was loaded onto a Waters tC18 Sep-Pak 50-mg column, washed twice with 1 ml 0.1% TFA and finally eluted with 400 µl 50% acetonitrile containing 0.1% TFA. The labeled and desalted peptides were split into two aliquots containing 20 µl (5% of the labeled peptides) and 380 µl eluate. Both aliquots were dried by lyophilization.

### Phosphopeptide enrichment

The enrichment of phosphopeptides was performed using Fe^3+^-immobilized metal ion affinity chromatography as described in ref. ^13^. Briefly, the enrichment steps were performed using a ProPac IMAC-10 column (Thermo Fisher Scientific, 4 × 50 mm) on an UltiMate 3000 HPLC liquid chromatography system (Thermo Fisher Scientific). The lyophilized peptides were dissolved in buffer A (70% acetonitrile, 0.07% TFA) and loaded on the column at 400 µl min^−1^. The loaded peptides were washed for 6 min at 1 ml min^−1^ with buffer A. Finally, isocratic elution of the phosphopeptides was performed using 50% buffer B (0.3% ammonia) for 2 min at 0.5 ml min^−1^. Both unbound and phosphopeptide fractions were collected and lyophilized.

### High-pH fractionation

To acquire the unmodified protein data, the aliquot containing 5% of labeled peptides was dissolved in 15 µl 20 mM ammonium formate, pH 10 and fractionated using C18-based reversed-phase chromatography with a Phenomenex Gemini 3-µm C18 110-Å 100-mm × 1-mm column. Mobile phase was buffer A (20 mM ammonium formate, pH 10) and buffer B (acetonitrile). The peptides were resolved over an 85-min gradient run at 0.1 ml min^−1^ in the following gradient: 0% B for 0–2 min, linear increase from 0% to 35% B in 2–60 min and 35% to 85% B in 60–62 min, hold at 85% B until 68 min, linear decrease to 0% in 68–70 min and finally equilibration of the system at 0% B until 85 min. Fractions measuring 200 µl each were collected over 2–70 min, and every 12th fraction was pooled together and vacuum dried.

An in-house packed C18 microcolumn was used for fractionation of phosphopeptides. This column was prepared by inserting a C18 resin plug (Affinisep AttractSPE C18 Disks) into gel-loaded tips, which were then filled with 1 mg ReproSil-Pur C18 material (Dr. Maisch, 5 µm, 120 Å). The lyophilized phosphopeptides were resuspended in 40 µl 20 mM ammonium formate, pH 10 (buffer A) and loaded onto the microcolumn using centrifugation (loading speed, ~10 µl min^−1^). The peptides were washed twice with 10 µl buffer A and eluted using a stepwise gradient of increasing concentrations of acetonitrile in buffer A starting from 1% until 30% (increments of 2%), followed by 35% and 40%. The flow through and wash were pooled together and considered as the FT fraction, and every sixth fraction of the elution was pooled together and lyophilized.

### Liquid chromatography with tandem mass spectrometry measurement

The fractionated peptides were resuspended in 0.05% formic acid and analyzed on Q Exactive Plus or Orbitrap Fusion Lumos mass spectrometers (Thermo Fisher Scientific). Chromatographic separation was performed on the UltiMate 3000 RSLCnano system (Thermo Fisher Scientific) equipped with a trapping cartridge (precolumn; C18 PepMap 100, 5 μm, 300-μm i.d. × 5 mm, 100 Å) and an analytical column (Waters nanoEase HSS C18 T3, 75 μm × 25 cm, 1.8 μm, 100 Å). The mobile phase constituted 0.1% formic acid in LC–MS-grade water (buffer A) and 0.1% formic acid in LC–MS-grade acetonitrile (buffer B). The peptides were loaded onto the trap column (30 μl min^−1^ of 0.05% TFA in LC–MS-grade water for 3 min) and eluted using a 120-min gradient at 0.3 μl min^−1^ (2% to 30% buffer B, followed by an increase to 40% B and a final wash to 80% B for 2 min before re-equilibration to initial conditions). The outlet of the LC system was directly coupled for MS analysis using a Nanospray Flex ion source and a PicoTip Emitter (360-μm o.d. × 20-μm i.d.; 10-μm tip, New Objective). The mass spectrometer was operated in positive ion mode with a spray voltage of 2.2 kV and capillary temperature at 275 °C. Full-scan MS spectra with a mass range of 375–1,200 *m*/*z* were acquired in profile mode using a resolution of 70,000 (maximum fill time of 10 ms and a maximum automatic gain control (AGC) of 3 × 10^6^ ions). MS was run in data-dependent acquisition mode, and fragmentation was triggered for the top ten most intense peaks with charge 2–4 with a 30 seconds dynamic exclusion window (normalized collision energy was 30), and MS/MS spectra were acquired in profile mode with a resolution of 35,000 (maximum fill time of 120 ms and an AGC target of 2 × 10^5^ ions).

Phosphopeptide fractions were resuspended in a mixture of 50 mM citric acid and 1% formic acid. The sample was loaded on the trap column and subsequently separated using a linear gradient from 8% to 25% buffer B, followed by an increase to 40% buffer B in 120 min. Full scans were acquired in the Orbitrap with a scan range of 375–1,400 *m*/*z*, and precursors were sequentially isolated and fragmented with a 30-s dynamic exclusion window. MS/MS spectra were acquired in the Orbitrap at a resolution of 30,000 with an AGC target of 1 × 10^5^ charges and a maximum injection time of 110 ms.

### Protein identification and quantification

MS data were processed as described in ref. ^13^. Briefly, raw MS data were processed with isobarQuant^31^, and peptide and protein identification was performed with Mascot 2.5.1 (Matrix Science) against a database containing *Homo sapiens* UniProt FASTA files (proteome ID UP000005640, downloaded on 14 May 2016) along with known contaminants and the reverse protein sequences (search parameters: trypsin; three missed cleavages; peptide tolerance of 10 ppm; MS/MS tolerance of 0.02 Da; fixed modifications included carbamidomethyl on cysteines and TMT 10-plex or TMT 16-plex on lysine; variable modifications included acetylation of protein N termini, methionine oxidation and TMT 16-plex on peptide N termini).

Phosphopeptide raw data were processed with both isobarQuant as well as MaxQuant software (version 1.6.15)^52^ to assess the phosphorylation site-localization probabilities. Search parameters were set to trypsin digestion with a maximum of three missed cleavages, TMT 10-plex labeling, fixed carbamidomethylation of cysteines and variable oxidation of methionines, as well as variable phosphorylation of serine, threonine and tyrosine residues. Mass tolerance was set to 4.5 ppm at the MS^1^ level and 20 ppm at the MS^2^ level. A score cutoff of 40 was used for modified peptides, the false discovery rate was set to 0.01, and the minimum peptide length was set to seven residues.

### Data preprocessing

#### Unmodified proteins

The summed intensities of proteins that were identified with two or more unique peptides from all three biological replicates were selected for downstream analysis. Protein FDRs were determined using the picked approach and set to be below 0.01.

#### Phosphopeptides

The search outputs of MaxQuant and isobarQuant were merged using the peptide MS/MS scan ID. Peptide identification and localization probability information was used from MaxQuant output, while quantification parameters were obtained from isobarQuant output. Data-quality criteria were set to signal-to-interference ratio ≥0.5 and precursor-to-threshold ratio ≥4 to minimize ratio compression originating from co-isolated peptides, and phosphopeptides quantified in all three replicates were used for the subsequent analysis^13^. The phosphopeptides (fulfilling the above criteria, 7,026 peptides in this dataset) with unique modification only based on phosphorylation pattern (disregarding the variation in the presence of N-terminal TMT labeling, methionine oxidation and N-terminal acetylation) were collapsed by summing their intensities. Next, phosphopeptides with highly reproducible measurements in all three replicates (s.d. < 1) were chosen for subsequent analysis. This dataset contained 4,324 peptides with a unique phosphorylation pattern that satisfied all the above data-quality criteria.

### Data analysis

#### Differential analysis of protein solubility

All statistical analysis was performed using RStudio (version 1.2.1335 and R version 3.6.1).

##### Mapping proteins with substantial insoluble subpopulation

Data normalization to minimize technical variation was performed based on a subset of proteins that are predominantly soluble, meaning proteins that exhibit comparable signal sum intensities between NP-40 and SDS channels. This subset was defined by calculating the NP-40/SDS ratio of all proteins using the raw signal sum intensities, and proteins with this ratio between 0.8 and 1.2 were chosen. Using this subset, the calibration and transformation parameters of ‘vsn’ (ref. ^53^) were obtained and further applied to all proteins. The log_2_-transformed normalized signal sum intensities of NP-40-derived and SDS-derived proteins from three replicates of RNA-preserved and RNA-digested lysates were compared using the limma package^54^. Proteins that exhibited |log_2_ (FC)| > 0.5 and adjusted *P* value (Benjamini–Hochberg) < 0.01 were considered to maintain substantial insolubility.

##### Mapping proteins with RNase-sensitive solubility behavior

The solubility of proteins in RNA-preserved and RNA-digested lysates was computed as a ratio of vsn-normalized NP-40-derived and SDS-derived abundances from three independent replicates. The log_2_-transformed solubility values from the two lysate types were differentially analyzed using the limma package^54^. Proteins that exhibited |log_2_ (FC)| > 0.5 and adjusted *P* value (Benjamini–Hochberg) < 0.01 were considered to be significantly affected due to RNase treatment.

#### Differential analysis of phosphopeptide solubility

##### Mapping differentially soluble phosphopeptides

Due to substoichiometric phosphorylation of proteins, the total protein solubility (described above), which is a weighted average of all the proteoforms available for a gene product, was used as a proxy for the non-phosphorylated (unmodified) proteoforms. The enriched phosphopeptides represent a subset of these proteoforms that can be experimentally differentiated. The NP-40 and SDS abundance of phosphopeptides that matched the required quality criteria (described above) was normalized using vsn as described for proteins. The solubility of the phosphopeptides was computed as the ratio of normalized NP-40 and SDS intensities from the RNA-preserved lysate. The log_2_-transformed solubility values of phosphopeptides and unmodified proteins from three biological replicates were differentially analyzed using the limma package. Phosphopeptides that exhibited |log_2_ (FC)| > 0.5 and adjusted *P* value (Benjamini–Hochberg) < 0.01 were considered to be significantly changing in solubility compared to their corresponding unmodified proteins.

##### Mapping differentially RNA-bound phosphopeptides

The ‘RNA-bound’ fraction of phosphopeptides and unmodified proteins was calculated by taking the ratio of their solubility in RNA-preserved and RNA-digested lysates. The log_2_-transformed RNA-bound fractions of phosphopeptides and respective unmodified proteins were differentially analyzed using the limma package. Phosphopeptides that exhibited |log_2_ (FC)| > 0.5 and adjusted *P* value (Benjamini–Hochberg) < 0.01 were considered to significantly differ in solubility compared to their corresponding unmodified versions.

#### Mapping of protein subpopulation-specific phosphosites

To categorize phosphosites specifically enriched in a certain protein subpopulation, all identified phosphopeptides encompassing a certain phosphosite were required to exhibit similar trends in their solubility profile. If a phosphosite (found from multiple peptides) unambiguously exhibited (1) higher solubility than the unmodified protein (as described above), it was called ‘soluble’, meaning that these sites were enriched in the soluble protein subpool, (2) lower solubility than the unmodified protein, it was called ‘insoluble’, referring to its enrichment in the insoluble protein subpool, (3) a higher RNA-bound fraction than unmodified protein, it was called ‘facilitates RNA association’ and (4) a lower RNA-bound fraction than unmodified protein, it was called ‘represses RNA association’. If multiple phosphopeptides mapping onto a phosphosite exhibited different trends in solubility and/or RNA-bound fraction analysis, it was called ‘ambiguous’.

#### Mapping protein interactors of NPM1 and its phosphomutants

##### Differential analysis of SNAP- and WT SNAP–NPM1-bound proteins

The signal sum intensities of proteins identified from all constructs (from three biological replicates) were normalized using vsn. The log_2_-transformed normalized signal sum intensities of SNAP- and WT SNAP–NPM1-pulled-down proteins were compared using the limma package. Proteins that exhibited log_2_ (FC) > 1 and adjusted *P* value (Benjamini–Hochberg) < 0.01 were considered to be specific interactors of NPM1.

##### Relative abundance of NPM1 interactors associated with phosphomutants

Ratios of vsn*-*normalized intensities of phosphomutant- and WT NPM1-pulled-down proteins were computed. Variations in sample input for each mutant were adjusted using the same ratio calculated from the input of the IP. Next, all mutant values were normalized for the amount of NPM1 pulled down with each mutant (to correct for the IP efficiency of each construct). The distribution of the median of corrected FCs (mutant/WT) of NPM1-specific interactors was compared between phosphodeficient and phosphomimetic mutants using two sample *t*-tests.

#### Solubility data visualization

UniProt information of protein length, domains and known phosphorylation sites was obtained and visualized using the drawProteins R package^55^. Median solubilities (from three independent trials) of the different phosphopeptides of a protein and its unmodified version were displayed along with the schematic of the protein.

#### Physicochemical properties of proteins

The full-length sequences of proteins identified were obtained from the UniProt human reference proteome. Hydrophobicity (using the Kyte–Doolittle scale) and isoelectric points (using the EMBOSS method) of all proteins were calculated using the Peptides R package^56^. The intracellular protein concentrations of all identified and quantified proteins were calculated using the histone ‘proteomic ruler’ approach^57^. Percentages of predicted structural disorder of these proteins were obtained from the D^2^P^2^ database^58^. The statistical significance of the distribution of these parameters between proteins categorized as ‘predominantly soluble’, ‘RNase-sensitive insoluble’ and ‘RNase-insensitive insoluble’ was assessed using the Wilcoxon signed-rank test.

#### Gene ontology over-representation analysis

The over-representation analysis of GO cellular compartment terms for proteins that exhibited substantial insolubility in RNA-preserved and RNA-digested lysates (related to Fig. 1c) was performed using clusterProfiler^59^ using all identified proteins from the dataset as the background. GO terms with *P* value < 0.05, Benjamini–Hochberg procedure for multiple-testing adjustment and *q* value < 0.05 were considered to be significantly over-represented among differential soluble proteins. A similar analysis was performed for phosphopeptides that exhibited differential solubility compared to their unmodified proteins (related to Fig. 3e). Proteins to which individual phosphopeptides mapped were considered as the phosphoprotein. All proteins for which a phosphopeptide was identified were used as the background.

#### Protein domain over-representation analysis

Protein domain-enrichment analysis was performed using the Pfam protein family database via the DAVID platform (version 6.8)^60^ for proteins that exhibited differential solubility in RNA-preserved and RNA-digested cellular lysates. Pfam terms with an adjusted *P* value (Benjamini–Hochberg) < 0.05 were considered to be significantly over-represented.

#### Kinase over-representation analysis

Analysis of enrichment of substrates of kinases was performed using known kinase–substrate relationships from a comprehensive resource of phosphosite annotations of direct substrates of kinases obtained from six databases: PhosphoSitePlus, SIGNOR, HPRD, NCI-PID, Reactome and the BEL Large Corpus and using three text-mining tools, REACH, Sparser and RLIMS-P^20^. Over-representation analysis was performed for each subgroup of phosphosites (soluble, insoluble or not changing) via a hypergeometric test using the ‘enricher’ function part of the clusterProfiler^59^ package in R.

#### Phosphosite activities across cell line perturbations

Activities of phosphosites were estimated in different subgroups (soluble, insoluble or not changing) from phosphoproteomic measurements of cell line perturbations across a range of biological conditions including drug or inhibitor treatments and cell cycle states from a large resource of previously published phosphoproteomic datasets^19^. Activities of different subgroups of phosphosites were inferred as −log_10_ (*P* value) of *Z*-tests from the comparison of FCs in phosphosite measurements against the overall distribution of FCs across all the phosphosites detected in this study and mapped to the phosphoproteomic resource. This approach has been previously shown to provide biological insights through reliable estimation of kinase activities from cell line perturbations^23^. Biological conditions with significant phosphosite activity (−log_10_ (*P* value) > 2 in either direction) in at least one subgroup were shown.

#### Disorder propensity, charge and hydrophobicity of the local segment around a phosphosite

Phosphosites identified from protein for which a substantial insoluble subpool was measured were used for the analysis described (related to Fig. 6). The presence of a phosphosite in a disordered segment of a protein (related to Fig. 6a) was assessed based on the predicted disordered regions, annotated in the D^2^P^2^ database. The physicochemical properties of the phosphosites were evaluated for the 31-amino acid segment (with the phosphosites as the center residue).

The 31-amino acid segments with low mean hydrophobicity and high mean net charge as described in ref. ^27^ were considered to be disordered. The net charge per residue (NPCR = fraction of positively charged residues (*f*_+_) + fraction of negatively charged residues (*f*_−_)), (FCR = *f*_+_ + *f*_−_) and *κ* (parameter that describes the extent of mixing of charged amino acids within a sequence (with well-mixed segments tending to have *κ* closer to 0 and segregated sequences having *κ* closer to 1) were calculated using the Python (version 3.7.4) module localCIDER (version 0.1.14)^46^. The proportions of aromatic (Y|F|W) and proline residues within these local segments were also computed. The distribution of these parameters was compared between phosphosites enriched in soluble and insoluble protein subpools as well as for phosphosites that did not differ in solubility.

### Image analysis

#### HNRNPA1 nuclear intensity measurements

The mean and s.d. of the nuclear intensity of HNRNPA1 and its phosphomutants were measured from single *z* slices of HeLa cell lines overexpressing GFP-tagged versions of the proteins. The GFP signal from the HNRNPA1 variants was used for segmenting nuclei with the local adaptive threshold using CellCognition Explorer^61^ of the GFP channel. The coefficient of variation (s.d. ÷ mean) of nuclear intensity was calculated per nucleus.

##### Sample numbers

For Fig. 4d,e, numbers of nuclei analyzed in two independent trials were as follows: WT (*n* = 45, 113), S2A–S4A–S6A (*n* = 58, 57), S2D–S4D–S6D (*n* = 34, 94), S362A–S365A (*n* = 26, 99), S362D–S365D (*n* = 36, 107), S361A–S362A–S363A–S364A–S265A–S368A (*n* = 36, 97), S361D–S362D–S363D–S364D–S265D–S368D (*n* = 42, 121).

#### NPM1 partition coefficient (*K*) measurements

Partition coefficient measurements were performed on single *z* slices of the HeLa cell line expressing WT GFP-tagged NPM1 from a BAC transiently expressing SiR-SNAP-tagged NPM1 mutants. Nucleoli were segmented using CellCognition Explorer^61^ based on local adaptive thresholding of the GFP channel. For intensity measurements of the nucleoplasm, a rim of 6 px (424 nm) surrounding each segmented nucleolus was generated using CellCognition Explorer’s Ring function (inner distance, 1; outer distance, 6). Background was measured in a 90 × 90-px ROI (6.36 × 6.36 µm) outside the cell area and subtracted from all intensity values of the corresponding image. Nucleoli with a size of more than 1,000 px^2^ (4.99 µm^2^) were considered for the analysis. Using R, the partition coefficient *K* was calculated by dividing the nucleolus mean intensity by the corresponding nucleoplasmic mean intensity. To ensure robust calculation of *K* values, we only considered cells with a mean nucleolus intensity 20 times higher than the average background values (GFP > 1.69 and SNAP > 0.77). The calculated partition coefficients of SiR-SNAP–NPM1 mutants were normalized to the median partition coefficient of WT SiR-SNAP–NPM1 of each independent experiment. We noticed that the *K* values calculated from SNAP were independent of NPM1 expression levels, while the *K* values calculated from GFP followed the trend previously observed in ref. ^11^.

##### Sample numbers

For Fig. 5d,f, numbers of nucleoli and nucleoplasms analyzed in at least three independent experiments per SiR-SNAP–NPM1 construct were as follows: WT (*n* = 205, 210, 161, 114, 135, 271, 1,128, 323, 433, 165, 586, 106, 411, 133), S4A–S10A (*n* = 133, 797, 191), S4D–S10D (*n* = 789, 369, 24), S218A–T219A (*n* = 372, 162, 380), S218D–T219E (*n* = 390, 216, 314), S254A–S260A (*n* = 504, 136, 355), S254D–S260D (*n* = 350, 243, 637), S218A–T219A–S254A–S260A (*n* = 161, 209, 99), S218D–T219E–S254D–S260D (*n* = 133, 456, 282, 232), S4A–S10A–S218A–T219A–S254A–S260A (*n* = 73, 628, 135), S4D–S10D–S218D–T219E–S254D–S260D (*n* = 368, 166, 127).

### Reporting summary

Further information on research design is available in the Nature Research Reporting Summary linked to this article.

## Online content

Any methods, additional references, Nature Research reporting summaries, source data, extended data, supplementary information, acknowledgements, peer review information; details of author contributions and competing interests; and statements of data and code availability are available at 10.1038/s41589-022-01062-y.

## Supplementary information

Reporting Summary

Supplementary Data 1 Protein solubility in RNA-preserved and RNA-digested lysates.

Supplementary Data 2 Classification of proteins based on solubility and their physiochemical properties.

Supplementary Data 3 Differential solubility of phosphopeptides.

Supplementary Data 4 Classification of phosphosites based on solubility and the RNA-bound fraction.

Supplementary Data 5 Partition coefficients of NPM1 and its phosphomutants.

Supplementary Data 6 NPM1 interactors.

## Data Availability

All raw MS data have been deposited in PRIDE. Data are available via ProteomeXchange with the identifier PXD027769. Source data are provided with this paper.

## Source data

Source Data Fig. 1 Statistical analysis source data.
Source Data Fig. 1 Unprocessed confocal image data.
Source Data Fig. 2 Statistical analysis source data.
Source Data Fig. 3 Calculated parameters of the 31-amino acid window surrounding a phosphosite.
Source Data Fig. 4 Statistical analysis source data.
Source Data Fig. 4 Unprocessed confocal image data.
Source Data Fig. 5 Statistical analysis source data.
Source Data Fig. 5 Unprocessed confocal images.
Source Data Fig. 6 Statistical analysis source data.
Source Data Extended Data Fig. 1 Statistical analysis source data.
Source Data Extended Data Fig. 1 Unprocessed confocal image data.
Source Data Extended Data Fig. 2 Statistical analysis source data.
Source Data Extended Data Fig. 2 Unprocessed images.
Source Data Extended Data Fig. 3 Statistical analysis source data.
Source Data Extended Data Fig. 4 Statistical analysis source data.
Source Data Extended Data Fig. 5 Statistical analysis source data.
Source Data Extended Data Fig. 6 Statistical analysis source data.
Source Data Extended Data Fig. 7 Statistical analysis source data.
Source Data Extended Data Fig. 8 Statistical analysis source data.
Source Data Extended Data Fig. 8 Unprocessed gel image.
