# Supplementary material for: Systematic discovery of biomolecular condensate-specific protein phosphorylation
Source: Nat Chem Biol. 2022 Jul 21;18(10):1104–14. doi: 10.1038/s41589-022-01062-y (PMC9512703; doi:10.1038/s41589-022-01062-y)
Supplement: Supplementary file 1 — Reporting Summary [file 41589_2022_1062_MOESM1_ESM.pdf]

nature research

Corresponding author(s): *Double-blind peer review submissions: write DBPR and your manuscript number here instead of author names.*

Last updated by author(s): *YYYY-MM-DD*

Reporting Summary

Nature Research wishes to improve the reproducibility of the work that we publish. This form provides structure for consistency and transparency in reporting. For further information on Nature Research policies, see our Editorial Policies and the Editorial Policy Checklist.

Statistics

For all statistical analyses, confirm that the following items are present in the figure legend, table legend, main text, or Methods section.

n/a	Confirmed	
☐	☒	The exact sample size (*n*) for each experimental group/condition, given as a discrete number and unit of measurement
☐	☒	A statement on whether measurements were taken from distinct samples or whether the same sample was measured repeatedly
☐	☒	The statistical test(s) used AND whether they are one- or two-sided<br>*Only common tests should be described solely by name; describe more complex techniques in the Methods section.*
☒	☐	A description of all covariates tested
☐	☒	A description of any assumptions or corrections, such as tests of normality and adjustment for multiple comparisons
☒	☐	A full description of the statistical parameters including central tendency (e.g. means) or other basic estimates (e.g. regression coefficient) AND variation (e.g. standard deviation) or associated estimates of uncertainty (e.g. confidence intervals)
☐	☒	For null hypothesis testing, the test statistic (e.g. *F*, *t*, *r*) with confidence intervals, effect sizes, degrees of freedom and *P* value noted<br>*Give P values as exact values whenever suitable.*
☒	☐	For Bayesian analysis, information on the choice of priors and Markov chain Monte Carlo settings
☒	☐	For hierarchical and complex designs, identification of the appropriate level for tests and full reporting of outcomes
☒	☐	Estimates of effect sizes (e.g. Cohen's *d*, Pearson's *r*), indicating how they were calculated

Our web collection on statistics for biologists contains articles on many of the points above.

Software and code

Policy information about availability of computer code

Data collection	All mass spectrometry data collection was been performed on Q Exactive Orbitrap or Orbitrap Fusion Lumos mass spectrometers (Thermo Fischer Scientific) and commercial operating software (Xcalibur) from Thermo Fischer Scientific was used.<br>Isobarquant (version 1.1.0) - MS data search<br>MaxQuant software (version 1.6.15)<br>Confocal images were obtained on Zeiss 780 microscope operated through ZEN 2011 software<br>RNA samples were measured on 2100 Bioanalyzer instrument (Agilent) programmed using 2100 Expert software
Data analysis	MS Data search: Mascot (Matrix Science) was used for peptide searching and isobarQuant (https://github.com/protcode/isob/archive/1.1.0.zip) was used to quantify peptide and protein abundances. For Phosphorylation site assignment peptide and protein search was carried out on Maxquant (version 1.6.15) and combined with Isobarquant output.<br>All statistical data analysis was performed on R studio (version 1.2.1335) running R (version 3.6.1).<br>Image segmentation and quantification was perfomed on Cell Cognition Explorer (version 1.02)<br>Image analysis was performed using ImageJ 1.53e

For manuscripts utilizing custom algorithms or software that are central to the research but not yet described in published literature, software must be made available to editors and reviewers. We strongly encourage code deposition in a community repository (e.g. GitHub). See the Nature Research guidelines for submitting code & software for further information.

Data

Policy information about availability of data

All manuscripts must include a data availability statement. This statement should provide the following information, where applicable:

- Accession codes, unique identifiers, or web links for publicly available datasets
- A list of figures that have associated raw data
- A description of any restrictions on data availability

All raw mass spectrometry data have been deposited on proteomeXchange Consortium via the PRIDE partner repository with datset identifier PXD027769.
Proteomics datasets were search using Uniprot Reference proteome (UP000005640, version downloaded on 14 May 2016)
Data for gene ontology (molecular function and cellular localization) assignment was obtained using Bioconductor package org.Hs.eg.db (version 3.8.2).
Data for protein domain was obtained from Pfam database (version 33.1) and the enrichment analysis was performed using DAVID (version 6.8)
Data for kinase-substate relationship was obtained from https://github.com/indralab/protmapper
Data for kinase activity assessment was obtained from http://phosfate.com/
Data for phase separation propensity of a protein was obtained from PhaseDB (version 1.0)
Information of predicted disordered regions of a protein was obtained from D2P2 database
Sequence properties of local disorder segments were calculated using localCIDER package(version 0.1.14) using python (version 3.7.4)

Field-specific reporting

Please select the one below that is the best fit for your research. If you are not sure, read the appropriate sections before making your selection.

☒ Life sciences ☐ Behavioural & social sciences ☐ Ecological, evolutionary & environmental sciences

For a reference copy of the document with all sections, see nature.com/documents/nr-reporting-summary-flat.pdf

Life sciences study design

All studies must disclose on these points even when the disclosure is negative.

Sample size	Biological triplicates have been used in the study to gain appropriate power for discovery of true positive events
Data exclusions	No data was excluded for the analysis presented in this study
Replication	Data interpretation and conclusions were drawn from reproducible effects from the triplicate datasets
Randomization	DIfferent passages of cells were used for different replicates. Cell position for controls and samples were randomized between different replicates for transfection related experiments.
Blinding	No blinding was performed, since it was necessary to track the control and test samples which were always compared in parallel.

Reporting for specific materials, systems and methods

We require information from authors about some types of materials, experimental systems and methods used in many studies. Here, indicate whether each material, system or method listed is relevant to your study. If you are not sure if a list item applies to your research, read the appropriate section before selecting a response.

Materials & experimental systems

n/a	Involved in the study	
☐	☒	Antibodies
☐	☒	Eukaryotic cell lines
☒	☐	Palaeontology and archaeology
☒	☐	Animals and other organisms
☒	☐	Human research participants
☒	☐	Clinical data
☒	☐	Dual use research of concern

Methods

n/a	Involved in the study	
☒	☐	ChIP-seq
☒	☐	Flow cytometry
☒	☐	MRI-based neuroimaging

Antibodies

Antibodies used	Mouse monoclonal IgG against NPM1 (sc-32256, Santa Cruz Biotechnology, clone: FC-8791)

Antibodies used	Goat-anti-mouse IgG-HRP (sc-2005, Santa Cruz Biotechnology)
Validation	The above mentioned antibosy has been used in multiple studies for detection of NPM1<br>https://www.scbt.com/p/b23-antibody-fc-8791

Eukaryotic cell lines

Policy information about cell lines

Cell line source(s)	Hela-Kyoto (Schmitz et al., 2010) - was a gift from Jan Ellenberg's group, EMBL - original cell line (RRID: CVCL_1922) was received as gift from Prof. S.Narumiya, Kyoto University and authenticated by sequencing.<br>Hela cells overexpressing wildtype GFP-NPM1 as a bacterial artifical chromosome (BAC-line) (Poser et al., 2008) - cell line gifted by Hyman group, MPI, Dresden
Authentication	HeLa cells have been authenticated by sequencing.
Mycoplasma contamination	verified for contamination of mycoplasma, none detected.
Commonly misidentified lines<br>(See ICLAC register)	To the best of our knowledge no misidentified cell lines have been used in this study.
