# Supplementary figures and images for: Systematic discovery of biomolecular condensate-specific protein phosphorylation
Source: Nat Chem Biol. 2022 Jul 21;18(10):1104–14. doi: 10.1038/s41589-022-01062-y (PMC9512703; doi:10.1038/s41589-022-01062-y)

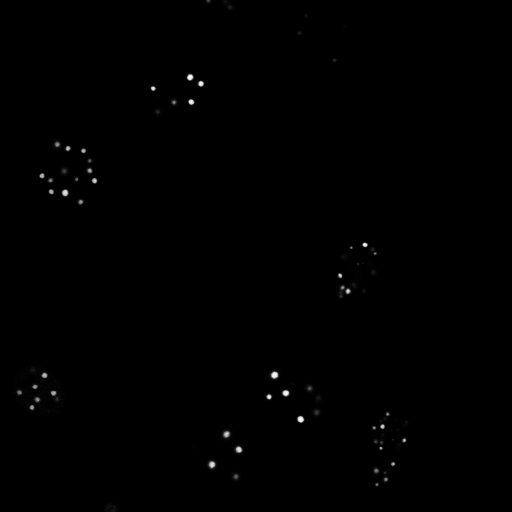

Supplement: Source Data Fig. 1 — Unprocessed confocal image data. [file 41589_2022_1062_MOESM9_ESM.zip › coil_incell.jpg]

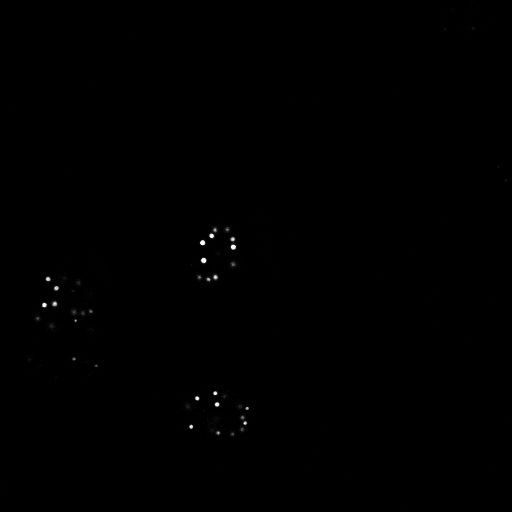

Supplement: Source Data Fig. 1 — Unprocessed confocal image data. [file 41589_2022_1062_MOESM9_ESM.zip › coil_permeabilized.jpg]

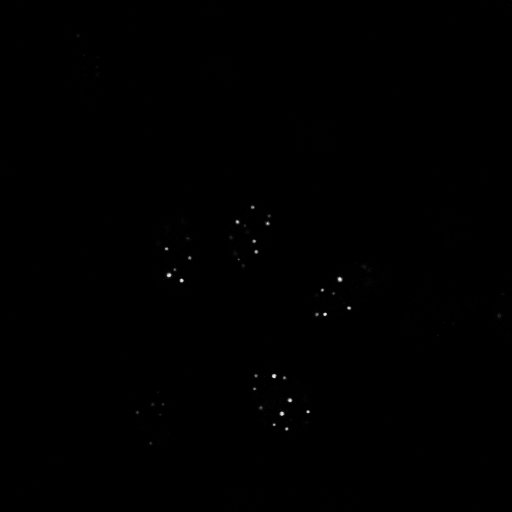

Supplement: Source Data Fig. 1 — Unprocessed confocal image data. [file 41589_2022_1062_MOESM9_ESM.zip › coil_rnase_permeabilized.jpg]

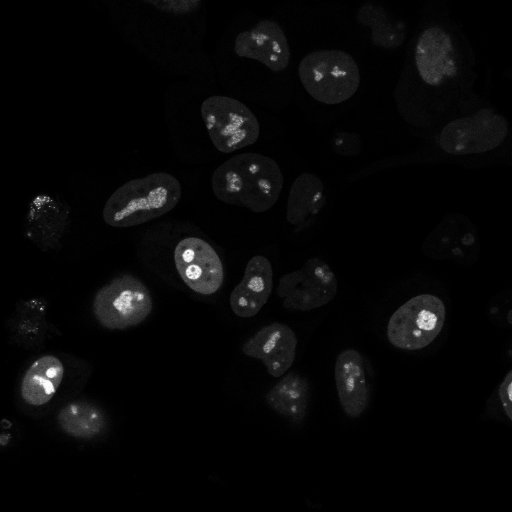

Supplement: Source Data Fig. 1 — Unprocessed confocal image data. [file 41589_2022_1062_MOESM9_ESM.zip › fbl_incell.jpg]

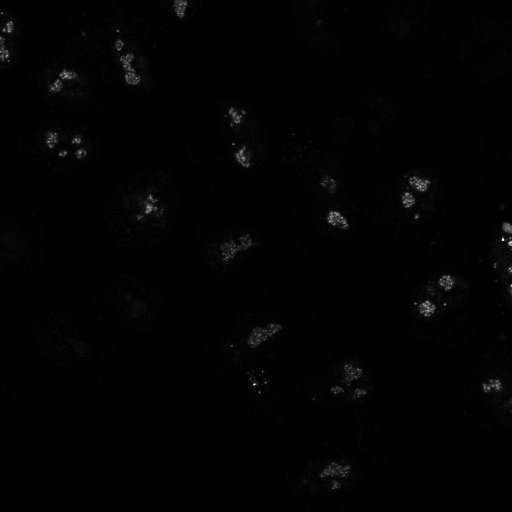

Supplement: Source Data Fig. 1 — Unprocessed confocal image data. [file 41589_2022_1062_MOESM9_ESM.zip › fbl_permeabilized.jpg]

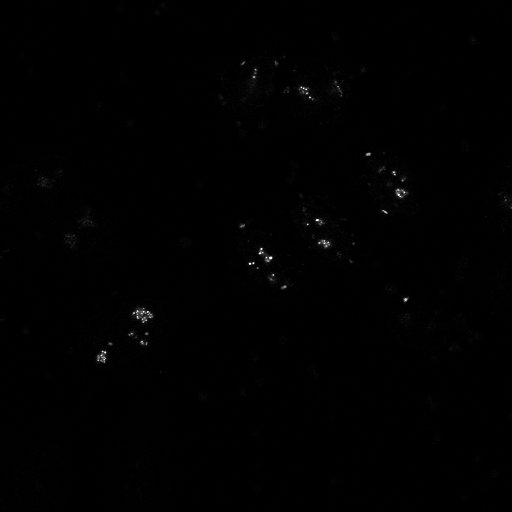

Supplement: Source Data Fig. 1 — Unprocessed confocal image data. [file 41589_2022_1062_MOESM9_ESM.zip › fbl_rnase_permeabilized.jpg]

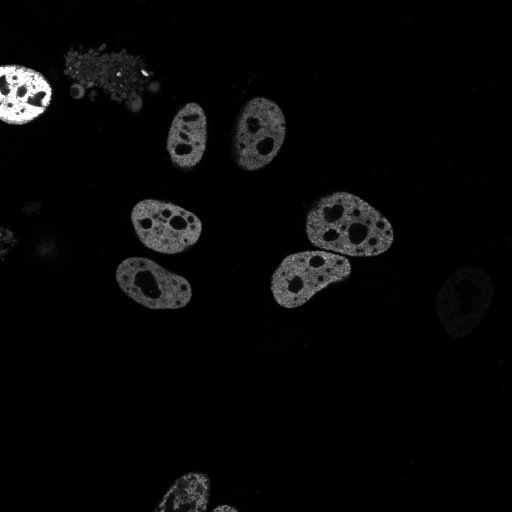

Supplement: Source Data Fig. 1 — Unprocessed confocal image data. [file 41589_2022_1062_MOESM9_ESM.zip › hnrnpa1_incell.jpg]

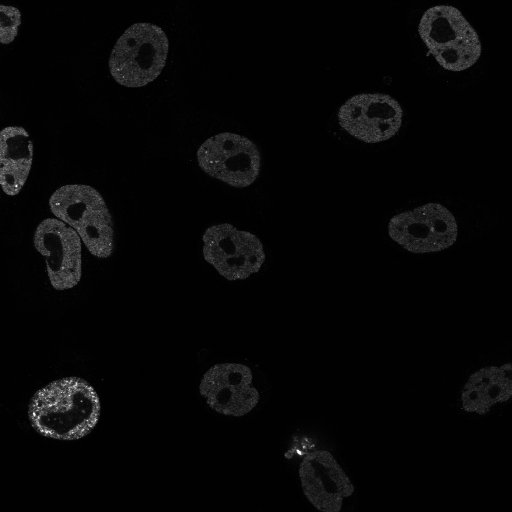

Supplement: Source Data Fig. 1 — Unprocessed confocal image data. [file 41589_2022_1062_MOESM9_ESM.zip › hnrnpa1_permeabilized.jpg]

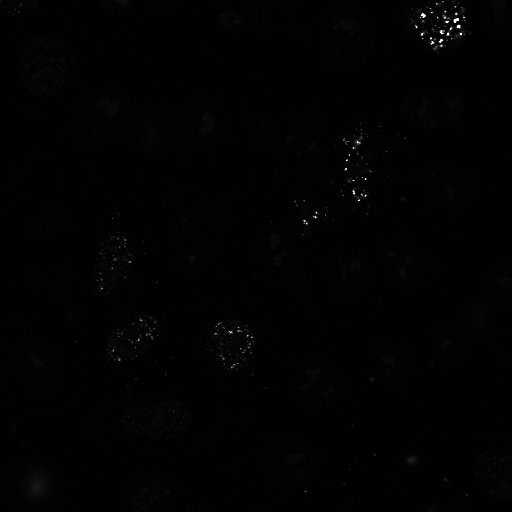

Supplement: Source Data Fig. 1 — Unprocessed confocal image data. [file 41589_2022_1062_MOESM9_ESM.zip › hnrnpa1_rnase_permeabilized.jpg]

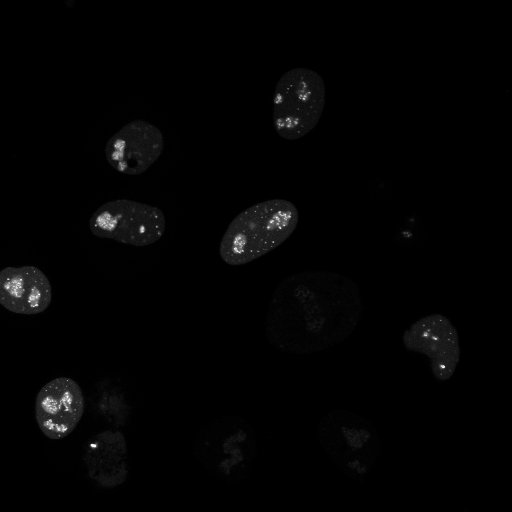

Supplement: Source Data Fig. 1 — Unprocessed confocal image data. [file 41589_2022_1062_MOESM9_ESM.zip › nop56_incell.jpg]

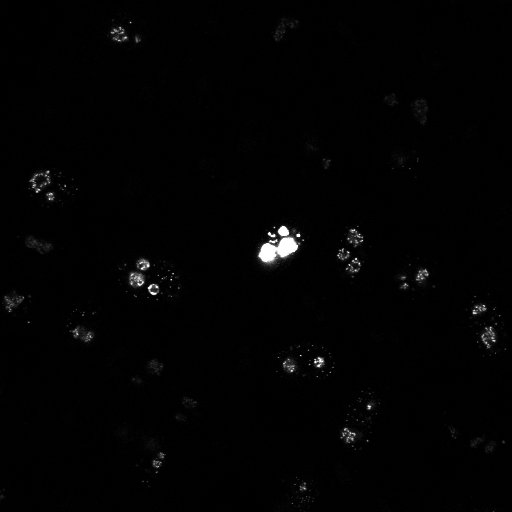

Supplement: Source Data Fig. 1 — Unprocessed confocal image data. [file 41589_2022_1062_MOESM9_ESM.zip › nop56_permeabilized.jpg]

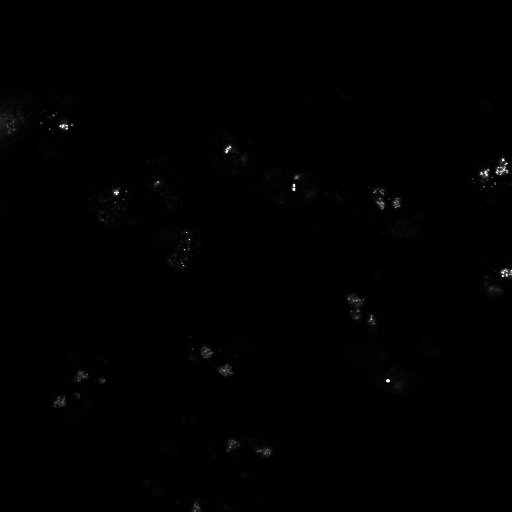

Supplement: Source Data Fig. 1 — Unprocessed confocal image data. [file 41589_2022_1062_MOESM9_ESM.zip › nop56l_rnase_permeabilized.jpg]

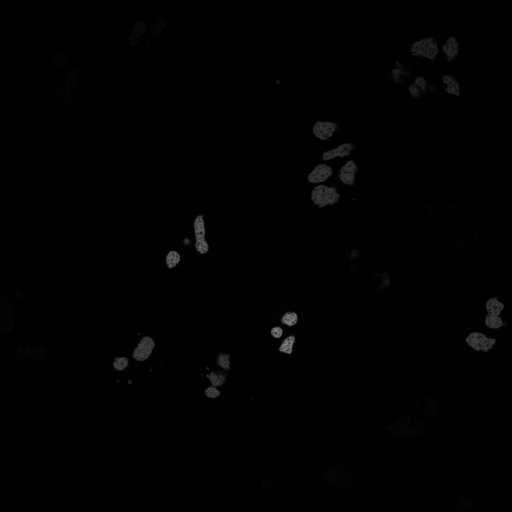

Supplement: Source Data Fig. 1 — Unprocessed confocal image data. [file 41589_2022_1062_MOESM9_ESM.zip › npm1_incell.jpg]

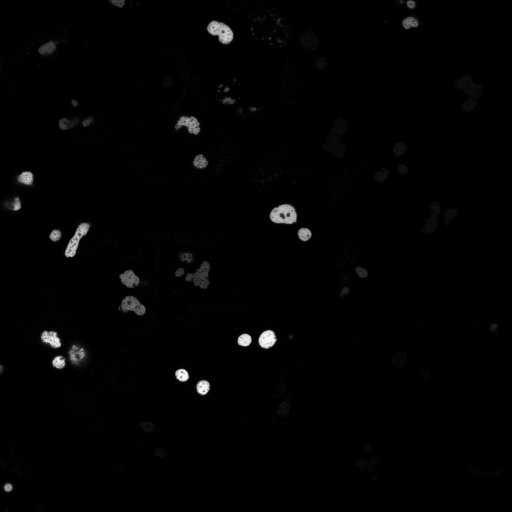

Supplement: Source Data Fig. 1 — Unprocessed confocal image data. [file 41589_2022_1062_MOESM9_ESM.zip › npm1_permeabilised.jpg]

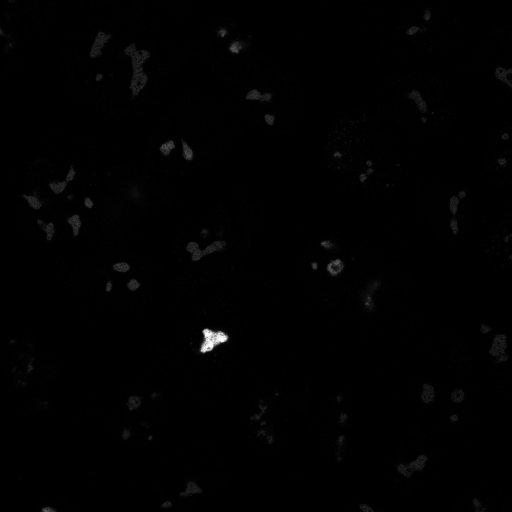

Supplement: Source Data Fig. 1 — Unprocessed confocal image data. [file 41589_2022_1062_MOESM9_ESM.zip › npml_rnase_permeabilized.jpg]

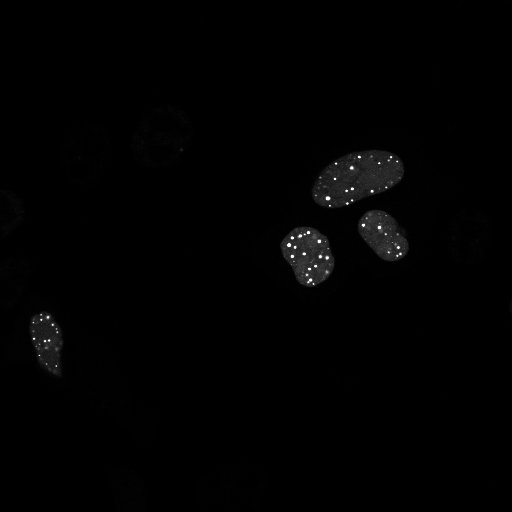

Supplement: Source Data Fig. 1 — Unprocessed confocal image data. [file 41589_2022_1062_MOESM9_ESM.zip › prpf6_incell.jpg]

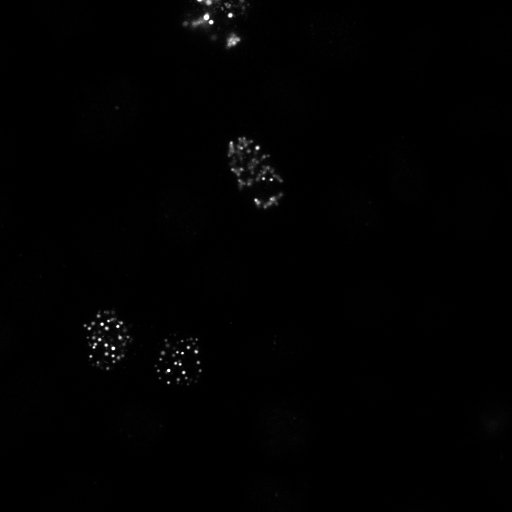

Supplement: Source Data Fig. 1 — Unprocessed confocal image data. [file 41589_2022_1062_MOESM9_ESM.zip › prpf6_permeabilized.jpg]

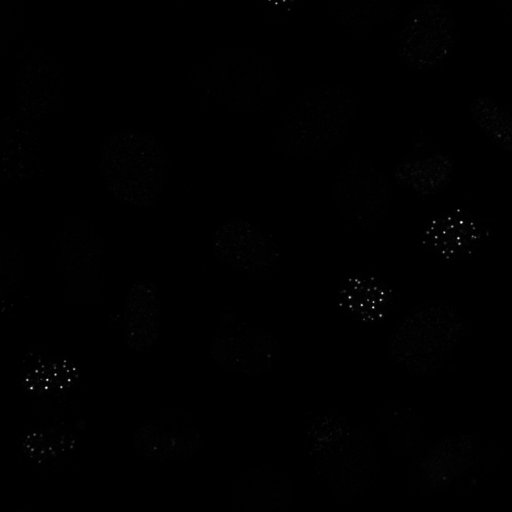

Supplement: Source Data Fig. 1 — Unprocessed confocal image data. [file 41589_2022_1062_MOESM9_ESM.zip › prpf6_rnase_permeabilized.jpg]

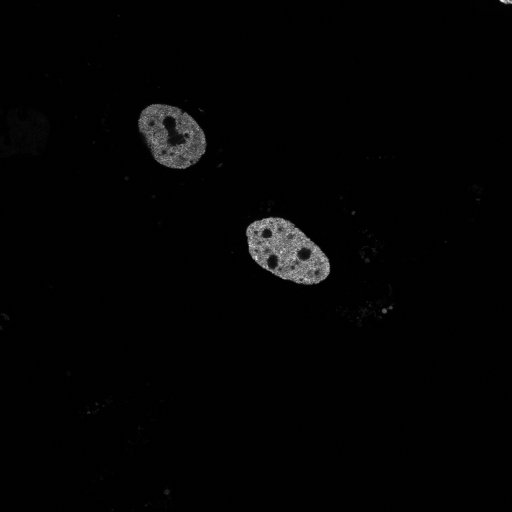

Supplement: Source Data Fig. 4 — Unprocessed confocal image data. [file 41589_2022_1062_MOESM13_ESM.zip › HNRNPA1_Cterm_2sites_deficient.jpg]

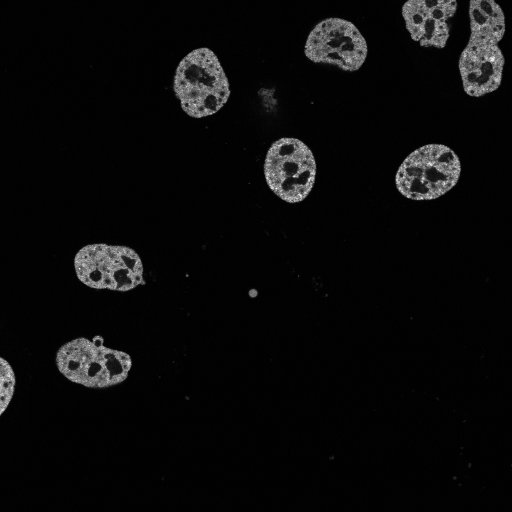

Supplement: Source Data Fig. 4 — Unprocessed confocal image data. [file 41589_2022_1062_MOESM13_ESM.zip › HNRNPA1_Cterm_2sites_mimetic.jpg]

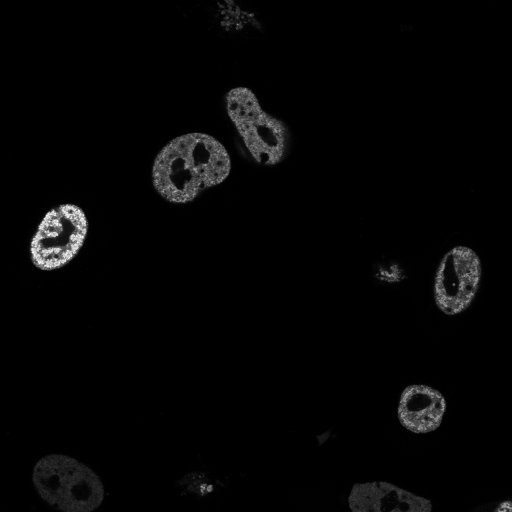

Supplement: Source Data Fig. 4 — Unprocessed confocal image data. [file 41589_2022_1062_MOESM13_ESM.zip › HNRNPA1_Cterm_6sites_deficient.jpg]

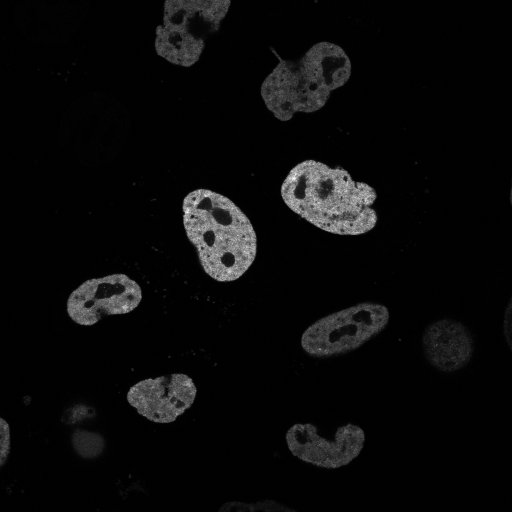

Supplement: Source Data Fig. 4 — Unprocessed confocal image data. [file 41589_2022_1062_MOESM13_ESM.zip › HNRNPA1_Cterm_6sites_mimetic.jpg]

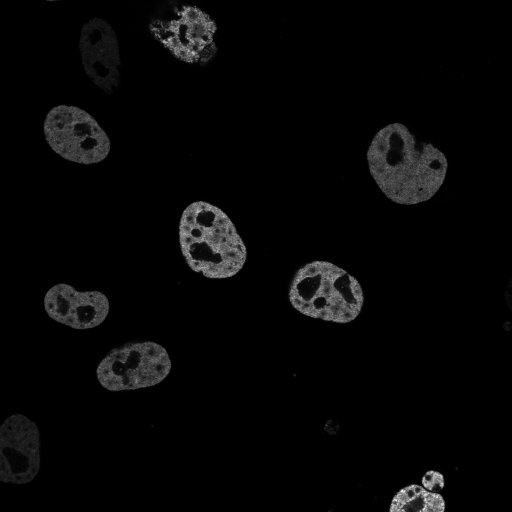

Supplement: Source Data Fig. 4 — Unprocessed confocal image data. [file 41589_2022_1062_MOESM13_ESM.zip › HNRNPA1_N_deficient.jpg]

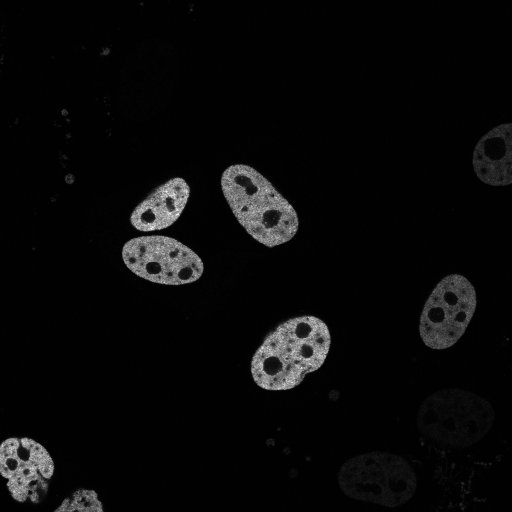

Supplement: Source Data Fig. 4 — Unprocessed confocal image data. [file 41589_2022_1062_MOESM13_ESM.zip › HNRNPA1_N_mimetic.jpg]

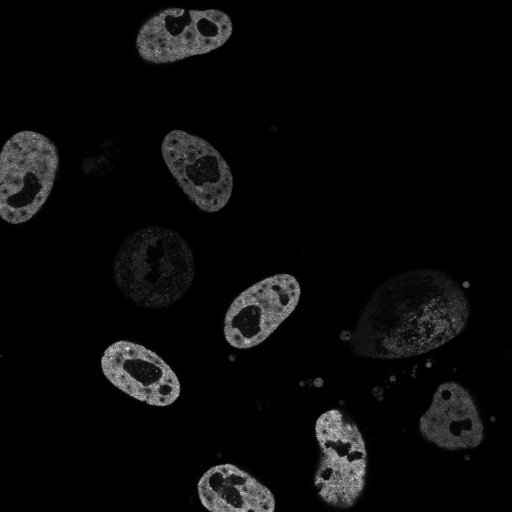

Supplement: Source Data Fig. 4 — Unprocessed confocal image data. [file 41589_2022_1062_MOESM13_ESM.zip › wt-HNRNPA1.jpg]

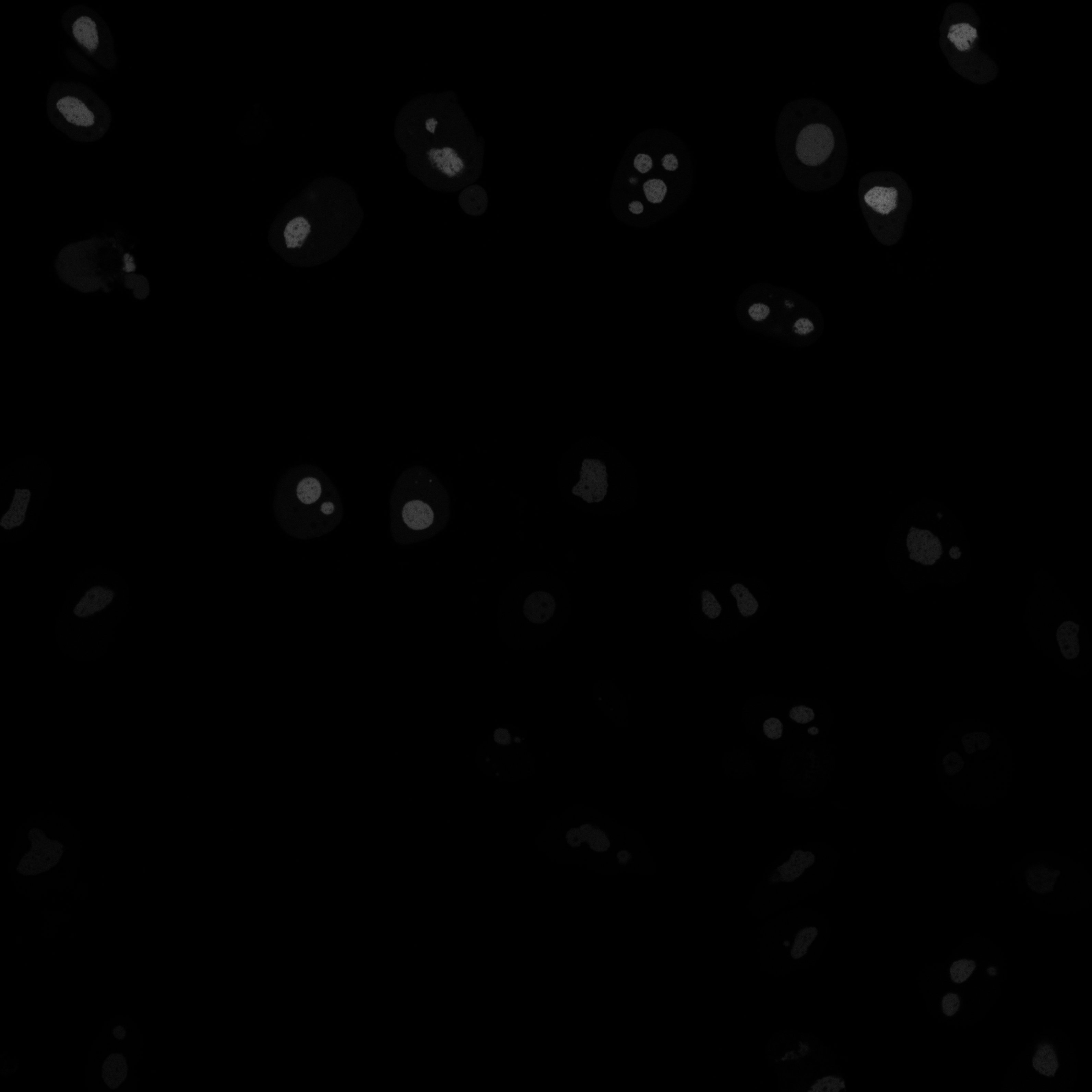

Supplement: Source Data Fig. 5 — Unprocessed confocal images. [file 41589_2022_1062_MOESM15_ESM.zip › S218A-T219A-S254A-S260A.jpg]

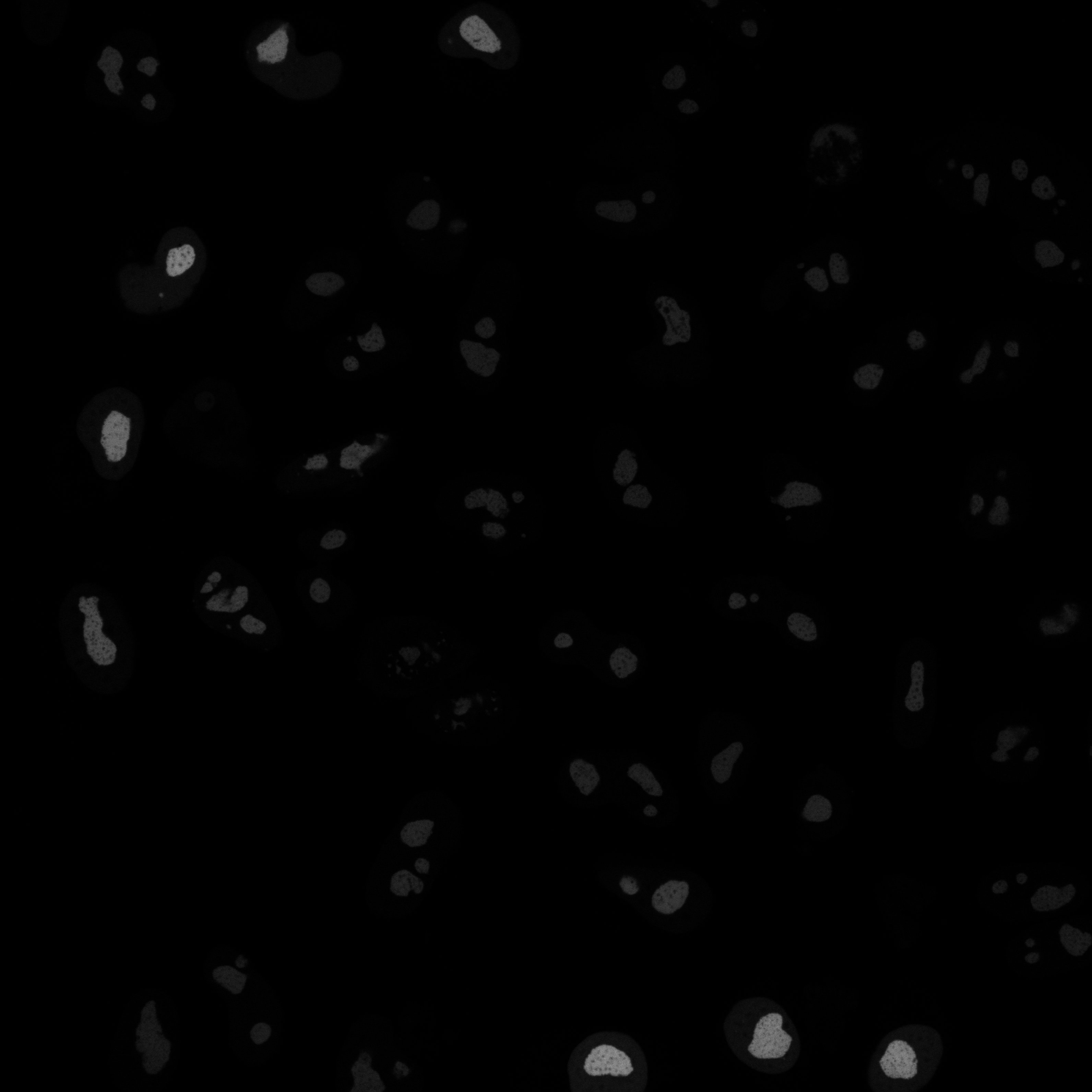

Supplement: Source Data Fig. 5 — Unprocessed confocal images. [file 41589_2022_1062_MOESM15_ESM.zip › S218A-T219A.jpg]

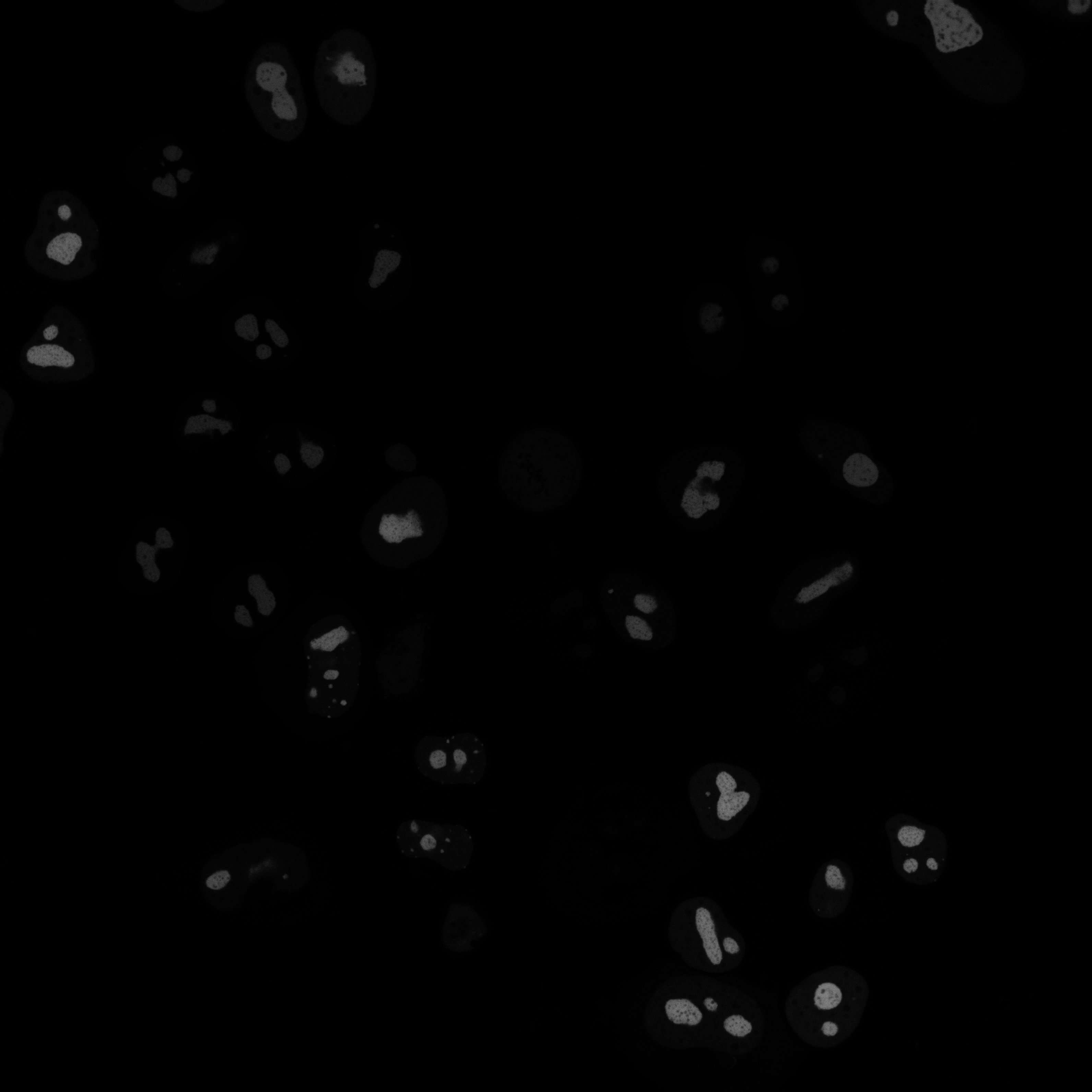

Supplement: Source Data Fig. 5 — Unprocessed confocal images. [file 41589_2022_1062_MOESM15_ESM.zip › S218D-T219E-S254D-S260D.jpg]

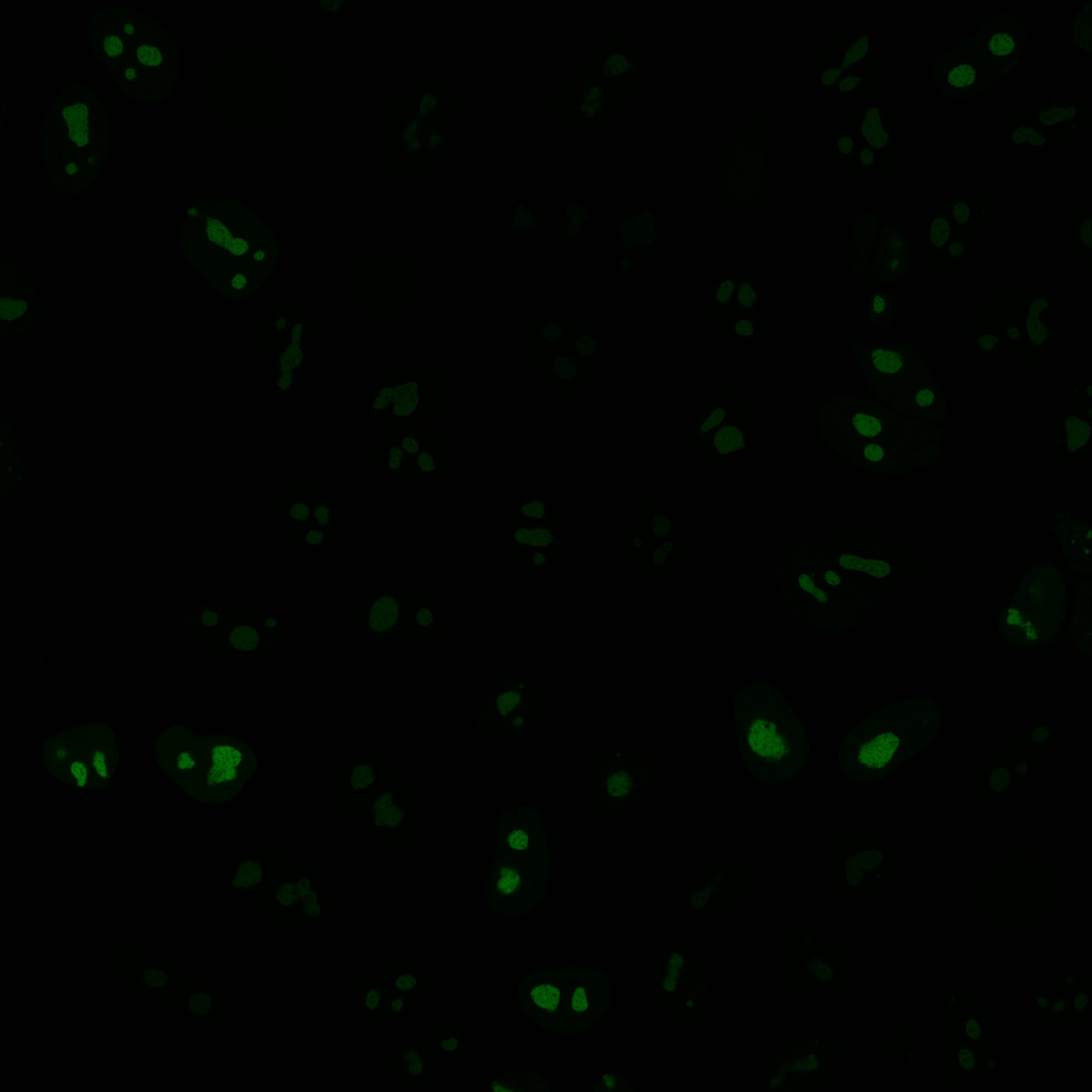

Supplement: Source Data Fig. 5 — Unprocessed confocal images. [file 41589_2022_1062_MOESM15_ESM.zip › S218D-T219E.jpg]

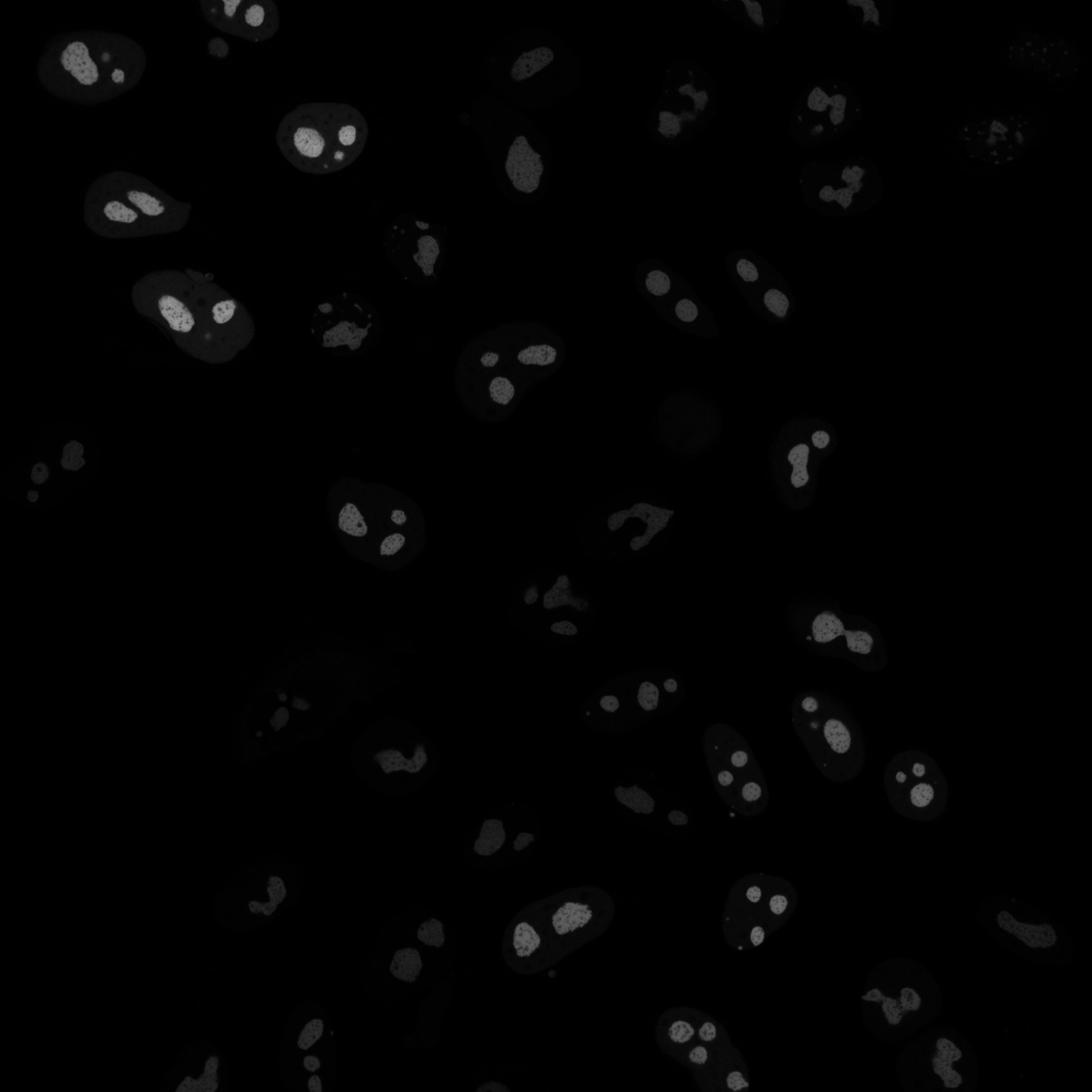

Supplement: Source Data Fig. 5 — Unprocessed confocal images. [file 41589_2022_1062_MOESM15_ESM.zip › S254A-S260A.jpg]

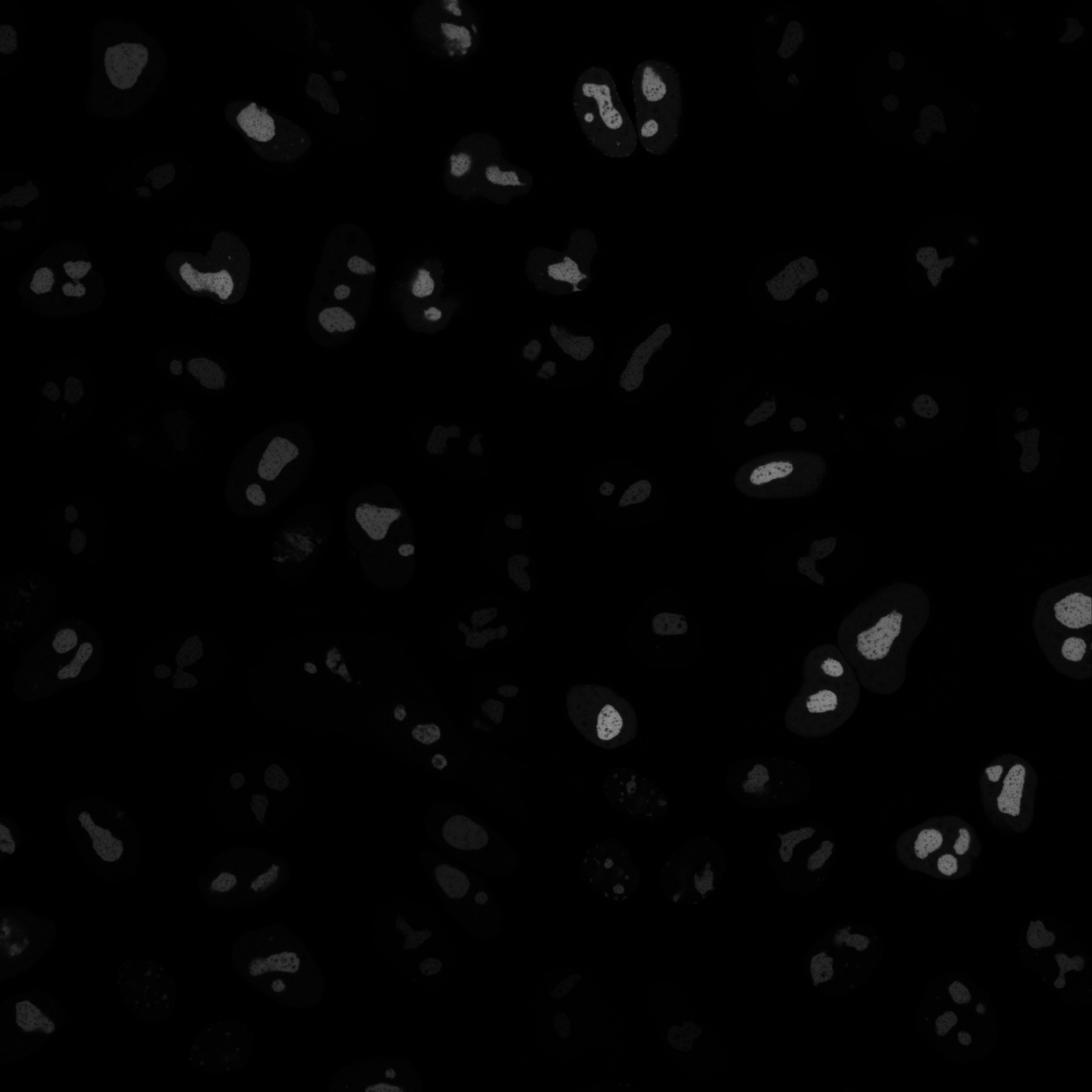

Supplement: Source Data Fig. 5 — Unprocessed confocal images. [file 41589_2022_1062_MOESM15_ESM.zip › S254D-S260D.jpg]

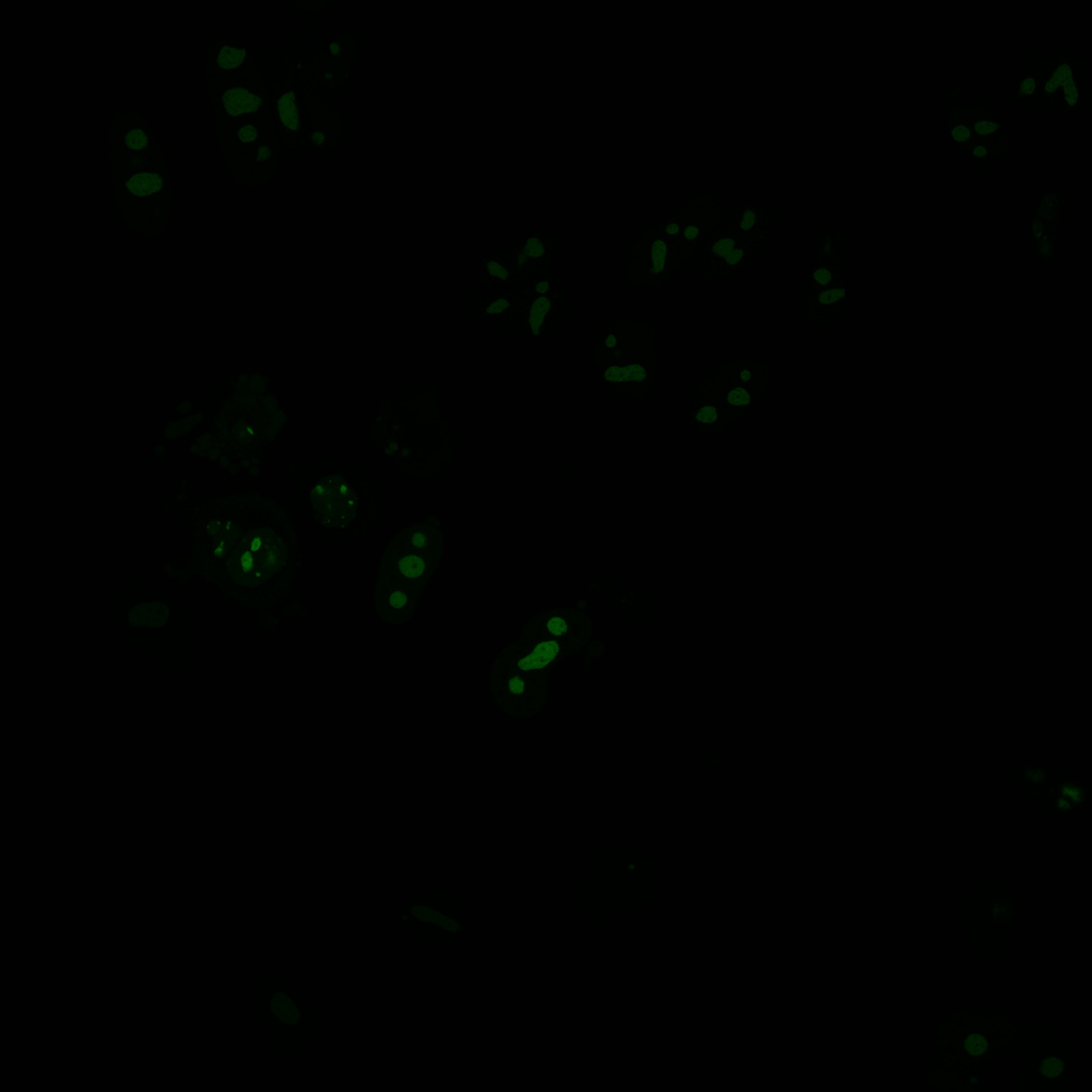

Supplement: Source Data Fig. 5 — Unprocessed confocal images. [file 41589_2022_1062_MOESM15_ESM.zip › S4A-S10A-S218A-T219A-S254A-S260A.jpg]

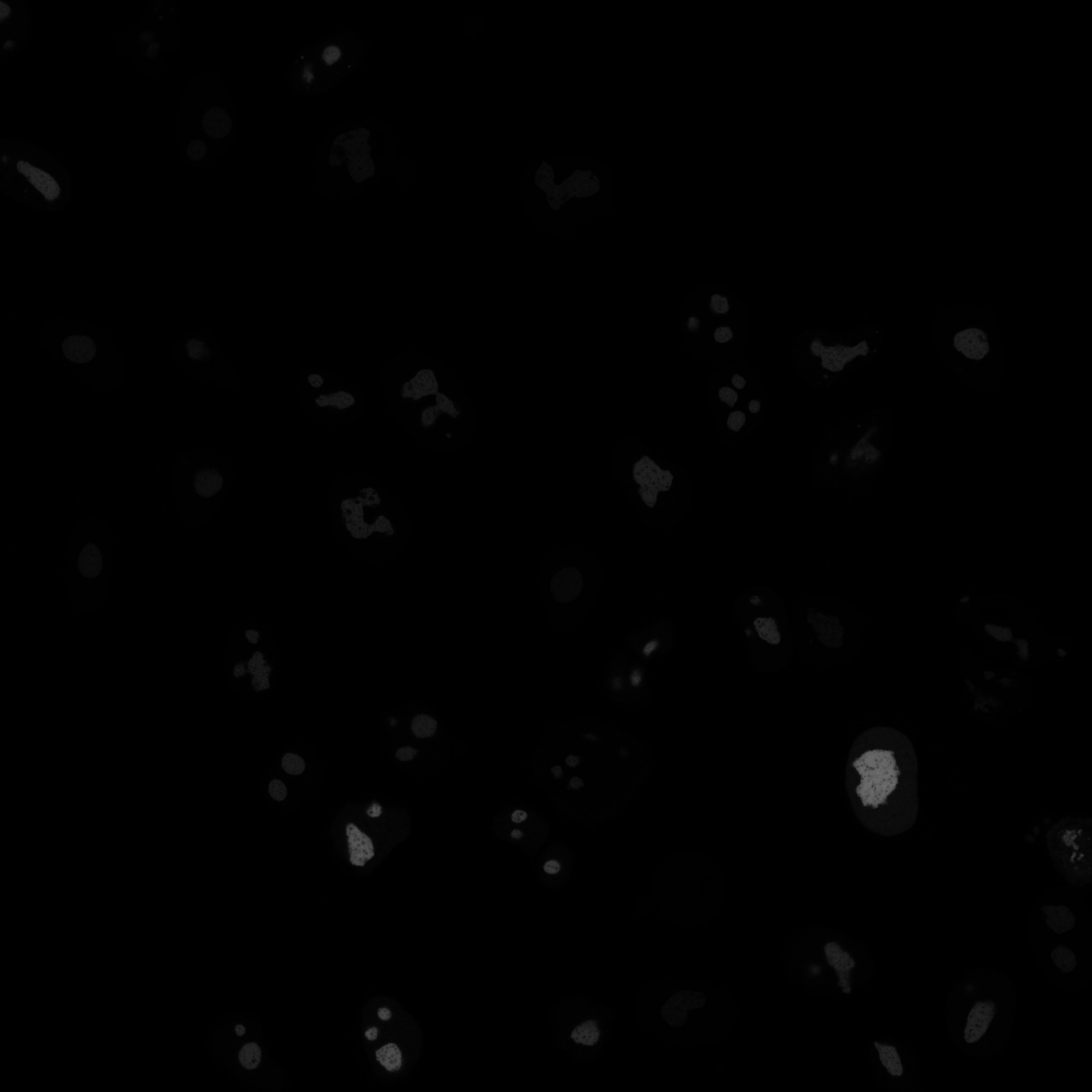

Supplement: Source Data Fig. 5 — Unprocessed confocal images. [file 41589_2022_1062_MOESM15_ESM.zip › S4A-S10A.jpg]

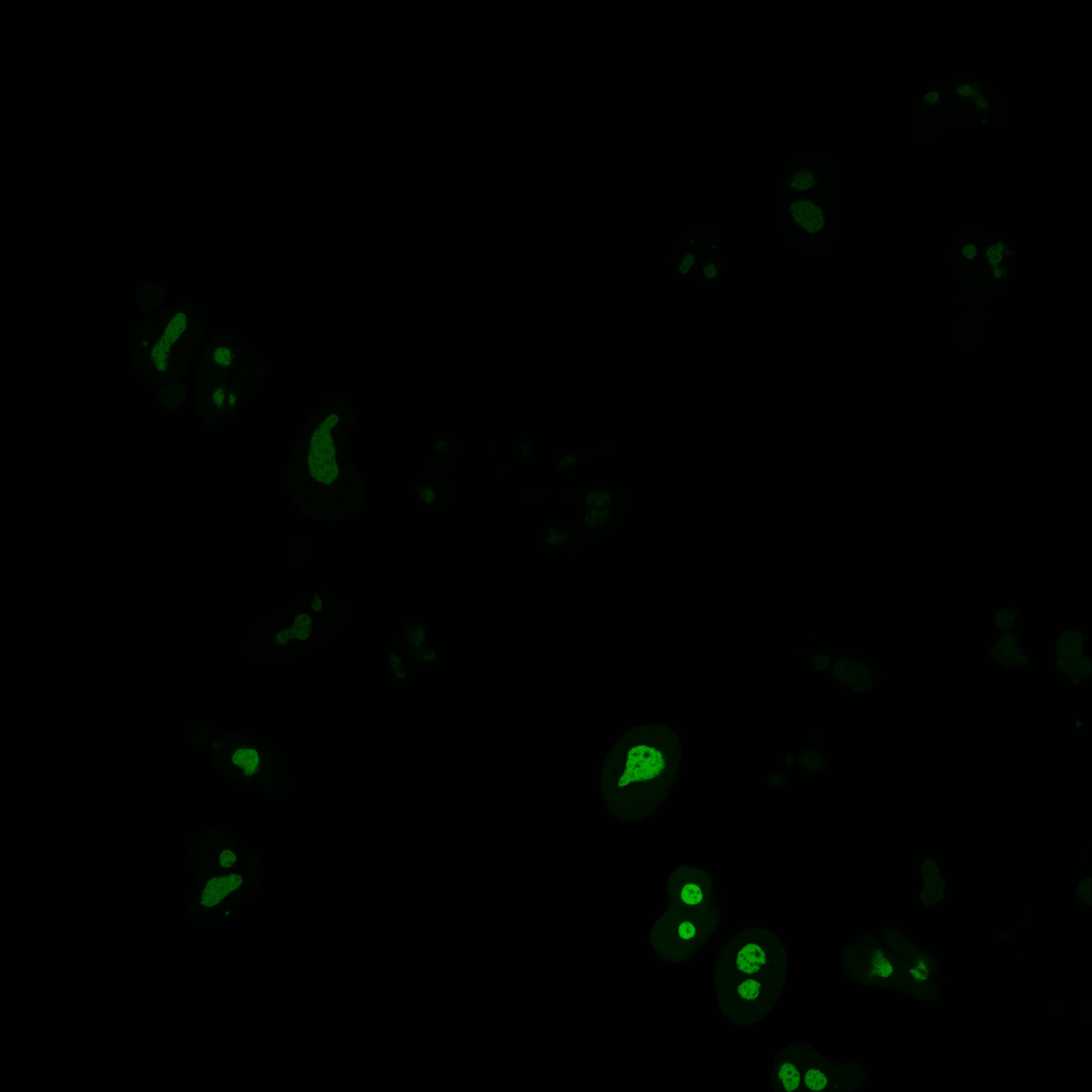

Supplement: Source Data Fig. 5 — Unprocessed confocal images. [file 41589_2022_1062_MOESM15_ESM.zip › S4D-S10D-S218D-T219E-S254D-S260D.jpg]

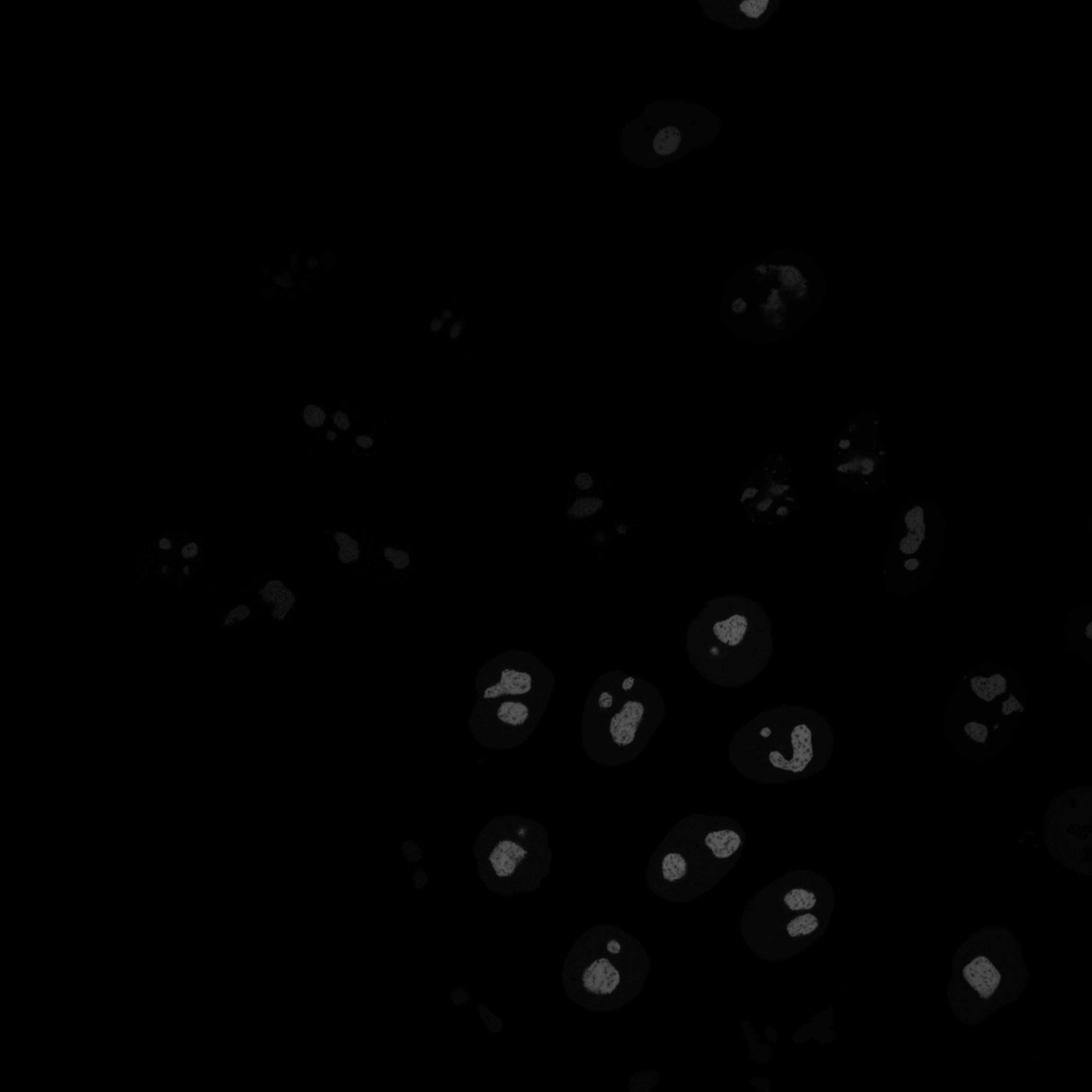

Supplement: Source Data Fig. 5 — Unprocessed confocal images. [file 41589_2022_1062_MOESM15_ESM.zip › S4D-S10D.jpg]

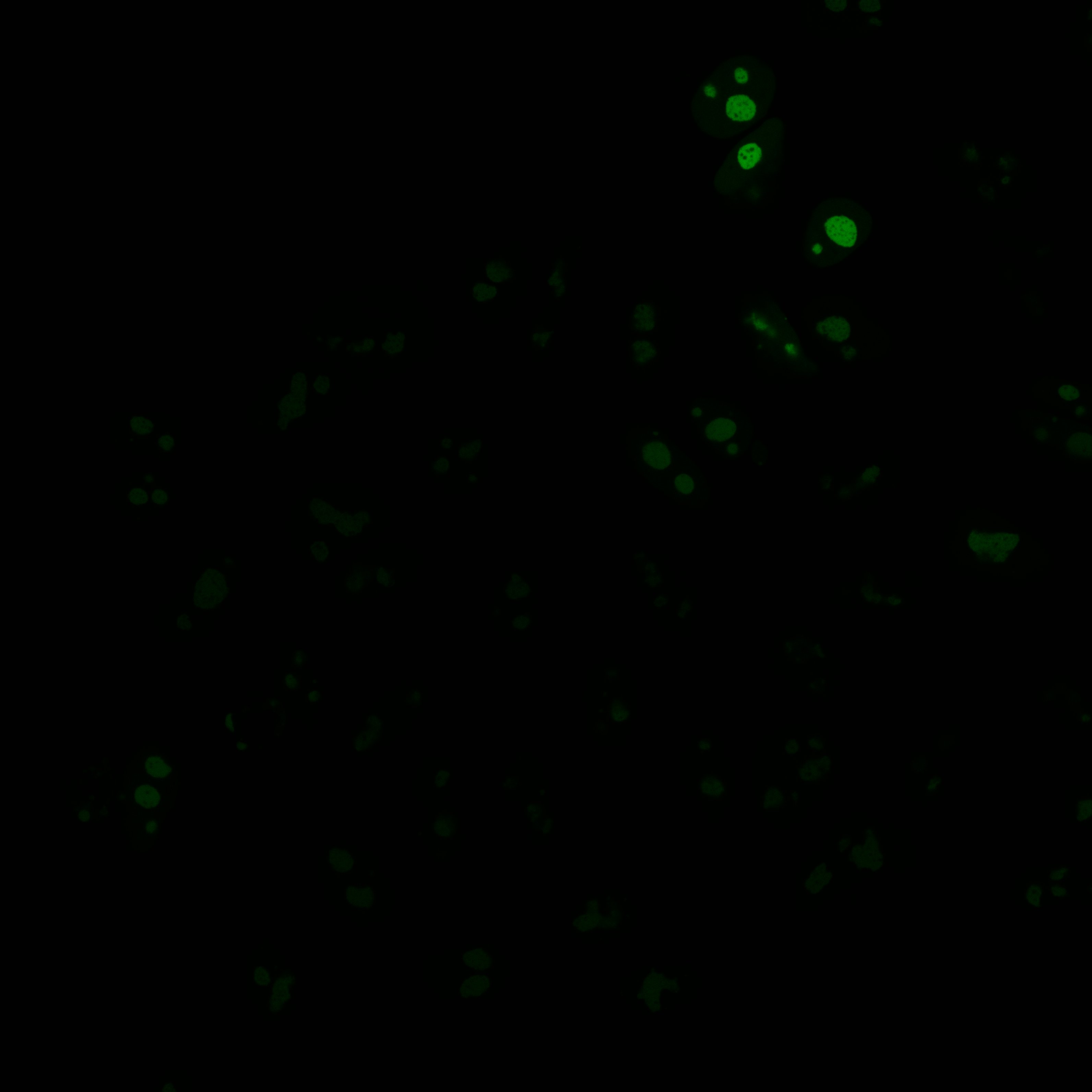

Supplement: Source Data Fig. 5 — Unprocessed confocal images. [file 41589_2022_1062_MOESM15_ESM.zip › WT.jpg]

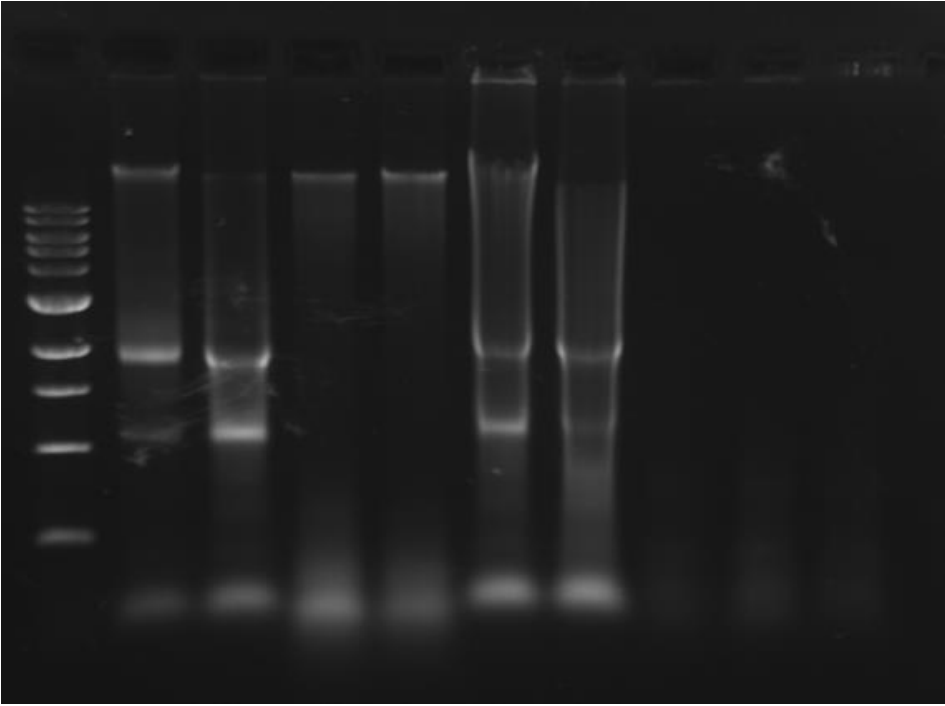

Supplement: Source Data Extended Data Fig. 1 — Unprocessed confocal image data. [file 41589_2022_1062_MOESM18_ESM.pdf]

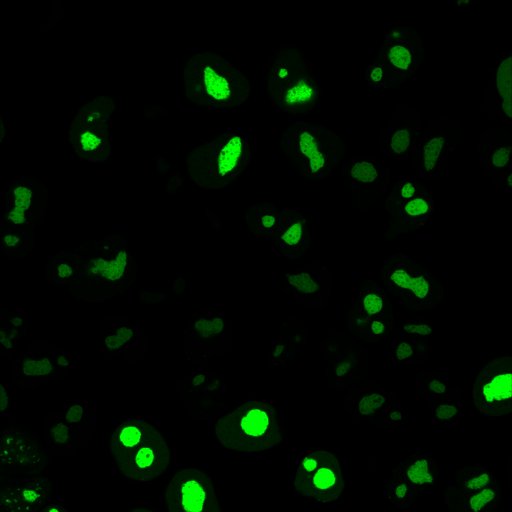

Supplement: Source Data Extended Data Fig. 2 — Unprocessed images. [file 41589_2022_1062_MOESM20_ESM.zip › C1-e-0891_HeLa-Kyoto_NPM1.jpg]

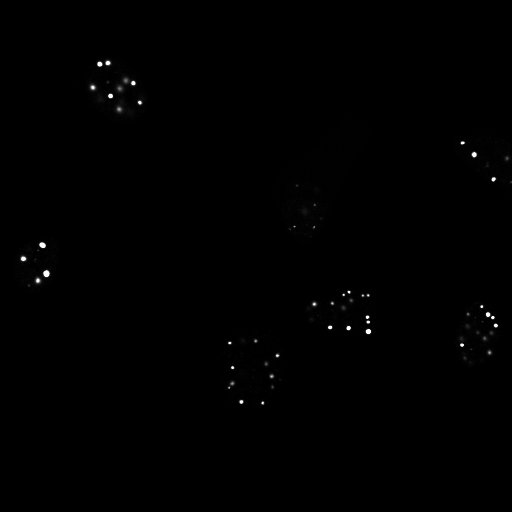

Supplement: Source Data Extended Data Fig. 2 — Unprocessed images. [file 41589_2022_1062_MOESM20_ESM.zip › e-0891_HeLa-Kyoto_COIL.jpg]

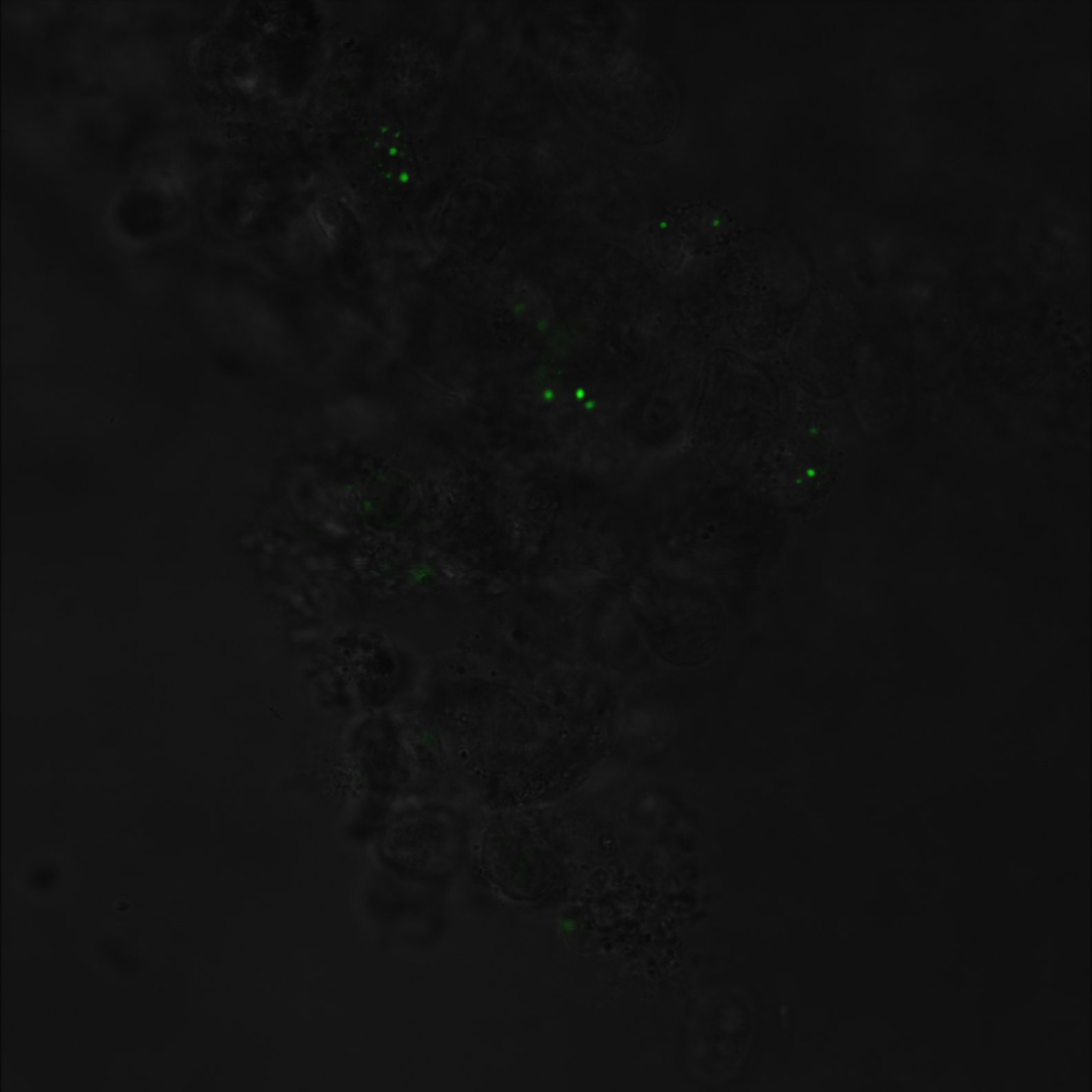

Supplement: Source Data Extended Data Fig. 2 — Unprocessed images. [file 41589_2022_1062_MOESM20_ESM.zip › e-0891_HeLa-Kyoto_COIL_lysate08.jpg]

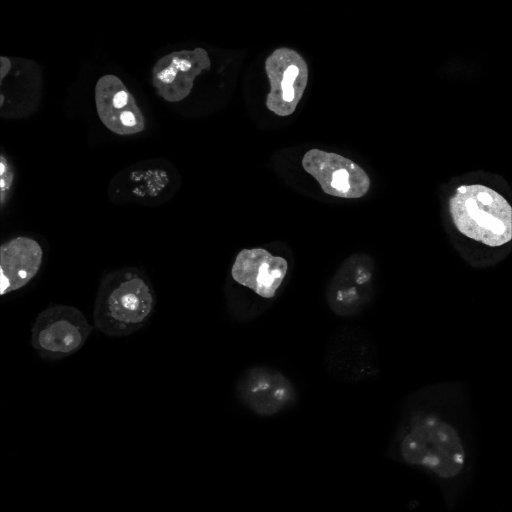

Supplement: Source Data Extended Data Fig. 2 — Unprocessed images. [file 41589_2022_1062_MOESM20_ESM.zip › e-0891_HeLa-Kyoto_FBL.jpg]

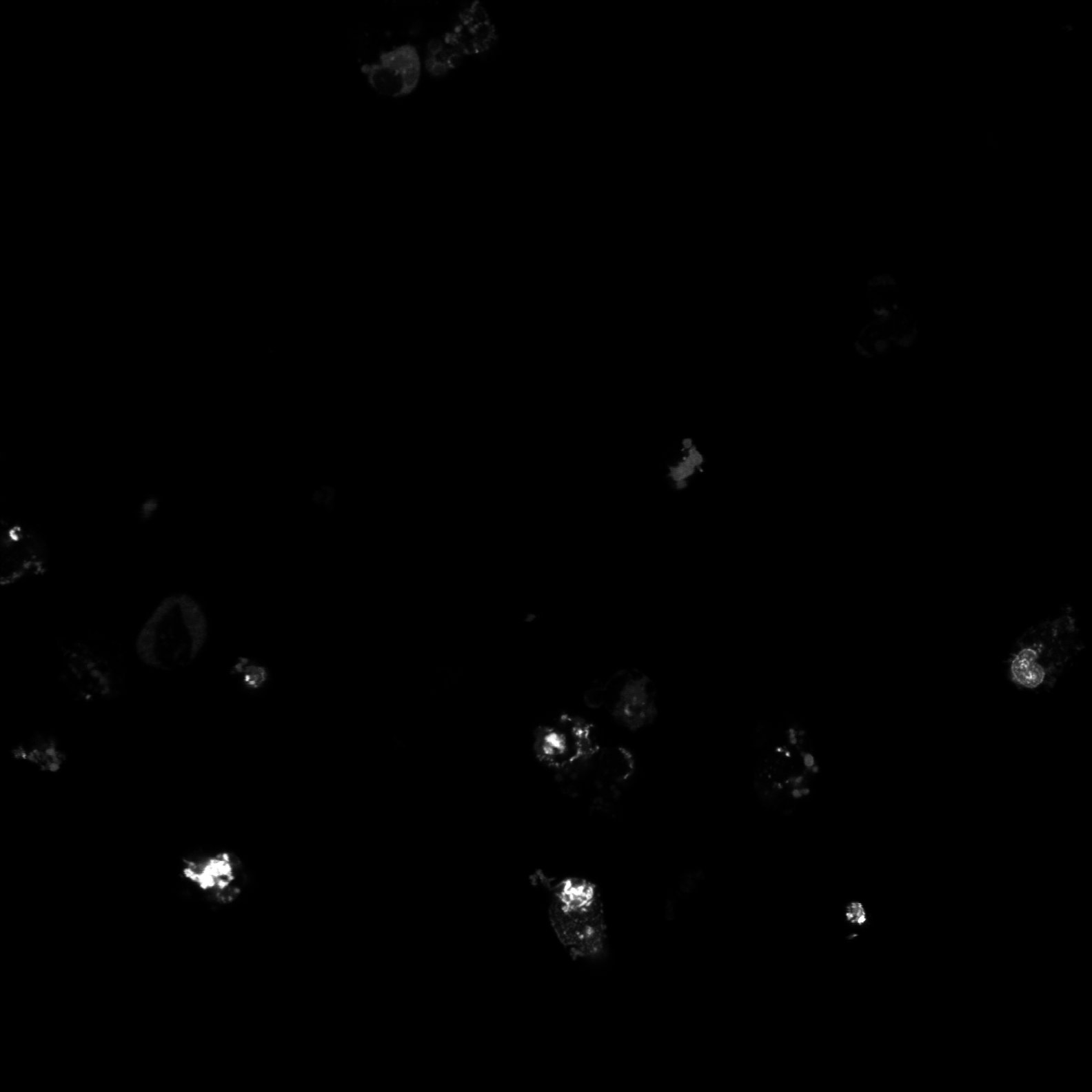

Supplement: Source Data Extended Data Fig. 2 — Unprocessed images. [file 41589_2022_1062_MOESM20_ESM.zip › e-0891_HeLa-Kyoto_FBL_lysate04.jpg]

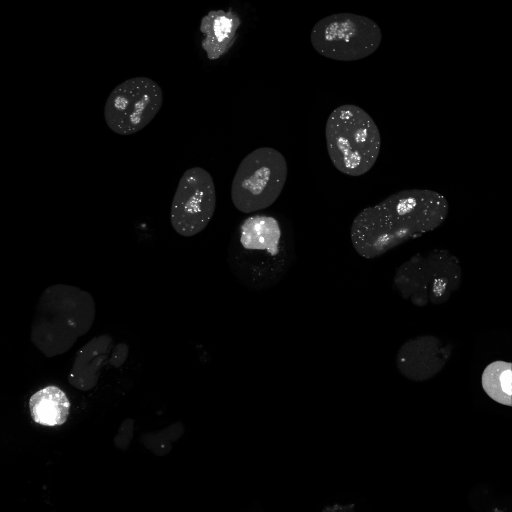

Supplement: Source Data Extended Data Fig. 2 — Unprocessed images. [file 41589_2022_1062_MOESM20_ESM.zip › e-0891_HeLa-Kyoto_NOP.jpg]

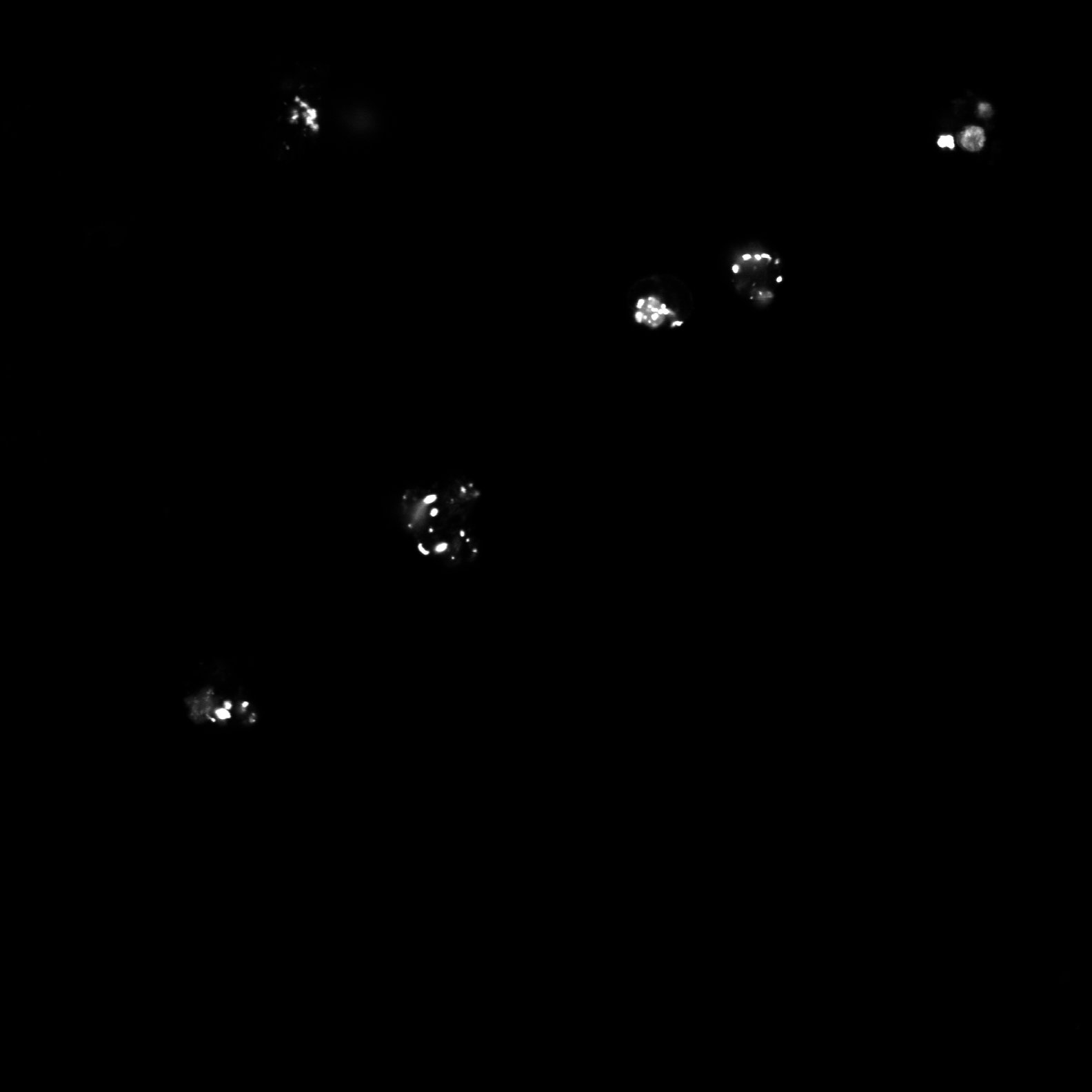

Supplement: Source Data Extended Data Fig. 2 — Unprocessed images. [file 41589_2022_1062_MOESM20_ESM.zip › e-0891_HeLa-Kyoto_NOP_lysate04.jpg]

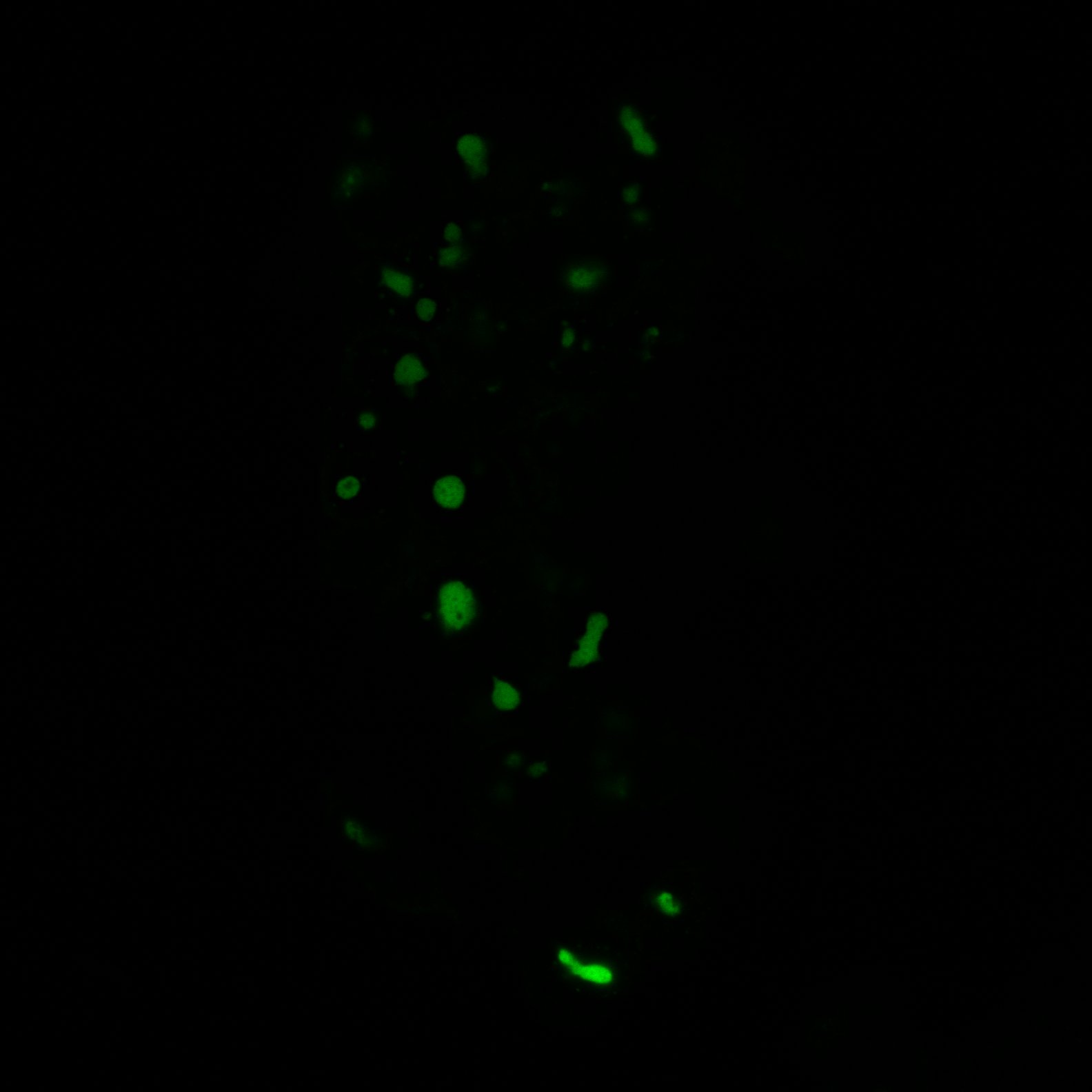

Supplement: Source Data Extended Data Fig. 2 — Unprocessed images. [file 41589_2022_1062_MOESM20_ESM.zip › e-0891_HeLa-Kyoto_NPM1_lysate05.jpg]

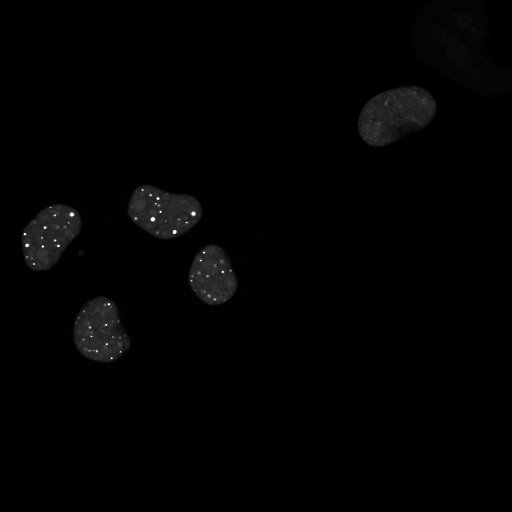

Supplement: Source Data Extended Data Fig. 2 — Unprocessed images. [file 41589_2022_1062_MOESM20_ESM.zip › e-0891_HeLa-Kyoto_PRPF.jpg]

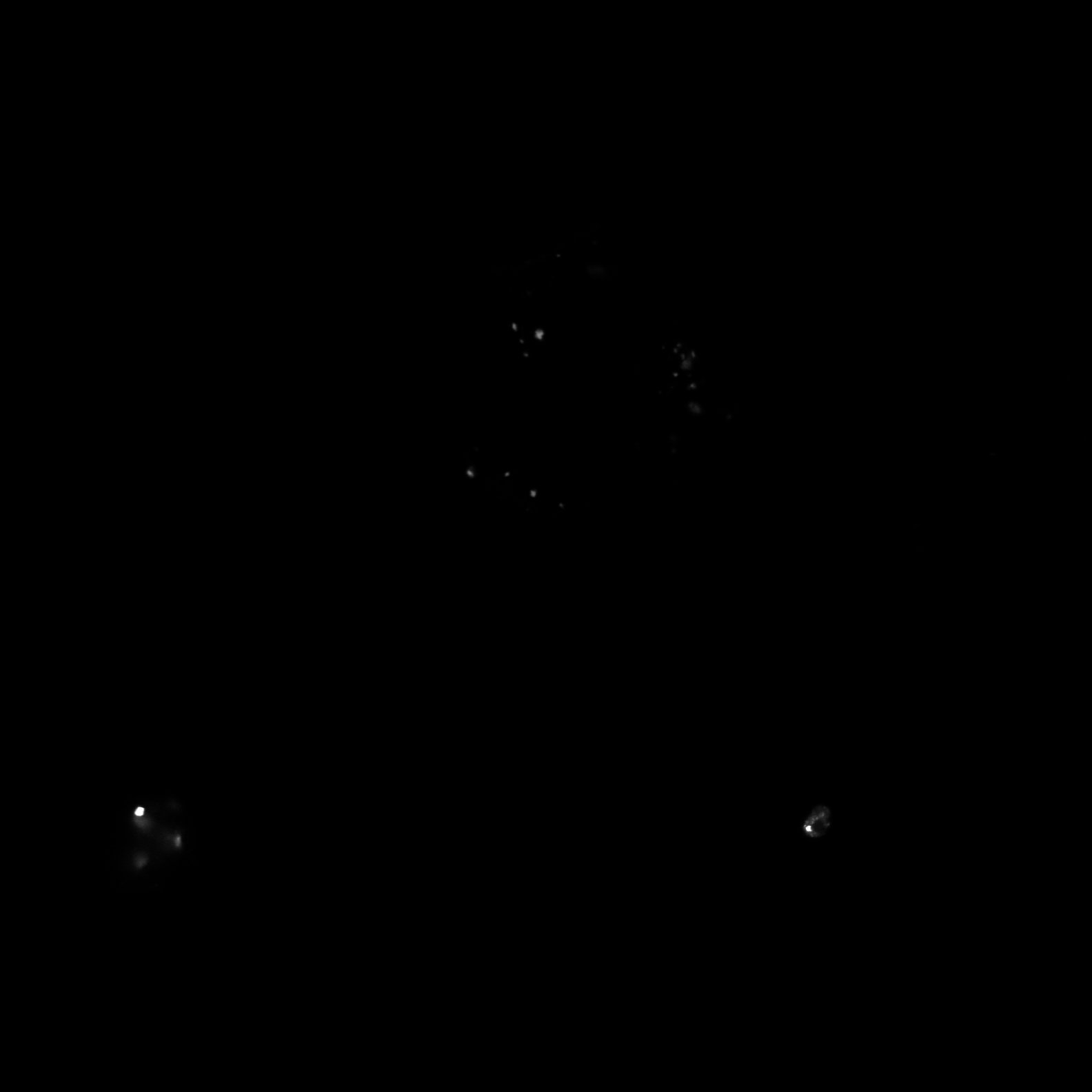

Supplement: Source Data Extended Data Fig. 2 — Unprocessed images. [file 41589_2022_1062_MOESM20_ESM.zip › e-0891_HeLa-Kyoto_PRPF_lysate03.jpg]

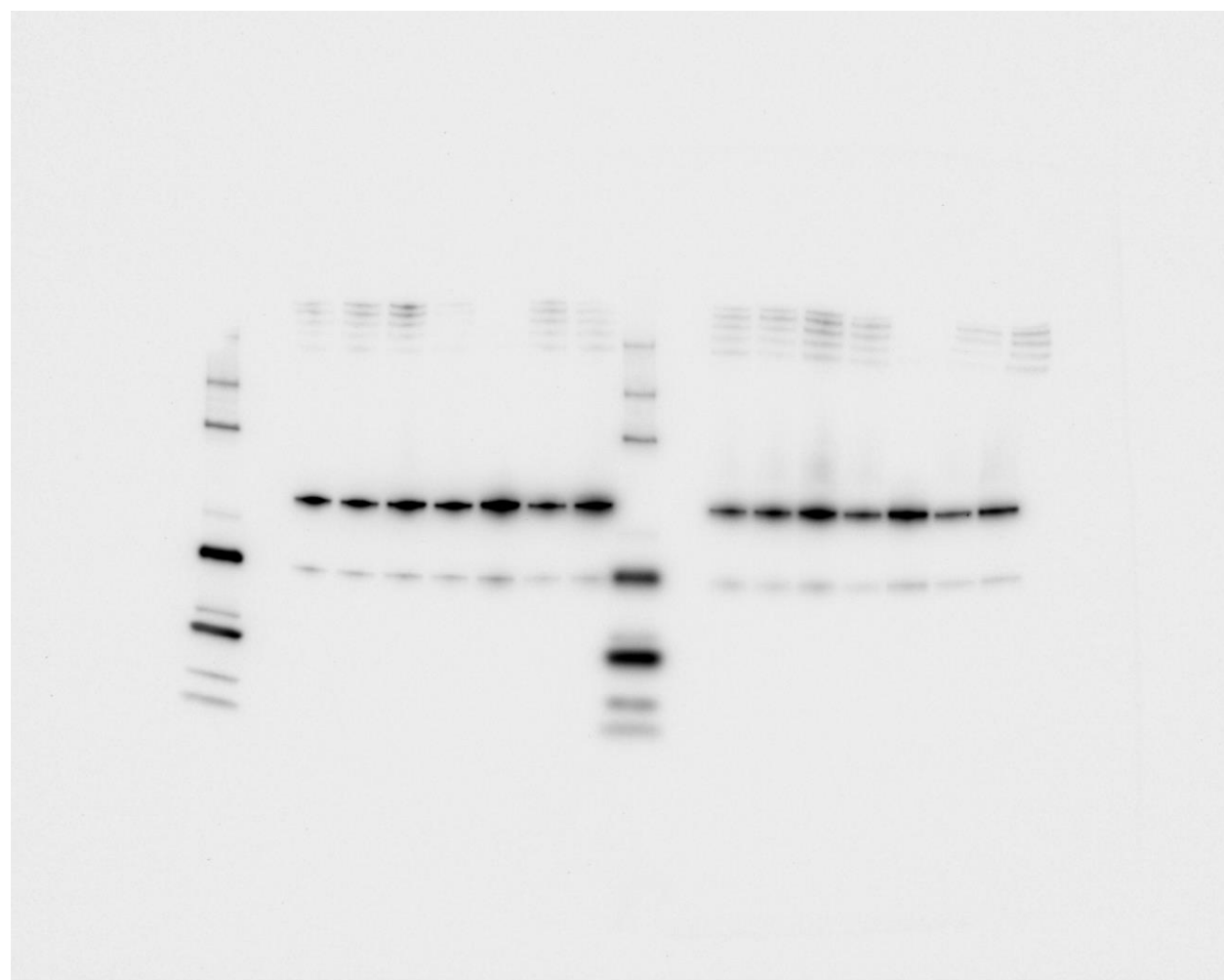

Supplement: Source Data Extended Data Fig. 8 — Unprocessed gel image. [file 41589_2022_1062_MOESM27_ESM.pdf]
